# Identification of Proteasome Subunit Beta Type 6 (PSMB6) Associated with Deltamethrin Resistance in Mosquitoes by Proteomic and Bioassay Analyses

**DOI:** 10.1371/journal.pone.0065859

**Published:** 2013-06-10

**Authors:** Linchun Sun, Yuting Ye, Haibo Sun, Jing Yu, Li Zhang, Yan Sun, Donghui Zhang, Lei Ma, Bo Shen, Changliang Zhu

**Affiliations:** 1 Department of Pathogen Biology, Nanjing Medical University, Nanjing, Jiangsu, P. R. China; 2 Pediatric Research Center, Nanjing Children's Hospital Affiliated to Nanjing Medical University, Nanjing, Jiangsu, P. R. China; 3 Jiangsu Province Hospital on Integration of Chinese and Western Medicine, Nanjing, Jiangsu, P. R. China; Universidade Federal do Rio de Janeiro, Brazil

## Abstract

Deltamethrin (DM) insecticides are currently being promoted worldwide for mosquito control, because of the high efficacy, low mammalian toxicity and less environmental impact. Widespread and improper use of insecticides induced resistance, which has become a major obstacle for the insect-borne disease management. Resistance development is a complex and dynamic process involving many genes. To better understand the possible molecular mechanisms involved in DM resistance, a proteomic approach was employed for screening of differentially expressed proteins in DM-susceptible and -resistant mosquito cells. Twenty-seven differentially expressed proteins were identified by two-dimensional electrophoresis (2-DE) and mass spectrometry (MS). Four members of the ubiquitin-proteasome system were significantly elevated in DM-resistant cells, suggesting that the ubiquitin-proteasome pathway may play an important role in DM resistance. Proteasome subunit beta type 6 (*PSMB6*) is a member of 20S proteasomal subunit family, which forms the proteolytic core of 26S proteasome. We used pharmaceutical inhibitor and molecular approaches to study the contributions of *PSMB6* in DM resistance: the proteasome inhibitor MG-132 and bortezomib were used to suppress the proteasomal activity and siRNA was designed to block the function of *PSMB6*. The results revealed that both MG-132 and bortezomib increased the susceptibility in DM-resistant cells and resistance larvae. Moreover, *PSMB6* knockdown decreased cellular viability under DM treatment. Taken together, our study indicated that *PSMB6* is associated with DM resistance in mosquitoes and that proteasome inhibitors such as MG-132 or bortezomib are suitable for use as a DM synergist for vector control.

## Introduction

Mosquito-borne diseases, such as malaria, dengue fever, yellow fever, filariasis and encephalitis, cause severe mortality and morbidity around the world, and pose significant threats to public health [1]–[4]. For a long period of time, insecticides have been the primary method for managing mosquito-borne diseases [5], [6]. Pyrethroid, one of the most prevalent insecticides, interacts with ion channels, which disrupt the transmembrane potentials, and damage the insect nervous system [7]. Deltamethrin (DM), a representative synthetic pyrethroid insecticide, is widely used for bed net impregnation and residual spraying for mosquito control [8], [9]. Widespread and improper use of insecticides has induced the development of insecticide resistance [10], [11], which has become the main obstacle for the mosquito-borne disease management [12]–[14].

Insecticide resistance is polygenic inheritance phenomenon, which suggests that multiple genes are associated with resistance [15]. Large-scale transcriptional gene expression profiling based on suppression subtractive hybridization (SSH) and cDNA microarray studies had been carried out to identify DM resistance-associated genes in *Culex pipiens pallens* [16], [17]. Although novel genes associated with DM resistance have been identified, the mechanisms underlying DM resistance are still not fully understood. Proteomics research, a strategy focusing on protein expression profiling, has great advantages in the study of complicated biological events [18]. Therefore, characterization and comparison of the protein profiles of susceptible and resistant strains will provide valuable information regarding the resistance mechanisms in mosquitoes.

The ubiquitin-proteasome system plays an essential role in cell cycle regulation, stress, carcinogenesis, and DNA repair, through influencing protein stability [19]–[24]. The ubiquitin-conjugating system generated polyubiquitinated proteins serve as substrates for the proteasome [25], [26]. In brief, proteins modified through ubiquitin-activating enzyme E1, ubiquitin-conjugating enzyme E2, and ubiquitin-protein ligase E3 are targeted by the 26S proteasome for degradation [27].

The proteasome 20S core is a multi-subunit protein complex comprised of two α-rings and two β-rings, which have regulatory activity and proteolytic activity, respectively [28], [29]. The 20S proteasome is responsible for the breakdown of shortlived proteins involved in cellular apoptosis, DNA repair, endocytosis, cell cycle regulation and for the rapid removal of misfolded proteins [30]. *PSMB6* belongs to the 20S proteasomal subunit family, which participates in catalyzing ubiquitin-protein degradation [31].

This study was designed to isolate differentially expressed proteins identified by protein profiling of DM-susceptible and -resistant mosquito cells, and to study the role of *PSMB6* in mosquito deltamethrin resistance.

## Materials and Methods

### 2.1 Cell Culture and Mosquito Strains

*Aedes albopictus* C6/36 cells were obtained from the China Center for Type Culture Collection (Wuhan, China). The DM-resistant C6/36 strains were selected with increasing dose of DM for hundreds of generations. The resistant strains had a 14.8-fold increase in the 50% lethal concentration (LC50) value compared with the DM-susceptible C6/36 strains. Cells were cultured in DMEM/High Glucose media (Hyclone; UT, USA) including 1% penicillin-streptomycin (Gibco; Carlsbad, USA) and 10% fetal bovine serum (Sijiqing; Zhejiang, China) in a 5% CO_2_-humidified incubator at 28°C.

A DM-susceptible strain of *Cx. pipiens pallen* (strain 01, the LC50 was 0.008 mg/L) which had never been exposed to insecticides, was obtained from the Shanghai Insect Institute of the Chinese Academy of Sciences (Shanghai, China). The DM (Sigma; St. Louis, USA) concentration used for selection was determined by LC50, which was calculated by larval bioassays. Two DM-resistant strains (strain 03 and 07) were used in this study. Strain 03 were selected by 0.05 mg/L DM for every generation and strain 07 were selected with increasing dose of DM for generations. For MG-132-treatment (Santa Cruz; Santa Cruz, USA) experiment, mosquitoes were subjected to DM selection for more than 10 generations, the LC50 for strain 03 and strain 07 were 0.08 mg/L and 0.56 mg/L, respectively. For bortezomib-treatment (LC Laboratories; Woburn, USA) experiment, mosquitoes were subjected to DM selection for more than 40 generations, the LC50 for strain 03 and strain 07 were 0.74 mg/L and 3.8 mg/L, respectively. All strains were maintained with approximately 14 h:10 h light/dark cycle at 28°C.

### 2.2 Two-Dimensional Gel Electrophoresis

DM-susceptible and -resistant cells were lysed in buffer containing 7 M urea, 65 mM dithiothreitol (DTT), 2% (v/v) IPG buffer (pH 3–10, nonlinear), 4% (w/v) 3-[(3-cholamidopropyl)-dimethylammonio]-1-propane sulfonate (CHAPS) (GE Healthcare; Uppsala, Sweden), 2 M thiourea and 1% (v/v) protease inhibitor cocktail (Roche; Rockford, USA). The extracts were centrifuged at 50,000×g for 1 h, and the supernatants were stored at −80°C. The Bradford method was used for protein concentration quantification with BSA as the standard [32]. Sample proteins (120 µg) were loaded by gel rehydration on a 24-cm immobilized (pH 3–10) nonlinear gradient strips for 2-DE. Electrophoretic separation was performed under the following conditions: step-n-hold, 30 V for 6 h and 60 V for 6 h, gradient:500 V for 1 h, 1000 V for 1 h, 3000 V for 3 h and 8000 V for 3 h, step-n-hold: 8000 V for 20 h. Isoelectric focusing (IEF) was performed using IPGphor apparatus (Amersham Bioscience, Piscataway, USA).

An Ettan DALTsix system (GE Healthcare, San Francisco, CA, USA) was applied for the second-dimension gel separation [33]. Silver staining was carried out based on a published protocol [34] except that glutaraldehyde was not included in the sensitizing solution. Gels were scanned and analyzed using ImageMaster 2D Platinum Software (GE Healthcare, San Francisco, CA, USA) as previously described [35]. Statistically significant differences were evaluated with the Student's *t*-test (ImageMasterTM 2D platinum software, *P<0.05).

### 2.3 In-Gel Tryptic Digestion and MALDI-TOF/TOF

Silver-stained protein spots were excised, dehydrated in acetonitrile, and dried at room temperature. Proteins were reduced with 10 mM DTT and 25 mM ammonium bicarbonate (NH_4_HCO_3_) at 56°C for 1 h and 55 mM iodoacetamide and 25 mM NH_4_HCO_3_ at RT for 45 min in the dark. Then gel pieces were completely washed with 25 mM NH_4_HCO_3_, 50% acetonitrile and 100% acetonitrile in succession and were thoroughly dried using Speedvac (Concentrator 5301, Eppendorf, Hamburg, Germany). The dried gel pieces were rehydrated with 2 µl trypsin (Promega, Madison, WI, USA) solution (10 mg/L trypsin in 25 mM NH4HCO3) and incubated at 4°C for 40 min. The excess liquid was discarded, and the gel plugs were incubated at 37°C for 12 h before the reaction was stopped by the addition of trifluoroacetic acid (TFA) (Sigma; St. Louis, USA) at a final concentration of 0.1%.

The extracted peptide mixture was then analyzed by MALDI-TOF mass spectrometry and tandem TOF/TOF mass spectrometry which was carried out on a time-of-flight Ultraflex II mass spectrometer (Biflex; Bruker Daltonics, Germany). Peptide mass maps were acquired in positive ion mode using a SmartBeam solid laser (averaging 800 laser shots per MALDI-TOF spectrum and 800 laser shots per TOF/TOF spectrum). Resolution was 15,000 to 20,000. The spectrum was calibrated by bruker calibration mixtures to a mass tolerance within 0.1 Da [33].

### 2.4 Database Queries and Protein Identifications

The *m*/*z* and resolution for mass spectra were ranging from 700 to 4,000 and 10,000 to 20,000, respectively. Results were analyzed using the FlexAnalysis software (version 2.4, Bruker Daltonik GmbH) with the following parameters: peak detection algorithm, Sort Neaten Assign and Place (SNAP); S/N threshold, 3.0; quality factor threshold, 50. The tryptic autodigestion peptides (842.51 and 1,045.56 Da) were used as internal standards. The matrix or auto-proteolytic trypsin fragments and known contaminants (e.g., keratins) were removed. The search conditions used were as described [36]. The detailed principle is available online (http://www.matrixscience.com/pdf/2003WKSHP2.pdf). Search parameters for MS data: 100 ppm for the precursor ion and 0.3 Da for the fragment ions. Covalent modifications and cleavage specificity were recognized to be the same as those described for peptide mass fingerprint (PMF) analysis. Confidence intervals exceeding 95% were considered significant. All significant MS results identified results by Mascot were manually validated for spectral quality, and y and b ion series matches.

### 2.5 Larvicidal Activity Assay

Three strains of *Cx. pipiens pallen* (strain 01, 03 and 07) were used for these experiments. In each strain, 180 early fourth instar larvae, randomly divided into three groups, were subjected to different treatments. Larvae were pre-treated with 1 µM MG-132 or 0.125 µM bortezomib for 4 h, followed by DM treatment. The LC50 of each strain was used as the test concentration. The larvae were exposed to these solutions at 28°C for a14 h∶10 h light/dark cycle and the percentage of survival was recorded. MG-132 or bortezomib treatment alone and DM treatment alone were applied as control and each assay was repeated three times.

### 2.6 RNA extraction and cDNA synthesis

Total RNA of mosquito cells was extracted by TriZol Reagent (Invitrogen; Carlsbad, USA) and the cDNA was reverse transcribed from 1 µg of total RNA by the SuperScript® VILO™ cDNA Synthesis kit (Invitrogen; Carlsbad, USA), according to the manufacturer's instructions.

### 2.7 Cloning of Cx. pipiens pallen PSMB6 full-length cDNA

Full-length *Cx. pipiens pallen PSMB6* cDNA was amplified in separate reactions covering three different regions: the open reading frame (ORF), 5′-cDNA ends (5′-RACE) and 3′-cDNA ends (3′-RACE) of *PSMB6*. The ORF of *PSMB6* was amplified using the following primers: 5′-GGACTAGTGAGATGGAAATGGACATGGCTACTCAAACGACT-3′ (sense) and 5′-CCCTCGAGCTAAGCACGAACGGCGACC-3′ (antisense), designed based on its homologue *Cx. quinquefasciatus PSMB6* (GenBank No. XM_001846164.1). PCR reactions were accomplished using KOD-Plus-Neo-401 (TOYOBO; Doushima Hama, Japan) according to the manufacturer's instructions. Amplification of the 5′-RACE and 3′-RACE of *PSMB6* was carried out using a SMART™ RACE cDNA Amplification Kit (Clontech; Mountain View, USA). The sequences of 5′-RACE and 3′-RACE adaptor primers supplied by the SMART™ RACE cDNA Amplification Kit were: 5′-CTAATACGACTCACTATAGGGCAAGCAGTGGTATCAACGCAGAGT-3′ and 5′-CTAATACGACTCACTATAGGGC-3′, respectively. The specific primer sequences for 5′-RACE and 3′-RACE were: 5′-TGTGGTGGGCGTTTCTCCAGTCGT-3′ and 5′-CCATTGGAGGTTCGGGAAGTTCGTACA-3′, respectively. The PCR reactions were carried out using an Advantage®2 PCR Kit (Clontech; Mountain View, USA) following the manufacturer's instructions. PCR products were separated by 1% agarose gel electrophoresis and purified using a QIA quick Gel extraction kit (QIAGEN; GmbH, Germany). The purified products were sequenced by the Shanghai Invitrogen Biotechnology Company (Shanghai, China). Finally, all sequences were assembled to generate the putative full-length cDNA of *PSMB6*.

### 2.8 Sequence Alignment and Phylogenetic Analysis

The standard protein/protein BLAST sequence comparison programs (http://beta.uniprot.org/?tab=blast) were used to search sequences with similarities to the translated sequences of *PSMB6* in the SWISS-PROT databases. Deduced amino acid sequences were aligned by the ClustalW2 computer program (http://www.ebi.ac.uk/Tools/clustalw2/index.html). The MEGA 5.0 program was used to construct the phylogenic tree.

### 2.9 Quantitative PCR analysis

Quantitative PCR assays were performed with the ABI PRISM 7300 equipment (Applied Biosystems, Foster City, USA). The primers used are all listed in Table S1. According to the manufacturer's instruction, each reaction was performed in a total volume of 20 µl containing cDNA, specific forward and reverse primers and LightCycler FastStart DNA Master SYBR Green I (Roche; Rockford, USA). A melting curve was generated immediately after the reaction to check the specificity and the data were analyzed with 7300 System SDS Software v1.2.1 (Applied Biosystems). The parameters for PCR were set as: 95°C for 30 s, followed by 40 cycles of 94°C for 30 s, 55°C for 30 s, and 72°C for 30 s, and the dissociation curve was inspected for quality control purposes. *β-actin* was used as the internal control. The relative gene expression level was calculated from the threshold cycle (C_t_) value of each reaction through Delta-delta Ct method [37].

### 2.10 Construction of PSMB6-siRNA

*Ae. albopictus PSMB6* (GenBank No. GU_371441.1) was amplified with two degenerate primers, which were designed based on the conserved *PSMB6* segment of *Cx. quinquefasciatus* and *An. gambiae*. Double stranded siRNA molecules corresponding to the partial *PSMB6* cDNA sequence were designed and synthesized by the GenePharma Company (Shanghai, China). The sequences of *PSMB6* siRNA and scrambled siRNA (used as a negative control) were as follows: *PSMB6*-siRNA, 5′-UGGCCGGAUUUGAUAACAATT-3′ (sense) and 5′-UUGUUAUCAAAUCCGGCCATT-3′ (antisense); and scrambled siRNA, 5′ –GCGACGAUCUGCCUAAGA-3′ (sense) and 5′-AUCUUAGGCAGAUCGUCG-3′ (antisense).

### 2.11 Cell Viability Analysis

Resistant and susceptible cells were seeded (2×10^4^/well) in 100 µl per well of complete media in four 96-well plates and incubated for 24 h. Cells were pre-treated with 1 µM MG-132 or 0.1 µM bortezomib. 1% (v/v) Dimethyl sulfoxide (DMSO) (Sigma; St. Louis, USA) alone was used as a negative control. Cells (resistance and susceptible) were then treated with 100 µl MG-132 or bortezomib for 4 h. Other batches of cell were transfected with *PSMB6*-siRNA for 7 h. siRNA (6 µl) and X-tremeGENE siRNA Transfection Reagent (6 µl) (Roche; Rockford, USA) were mixed in 1.2 ml DMEM in a RNase-free tube for 15 min at room temperature, then 4.8 ml DMEM was added to the transfection mixture, mixed again and added to cells (100 µl per well). Cells transfected with scrambled siRNA were used as negative controls. Cells were treated with 10^0.5^, 10^1^, 10^1.5^, 10^2^, 10^2.5^ mg/L of DM for 68 h. According to the manufacturer's instructions, CCK-8 reagents (Dojindo; Gaithersburg, USA) was added to the medium and incubated for a further 4 h at 28°C. A microplate reader (Biotek Instruments; Winooski, USA) was used to measure the absorbance at 450 nm. Cell viability was calculated as a percentage based upon control cell viability.

### 2.12 Statistical analysis

All statistical analysis was performed with GraphPad 5.0 (GraphPad Software). Statistically significant differences were evaluated with the Student's *t*-test (*P<0.05, **P<0.01). All experiments were performed in triplicates on at least three separate occasions.

## Results

### 3.1 Characterization of DM-resistant Mosquito Cells

**Figure 1:**
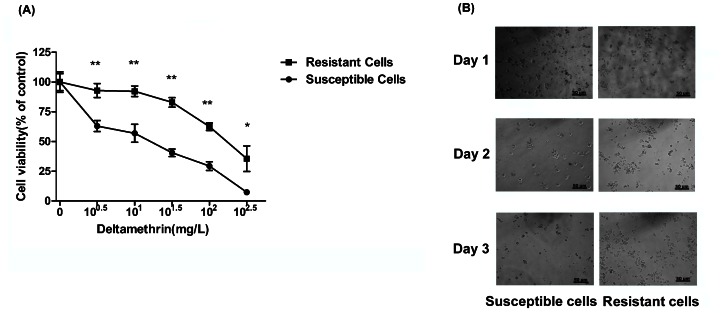
Characterization of DM-resistant mosquito cells. (A) Resistant and susceptible cells were treated with DM at the indicated concentrations, and cell viability was measured after 72 h treatment. The percentage of viable cell is shown relative to the control (DMSO only). The results shown are representative of three independent experiments. After treatment with 50 mg/L DM for 72 h, representative images (B, 200×) of susceptible- and resistant-cells were taken.

DM-resistance mosquito cells were selected with increasing doses of DM for hundreds of generations to generate the DM-resistance cells and cell viability was analyzed using a modified MTT method (CCK-8). As shown in Figure 1, after DM selection, the LC_50_ for the resistant cells was 251 mg/L, much higher than 17 mg/L for the susceptible cells (Figure 1A). DM-selection in resistance cells significantly improved cell viability and proliferation compared with susceptible cells (Figure 1B).

### 3.2 Identification and quantification protein spots on 2-DE gels

**Figure 2:**
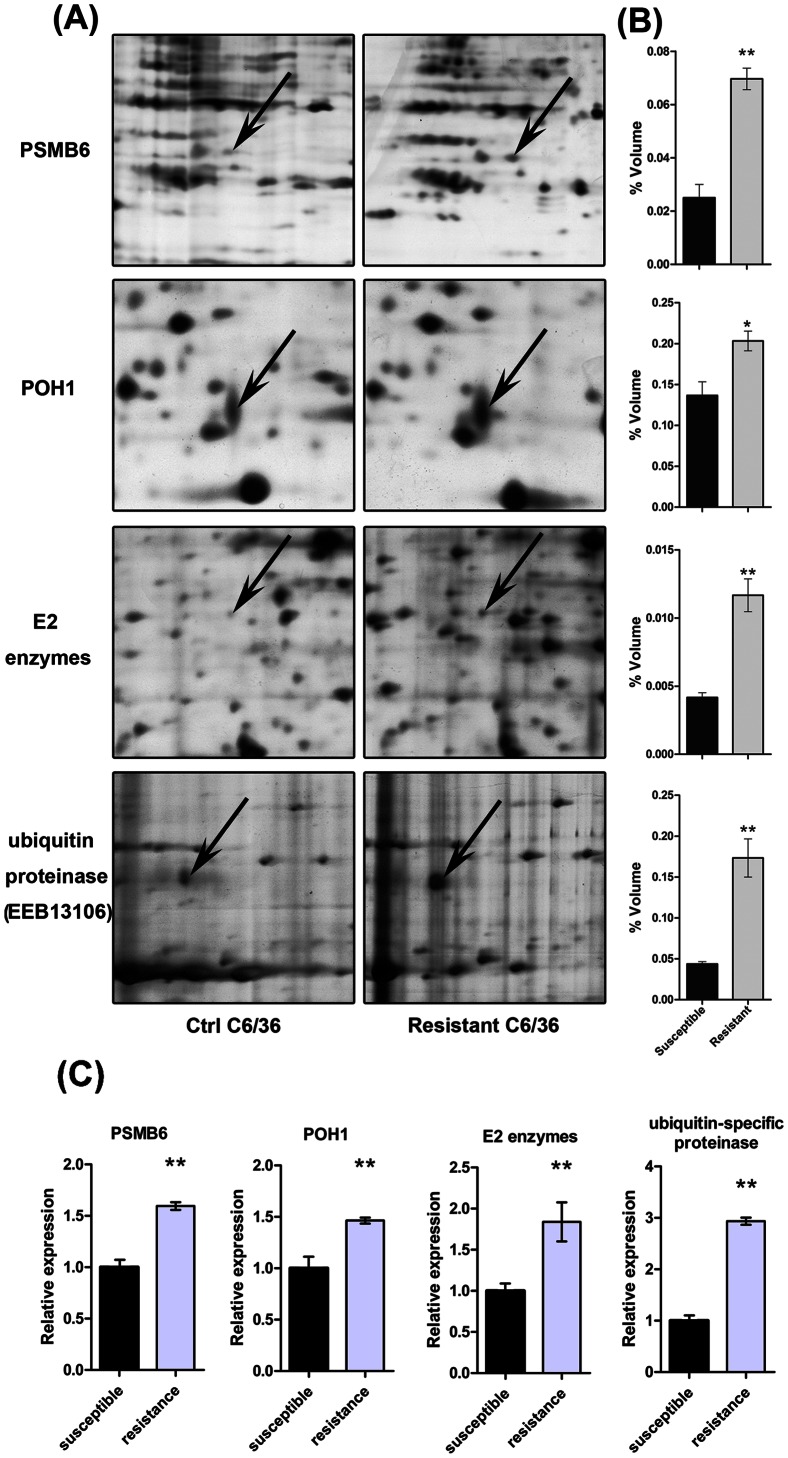
Four unbiquitin-proteasome proteins were up-regulated in DM-resistant mosquito cells. (A) Representative magnified images of 2-D gel showing the elevated expression of proteins (*PSMB6*, *PSMD14* (*POH1*), *E2* enzymes and *USP*) in DM-resistant cells. (B) Relative expression levels of the four ubiquitin-proteasome proteins in DM-resistant cells. Spot density was digitized and the results are shown as the mean±SEM of three independent experiments. *P<0.05, **P<0.01 compared with the DM-susceptible cells. (C) Levels of *PSMB6*, *POH1*, *E2* and *USP* mRNA in mosquito strain 07 and strain 01. The relative expression rate was determined by quantitative PCR and normalized with β-actin. Results are expressed as the mean±SEM. *P<0.05, **P<0.01 compared with control. The results shown are representative of three independent experiments.

A representative 2-DE gel image for protein expression of DM-resistant and -susceptible mosquito cell is presented in Figure S1. Thirty-six proteins were identified to be significantly differently expressed between two groups (*P*<0.05 with average spot intensity greater than 1.2-fold). All spots were subjected to tryptic digestion and MALDI-TOF/MS analysis. Using PMF analysis, twenty-seven proteins were identified (summary of these proteins, including accession numbers, protein names, scores, sequence coverage, Mr, and pI, are listed in Table 1 and the raw data of each protein are shown in Table S2). The other nine proteins were unidentified because of incomplete polypeptide fragments or low abundance. A further magnified 2-DE gel image of four DM-resistant upregulated proteins: proteasome subunit beta type 6 (*PSMB6*), 26S proteasome non-ATPase regulatory subunit 14 (*PSMD14*/*POH1*), ubiquitin-conjugating enzyme (*E2*) and ubiquitin-specific protease, putative (*USP*) was shown in Figure 2. These proteins are components of the ubiquitin-proteasome system. Quantitative PCR confirmed that gene expression of *PSMB6*, *POH1*, *E2* and *USP* was 1.59, 1.46, 1.80 and 2.93-fold increased in DM-resistant mosquito strain (strain 07, LC50 was 3.8 mg/L) compared with those of DM-susceptible strain (strain 01) (Figure 2C).

**Table 1:** The Profile of 27 Proteins Identified by 2-DE.

ID	Acc. no.	Protein description	No. of matched peptides	Score	Sequence coverage(%)	Mr/pI	Fold of resistant/susceptible	*P* value
Up^a^								
253	gi|157125883	40S ribosomal protein S12	13	183	68	15687/6.20	4.23	0.0069
407	gi|94469190	proteasome subunit beta type 6 precursor-like protein	16	163	56	25028/5.45	1.83	0.0023
508	gi|157108608	pap-inositol-1,4-phosphatase	13	99	39	33892/5.79	4.24	0.0034
536	gi|157130038	elongation factor ts	12	69	26	34245/5.94	4.26	0.0007
554	gi|157112126	phosphoglycerate mutase	15	149	51	28595/6.34	2.89	0.0006
583	gi|170043858	26S proteasome non-ATPase regulatory subunit 14	12	114	31	34696/5.74	1.84	0.0315
618	gi|157114501	14-3-3 protein	11	123	43	28324/4.78	5.17	0.0002
672	gi|157134886	aldo-keto reductase	17	165	43	35865/5.20	3.58	0.0192
725	gi|157131170	l-lactate dehydrogenase	18	176	32	35812/6.61	3.32	0.0317
728	gi|94469060	transaldolase	21	169	44	35909/5.45	3.63	0.0170
898	gi|170036819	elongation factor ts	28	69	10	35022/5.88	1.81	0.0032
966	gi|157134639	eukaryotic translation initiation factor 3 subunit	10	86	27	36224/5.56	1.99	0.0335
991	gi|157113904	acetyl-coa acetyltransferase, mitochondrial	11	105	30	43577/8.60	2.27	0.0329
1011	gi|157130317	actin	14	134	33	44980/5.92	2.64	0.0396
1095	gi|157115992	UDP-glucose 4-epimerase	25	228	57	39001/6.46	2.24	0.017
1153	gi|157110699	ethanolamine-phosphate cytidylyltransferase	14	98	26	42278/6.06	3.27	0.0229
1186	gi|157130058	hypothetical protein	8	73	26	44678/5.93	4.13	0.0089
1206^c^	gi|157139358	ubiquitin-conjugating enzyme E2	9	68	57	14187/5.89	5.29	0.0039
	gi|158292977	AGAP004890-PB	13	81	22	45597/7.17		
1236	gi|170054846	mannose-1-phosphate guanylyltransferase	9	67	22	39381/6.68	1.68	0.0435
1348	gi|157116575	chaperonin	9	79	25	53596/5.59	2.36	0.0135
1463	gi|157106871	phenylalanyl-tRNA synthetase beta chain	17	100	28	66523/5.85	2.92	0.0227
1487	gi|157110521	adenylylsulfate kinase	19	93	22	70212/5.87	2.10	0.0360
2235	gi|212509777	ubiquitin specific proteinase 54	21	70	12	191744/8.95	3.62	0.0053
Down^b^								
83	gi|170058335	phosphoglycerate mutase 2	15	132	46	28582/6.62	0.16	0.0012
245	gi|170040378	conserved hypothetical protein	9	67	25	51872/5.55	0.45	0.0062
363	gi|157115772	glial maturation factor	9	72	59	16747/4.92	0.51	0.0040
589	gi|157131660	methylthioadenosine phosphorylase	11	98	44	30776/6.07	0.71	0.0054

Profile of differentially expressed proteins in DM-resistant cells. Acc. no.: Swiss-Prot or TrEMBL database accession number; Protein description: name of protein in the Swiss-Prot or TrEMBL database; No of matched peptides: number of peptides matched to the candidate protein (the number of observed peptides); Score: Mowse Score, scores greater than 67 are considered statistically significant (*p*<0.05); Sequence coverage: identified sequence as a percentage of the complete sequence; Mr: molecular weight; pI: theoretical isoelectric point; Folds of resistant/susceptible: describes the fold changes of the protein expression level in resistant cell compared with that in susceptible cell; *P*-value: Statistical significance of the fold-change in protein expression;

a Up-regulated proteins.

b Down-regulated proteins.

c This protein spot contains more than one protein.

### 3.3 Proteasome inhibitor increases the sensitivity of DM-resistant cells to DM treatment

**Figure 3:**
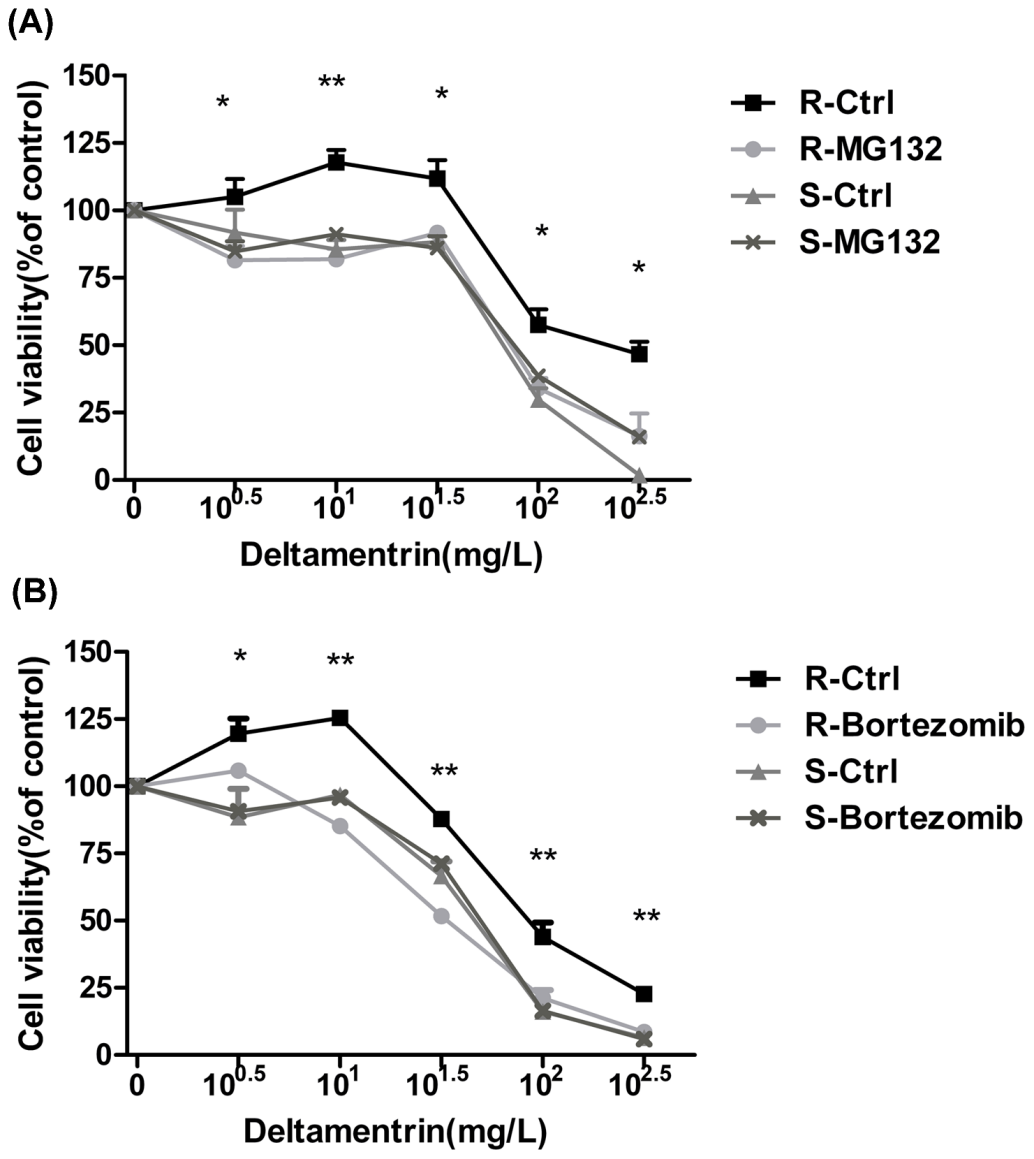
Proteasome inhibitor enhanced cell susceptibility to DM. Resistant and susceptible mosquito cells were exposed to DM at the indicated concentrations after pre-treatment with 1 µM MG-132 (A) or 0.1 µM bortezomib (B) and cell viability was measured. *P<0.05, **P<0.01 compared with the control group. The results shown are representative of three independent experiments.

The proteasome inhibitor MG-132 and bortezomib were used to elucidate the involvement of the ubiquitin-proteasome system in DM resistance. A dose-dependent viability experiment was conducted to select the optimum dose of MG-132 or bortezomib. Viabilities and morphology of DM-resistant cells were not notably affected by MG-132 ranging from 0.125 µM to 1 µM (Figure S2A) or bortezomib ranging from 0 µM to 2 µM (Figure S2B). 1 µM and 0.1 µM were chosen as the optimal concentrations for MG-132 and bortezomib, respectively. DM treatment decreased cell viability in a dose-dependent manner, while MG-132(Figure 3A) or bortezomib (Figure 3B) pre-treatment sensitized DM-resistant cells to DM compared with the controls. However, MG-132 or bortezomib treatment has no significant effect on DM-susceptible cell.

### 3.4 Proteasome inhibitor increases mosquito larvae sensitivity to DM

**Figure 4:**
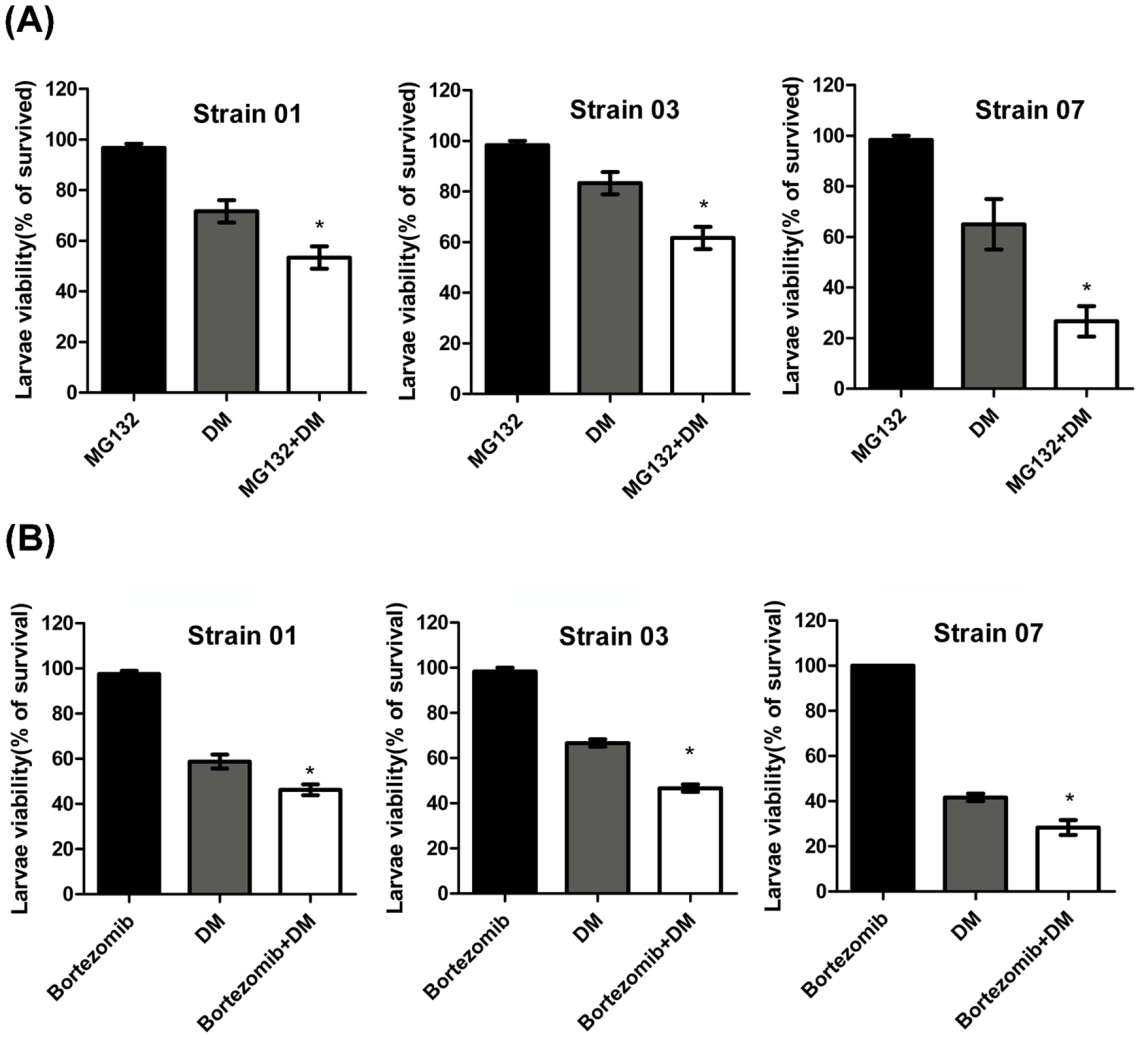
Proteasome inhibitor enhanced susceptibility to DM in mosquito larvae. Three different DM resistance levels strains (strains 01, 03 and 07) were used in these experiments. Larvae with different levels of DM resistance levels were exposed to the indicated concentrations of DM for 24 h after pre-treatment with 1 µM MG-132 (A) or 0.125 µM bortezomib (B), and the viability was evaluated. *P<0.05, **P<0.01 compared with the DM group. The results shown are representative of three independent experiments.

Three *Cx. pipiens pallen* strains (see Section 2.1) were used in these experiments, 1 µM was used as the optimal concentration for MG-132 (Figure S2C) and 0.125 µM for bortezomib (Figure S2D) (identified in dose-escalation experiments) in larvicidal activity assays. DM treatments reduced mosquito larvae viability in all three strains. MG-132 or bortezomib pre-treatment significantly decreased the mosquito larvae viability compared with the DM treated group in all strains (MG-132: Strain 01, 53% vs. 72%; Strain 03, 62% vs. 83%; Strain 07, 27% vs. 65%; *P*<0.05 (Figure 4A); Bortezomib: Strain 01, 46% vs. 58%; Strain 03, 47% vs. 67%; Strain 07, 28% vs. 41%; *P*<0.05 (Figure 4B)), which indicate that MG-132 or bortezomib treatment make mosquitoes more susceptible to DM treatment.

### 3.5 Molecular cloning and sequence analysis of PSMB6

*PSMB6* is a catalytic subunit of proteasome β-rings [25], [26]. The full-length cDNA of *PSMB6* from *Cx. pipiens pallen* were cloned and submitted to GenBank (GenBank Accession NO: JQ037858) (Figure S3A). The deduced peptide is composed of 227 amino acids. Homology analysis of the *Cx. pipiens pallen PSMB6* sequence revealed 99% and 91% and 90% identity with *PSMB6* of *Cx. Quinquefasciatus*, *Ae. aegypti* and *An. Gambiae*, respectively (Figure S3B). Phylogenetic analysis showed that *Cx. pipiens pallen*, *Cx. quinquefasciatus*, *Ae. albopictus*, *Ae. aegypti* and *An. gambiae* share the most recent common ancestry (Figure S3C).

### 3.6 PSMB6 is essential for DM resistance in mosquito cells

**Figure 5:**
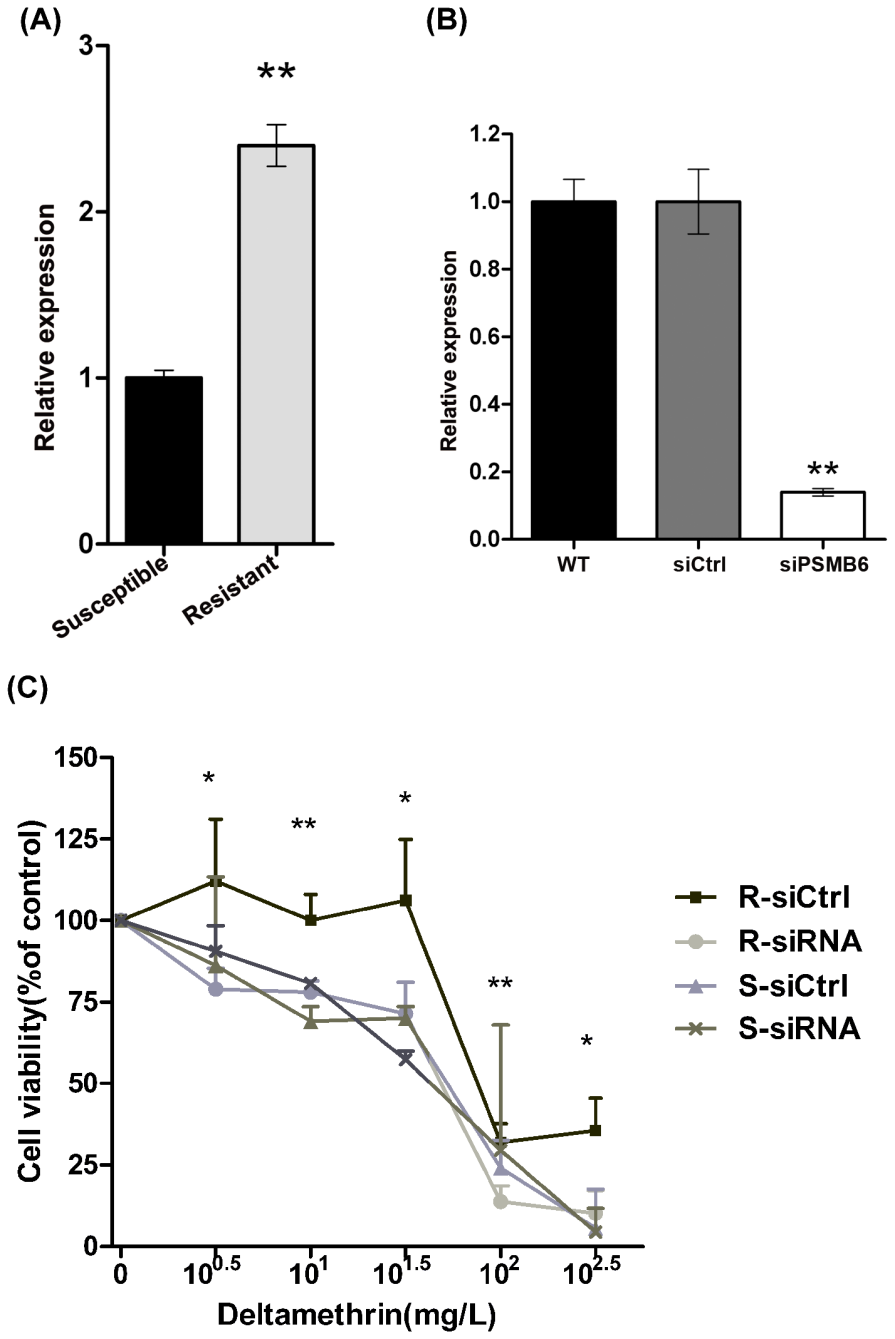
*PSMB6* played an important role in DM resistance. (A) Levels of *PSMB6* mRNA in DM-susceptible and -resistant mosquito cells. The relative expression rate was determined by quantitative PCR and normalized with β-actin. Results are expressed as the mean±SEM. **P<0.01 compared with the control. The results shown are representative of three independent experiments. (B) The knockdown efficiency of *siPSMB6*. DM-resistant mosquito cells were transfected with *siPSMB6* or a scrambled siRNA as a control. Results are expressed as the mean±SEM. **P<0.01 compared with the WT and siCtrl groups. (C) Transient knockdown of *PSMB6* decreased DM resistance in resistant mosquito cells. DM-resistant and -susceptible cells were transfected with *siPSMB6* and siCtrl and exposed to DM at the indicated concentrations. Cell viability was measured after 72 h. The percentage of viable cells is shown relative to the control (DMSO only). *P<0.05, **P<0.01 compared with the DM-susceptible group.

The expression of *PSMB6* in DM-resistant cell was shown to be 2.5-fold higher than that in DM-susceptible cell assessed by quantitative PCR (Figure 5A). Combined with the 2-DE results, these results indicated that *PSMB6* expression is upregulated at both the transcriptional and translational levels in DM-resistant cells. Subsequent investigation of the role of *PSMB6* in DM resistance was conducted using *PSMB6* siRNA. The knockdown efficiency of *PSMB6* was confirmed by quantitative PCR (Figure 5B). Cell viability over a wide range of DM concentrations (10^0.5^, 10^1^, 10^1.5^, 10^2^, 10^2.5^ mg/L) of DM was measured using the CCK-8 method. As shown in Figure 5C, transient knock down of *PSMB6* expression in DM-resistant cells resulted in decreased cell viability, indicating that cell lack of *PSMB6* became more sensitized to DM treatment. No significant difference in cell viability was observed between control and *PSMB6* siRNA-treated groups of DM-susceptible cells.

## Discussion

In the present study, a global comparative proteomic analysis was performed between DM-susceptible and -resistant mosquito cells. Twenty-seven DM resistance-associated candidate proteins, involved in metabolism, energy generation, translation and signalling transduction, were identified. Four proteins (*PSMB6*, *POH1*, *E2* enzymes and *USP*) involved in different steps of ubiquitin-proteasome degradation process were found to be upregulated. Together with the upregulated transcription of these genes, our results suggested that ubiquitin-proteasome system may play critical roles in DM-resistance.

*E2* is involved in the formation of ubiquitin chains during protein ubiquitination. Ubiquitin specific proteinase (*USP*) is an important regulator that mediates the removal, and recycling of ubiquitin to maintain adequate free ubiquitin levels [38]–[40]. *POH1* is a component of the proteasomal complex [41]. *PSMB6* is a member of the proteasome *β*-type family, which participate in the formation of proteolytic centers of proteasome machinery [42]. The upregulated expression of these ubiquitin-proteasome proteins involved in each stage of the degradation process suggests that this ubiquitin-proteasome system is functionally enhanced and may contribute to DM resistance in mosquitoes.

Enhanced proteasomal activity has been demonstrated as a mediator of resistance to chemotherapy [20]. Overexpression of members of the ubiquitin-proteasome pathway has been implicated in cancer chemotherapy resistance. Inhibiting E2 enzyme activity with CDC34 could enhance the anti-cancer activity of bortezomib, dexamethasone and 2-methoxyestradiol [43]. Smith et al (2007) reported that the over-expression of the *PSMB1* proteasomal subunit is associated with resistance to cisplatin in cancer cell lines [44]. Furthermore, *PSMB7* has been proved to be associated with anthracycline-resistance in breast cancer [45]. Overexpression of the *POH1* subunit confers resistance to vinblastine, cisplatin, doxorubicin and paclitaxel in mammalian cells [43]. In this study, we identified *PSMB6* as a DM-resistance mediator in mosquitoes for the first time.

MG-132 and Bortezomib are both reversible and cell-permeable proteasome inhibitor [46]–[48]. Bortezomib is reported to be more specific with no significant inhibitory activity towards other enzymes or receptors [48]. As we anticipated, pre-treatment with MG-132 or bortezomib was associated with significantly decreased viability of DM-resistant cells or mosquito larvae indicating that proteasome do involved in DM-resistant. *PSMB6* was highly at transcriptional level in DM-resistant cells, which was consistent with the protein profile, indicating that transcriptional upregulation of *PSMB6* leads to translational upregulation of the protein. *PSMB6* silencing resulted in significantly decreased cell viability under DM stress. Inhibition of proteasome activity using pharmaceutical inhibitor or knocking down the expression of *PSMB6* through molecular methods resulted in sensitization of mosquito cells to DM-treatment, which strongly suggests that ubiquitin-proteasome system maybe involved in the DM resistance. Taken together, it is possible to manage DM resistance by regulating the ubiquitin-proteasome activity.

Under continuous selective pressure from insecticides, mosquitoes have attained stable inheritance of DM-resistance through over-expression of ubiquitin-proteasome proteins. It can be speculated that hyperactivation of the proteasome degradation pathway is associated with pyrethroid resistance, although further studies are required to elucidate the underlying mechanism.

This study provides compelling evidences that *PSMB6* is associated with DM resistance, which indicated the potential of proteasome inhibitors as synergistic agent for insecticides.

## Supporting Information

Figure S1 **Representative 2-DE images of susceptible and resistant mosquito cell lysates.** The 27 differential protein spots identified by MS are marked with arrows.
(TIF)

Figure S2 **Effect of proteasome inhibitors on cell or larvae viability.** DM-resistant mosquito cells were treated with MG-132 (A) or bortezomib (B) at the indicated concentrations for 72 h and the cell viability was measured by CCK-8 assay. Results are expressed as the mean±SEM. *P<0.05, **P<0.01 compared with the DMSO control. Larvae of the early fourth instar were exposed to MG-132 (C) or bortezomib (D) at the indicated concentrations for 24 h before the survival was calculated. The results shown are representative of three independent experiments.
(TIF)

Figure S3 **Molecular cloning and sequence analysis of** ***PSMB6*** **.** (A).Three fragments of the cDNA sequence of *PSMB6*. The PCR product was cloned from *Cx. pipiens pallen* and separated by electrophoresis. (B) Homology analysis of *PSMB6* cloned from *Cx. pipiens pallen*. (C) Phylogenetic relationship of *PSMB6* with other species.
(TIF)

Table S1 **Sequences of primers used for quantitative PCR.**
(DOC)

Table S2 **The MALDI-TOF-MS raw data for all peptides (data used to generate** **Table 1** **).**
(DOC)
