# Supplementary figures and images for: Identification of Proteasome Subunit Beta Type 6 (PSMB6) Associated with Deltamethrin Resistance in Mosquitoes by Proteomic and Bioassay Analyses
Source: PLoS One. 2013 Jun 10;8(6):e65859. doi: 10.1371/journal.pone.0065859 (PMC3677870; doi:10.1371/journal.pone.0065859)

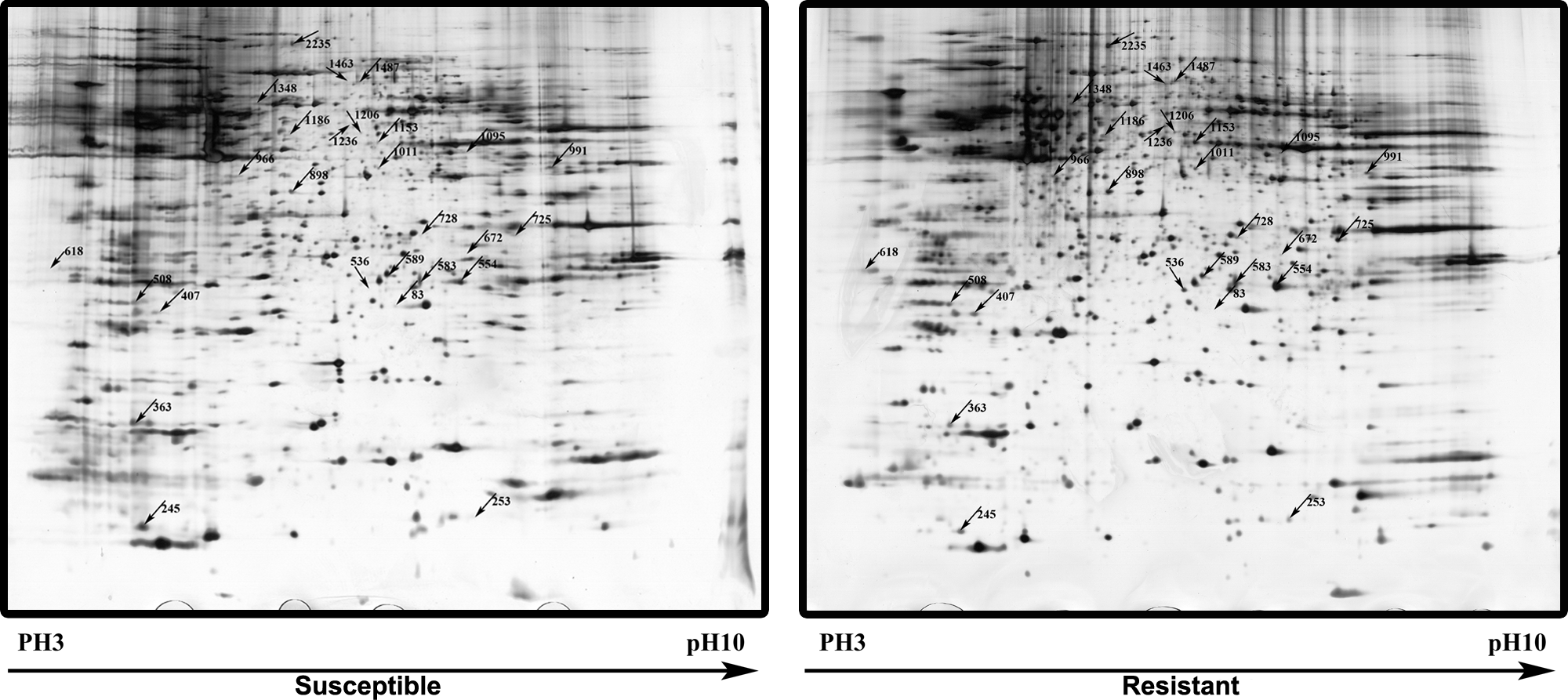

Supplement: Figure S1 — Representative 2-DE images of susceptible and resistant mosquito cell lysates. The 27 differential protein spots identified by MS are marked with arrows. (TIF) [file pone.0065859.s001.tif]

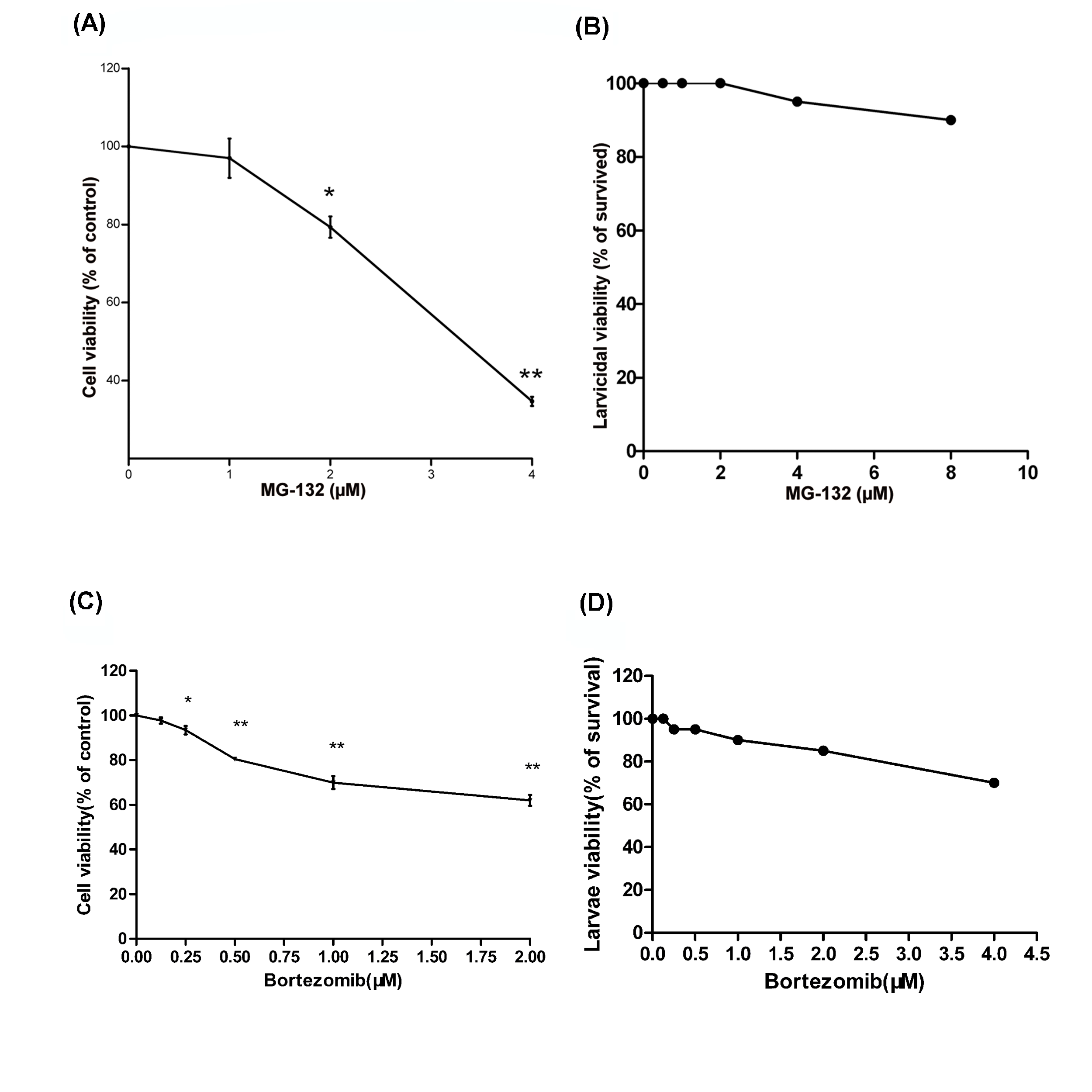

Supplement: Figure S2 — Effect of proteasome inhibitors on cell or larvae viability. DM-resistant mosquito cells were treated with MG-132 (A) or bortezomib (B) at the indicated concentrations for 72 h and the cell viability was measured by CCK-8 assay. Results are expressed as the mean±SEM. *P<0.05, **P<0.01 compared with the DMSO control. Larvae of the early fourth instar were exposed to MG-132 (C) or bortezomib (D) at the indicated concentrations for 24 h before the survival was calculated. The results shown are representative of three independent experiments. (TIF) [file pone.0065859.s002.tif]

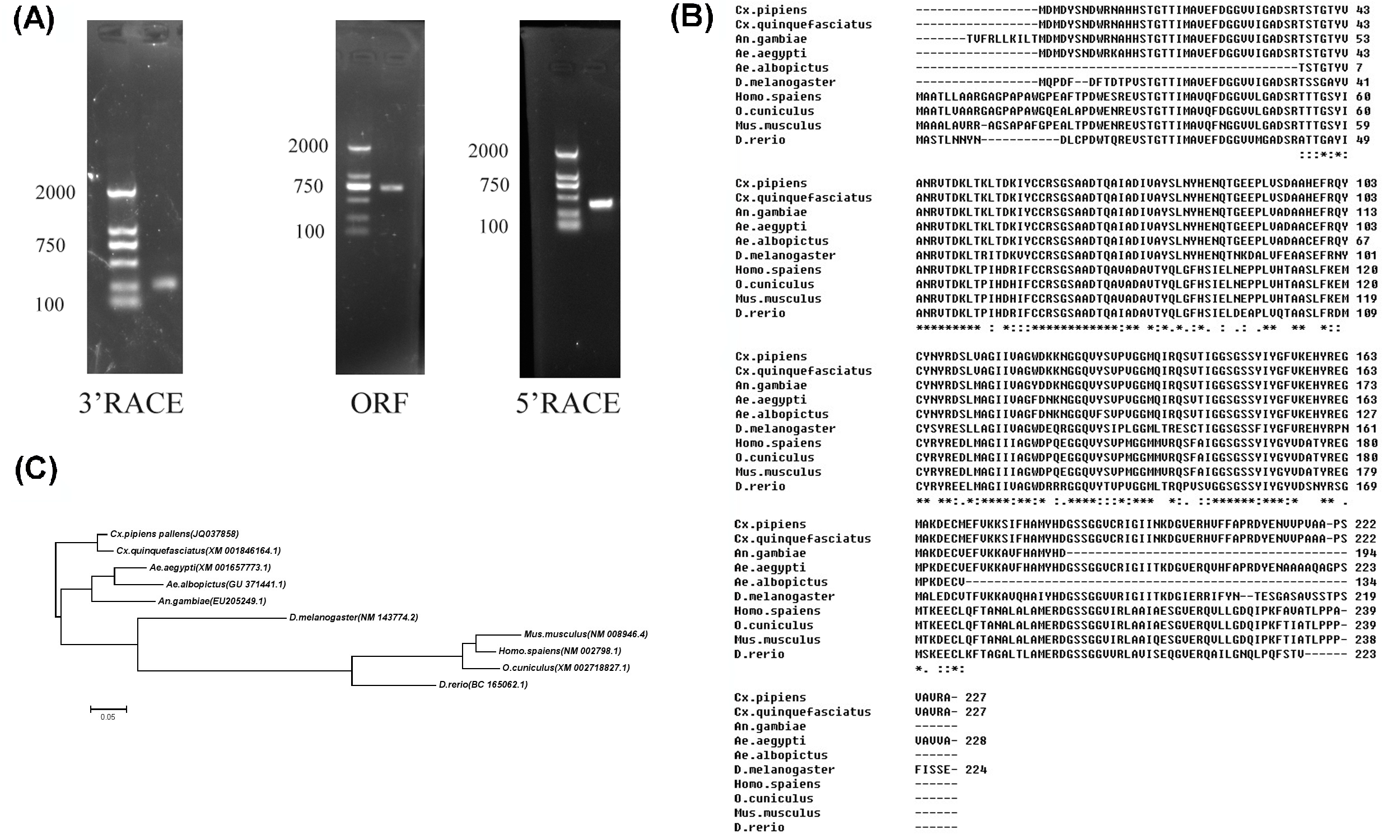

Supplement: Figure S3 — Molecular cloning and sequence analysis of PSMB6 . (A).Three fragments of the cDNA sequence of PSMB6. The PCR product was cloned from Cx. pipiens pallen and separated by electrophoresis. (B) Homology analysis of PSMB6 cloned from Cx. pipiens pallen. (C) Phylogenetic relationship of PSMB6 with other species. (TIF) [file pone.0065859.s003.tif]
