# Supplementary material for: Identification of Proteasome Subunit Beta Type 6 (PSMB6) Associated with Deltamethrin Resistance in Mosquitoes by Proteomic and Bioassay Analyses
Source: PLoS One. 2013 Jun 10;8(6):e65859. doi: 10.1371/journal.pone.0065859 (PMC3677870; doi:10.1371/journal.pone.0065859)
Supplement: Table S1 — Sequences of primers used for quantitative PCR. (DOC) [file pone.0065859.s004.doc]

**Table S1. Primer sequences of genes used in Quantitative Real-Time PCR.**

| Gene name | Primers | Primer sequences (5'- 3') |
| --- | --- | --- |
| *Aedes Albopticus* | | |
| β-Actin (XM_001844545.1) | forward | CCACCATGTACCCAGGAATC |
|  | reverse | CACCGATCCAGACGGAGTAT |
| PSMB6(GU371441.1) | forward | GAGTTCCGCCAGTATTGCTACA |
|  | reverse | CCTGCCCTCCGTTCTTGTTA |
| *Culex pipiens pallens* | | |
| β-Actin (AY_100005.1) | forward | AGCGTGAACTGACGGCTCTTG |
|  | reverse | ACTCGTCGTACTCCTGCTTGG |
| PSMB6(JQ037858) | forward | GCTGGTGGCGGGTATCATT |
|  | reverse | ATGGTGACACTCTGCCGAATC |
| POH1（XM_001849534.1） | forward | GACTGATGCTGGGAGAGTTTG |
|  | reverse | CGACTGCTGCGTGTTGATG |
| USP（XM_001846893） | forward | AGAAAGAGCGAGAGCGGGT |
|  | reverse | TGGTGTAGGTTGTAGGAGGTGG |
| E2（XM_001864564.1） | forward | CCAATGTTTGCGTTGTCGTC |
|  | reverse | TGTGGTGTGATAGTAAGTTCCTGTC |
