# Supplementary material for: Identification of Proteasome Subunit Beta Type 6 (PSMB6) Associated with Deltamethrin Resistance in Mosquitoes by Proteomic and Bioassay Analyses
Source: PLoS One. 2013 Jun 10;8(6):e65859. doi: 10.1371/journal.pone.0065859 (PMC3677870; doi:10.1371/journal.pone.0065859)
Supplement: Table S2 — The MALDI-TOF-MS raw data for all peptides (data used to generate Table 1 ). (DOC) [file pone.0065859.s005.doc]

| **Table S2**. The raw data with all peptides(only those used for generating Table 1) by MALDI-TOF-MS | | | | | | | | | |
| --- | --- | --- | --- | --- | --- | --- | --- | --- | --- |
| ID | m/z | Intens. | SN | Quality Fac. | Res. | Area | Rel. Intens. | FWHM | Chi^2 |
| 253 | 774.4172 | 81.97255 | 17.97168 | 490.6264 | 8037.806 | 13.21653 | 9.10E-03 | 9.63E-02 | 43.7438 |
|  | 782.4543 | 36.51272 | 7.964874 | 110.7972 | 14845.05 | 3.405422 | 4.05E-03 | 5.27E-02 | 28.02138 |
|  | 788.464 | 116.7797 | 26.53704 | 1273.849 | 7954.689 | 19.23091 | 1.30E-02 | 9.91E-02 | 31.82402 |
|  | 804.2507 | 74.2196 | 16.80672 | 532.242 | 9592.852 | 12.1992 | 8.24E-03 | 8.38E-02 | 33.51087 |
|  | 838.4566 | 66.39934 | 14.78153 | 442.4623 | 7928.29 | 12.35988 | 7.37E-03 | 0.105755 | 34.98229 |
|  | 856.5053 | 22.76301 | 5.22401 | 107.9991 | 7408.664 | 4.423876 | 2.53E-03 | 0.115609 | 9.631264 |
|  | 870.5421 | 151.7522 | 36.06254 | 492.9298 | 8893.123 | 25.43902 | 1.68E-02 | 9.79E-02 | 163.3585 |
|  | 942.5477 | 356.5703 | 91.73609 | 692.4342 | 9853.459 | 61.80801 | 3.96E-02 | 0.095657 | 806.3313 |
|  | 950.5261 | 35.48633 | 9.002085 | 262.076 | 8803.267 | 6.949394 | 3.94E-03 | 0.107974 | 16.24053 |
|  | 1028.621 | 20.82255 | 4.812556 | 62.58998 | 9516.175 | 4.146943 | 2.31E-03 | 0.108092 | 17.83221 |
|  | 1032.612 | 158.7906 | 36.92512 | 1012.41 | 8815.318 | 36.24187 | 1.76E-02 | 0.117138 | 103.8763 |
|  | 1049.529 | 1135.396 | 275.0893 | 4212.273 | 9841.395 | 233.8046 | 0.125995 | 0.106644 | 5051.453 |
|  | 1161.676 | 86.02604 | 23.01608 | 874.2743 | 9309.265 | 22.31967 | 9.55E-03 | 0.124787 | 62.24579 |
|  | 1177.628 | 66.50256 | 17.82357 | 414.6181 | 11365.05 | 15.80513 | 7.38E-03 | 0.103618 | 50.79375 |
|  | 1190.642 | 23.49407 | 6.612659 | 88.99636 | 14697.47 | 5.06119 | 2.61E-03 | 8.10E-02 | 23.82347 |
|  | 1218.711 | 31.4181 | 9.314988 | 59.21373 | 11372.83 | 8.305365 | 3.49E-03 | 0.10716 | 34.44155 |
|  | 1277.726 | 25.38091 | 7.008564 | 108.0834 | 21764.42 | 3.82481 | 2.82E-03 | 5.87E-02 | 15.01936 |
|  | 1327.739 | 84.72279 | 24.42879 | 866.6631 | 11213.44 | 22.98826 | 9.40E-03 | 0.118406 | 42.00765 |
|  | 1335.706 | 78.59 | 22.0269 | 1212.543 | 12911.75 | 20.54922 | 8.72E-03 | 0.103449 | 24.87331 |
|  | 1349.715 | 15.95091 | 4.418291 | 174.0858 | 10255.82 | 5.179919 | 1.77E-03 | 0.131605 | 5.082748 |
|  | 1405.758 | 55.79543 | 14.62237 | 652.4248 | 10610.61 | 17.51801 | 6.19E-03 | 0.132486 | 29.9178 |
|  | 1457.73 | 1909.688 | 491.6181 | 19382.3 | 10117.54 | 650.8078 | 0.211918 | 0.144079 | 10099.36 |
|  | 1474.772 | 18.31289 | 5.092146 | 59.07062 | 8699.333 | 6.346106 | 2.03E-03 | 0.169527 | 16.26262 |
|  | 1703.903 | 15.67824 | 4.568811 | 74.51507 | 12958.41 | 5.674208 | 1.74E-03 | 0.13149 | 7.008315 |
|  | 1719.905 | 2882.422 | 818.1189 | 26457.37 | 11088.17 | 1255.431 | 0.319862 | 0.155112 | 20547.81 |
|  | 1741.882 | 60.65959 | 16.01227 | 923.5301 | 10058.84 | 30.81606 | 6.73E-03 | 0.173169 | 35.45781 |
|  | 1790.998 | 9011.464 | 2176.212 | 48904 | 10765.19 | 4597.03 | 1 | 0.166369 | 76080.58 |
|  | 1812.98 | 12.57415 | 3.51015 | 77.45323 | 11526.82 | 5.503353 | 1.40E-03 | 0.157284 | 11.31985 |
|  | 1848.015 | 20.97085 | 8.103196 | 442.2892 | 10916.93 | 10.56377 | 2.33E-03 | 0.16928 | 8.58145 |
|  | 1852.918 | 15.53287 | 6.278265 | 304.8508 | 11653.6 | 9.258725 | 1.72E-03 | 0.159 | 8.544262 |
|  | 1910.996 | 19.4152 | 6.893348 | 250.1768 | 14158.47 | 7.476007 | 2.15E-03 | 0.134972 | 12.274 |
|  | 1919.084 | 379.8724 | 126.6269 | 4704.089 | 14386.01 | 156.163 | 4.22E-02 | 0.133399 | 1284.776 |
|  | 1947.009 | 16.65887 | 4.559672 | 92.26118 | 10637.2 | 9.838255 | 1.85E-03 | 0.183038 | 17.40964 |
|  | 1964.978 | 6223.899 | 1578.287 | 15532.88 | 11559.65 | 3389.106 | 0.690665 | 0.169986 | 118099.6 |
|  | 2023.966 | 105.1102 | 48.23244 | 864.9197 | 16865.46 | 39.70543 | 1.17E-02 | 0.120007 | 116.947 |
|  | 2136.029 | 17.99682 | 13.51067 | 249.0022 | 14344.7 | 9.419763 | 2.00E-03 | 0.148907 | 13.75418 |
|  | 2152.041 | 601.7536 | 454.6561 | 11882.44 | 15163.02 | 278.7345 | 6.68E-02 | 0.141927 | 2675.659 |
|  | 2174.047 | 10.19134 | 8.649229 | 263.0215 | 11632.65 | 6.827186 | 1.13E-03 | 0.186892 | 4.959718 |
|  | 2303.195 | 10.28175 | 16.33254 | 256.2592 | 16163.88 | 5.506602 | 1.14E-03 | 0.14249 | 5.010333 |
|  | 2362.202 | 15.49637 | 31.41137 | 1088.76 | 20883.57 | 6.527899 | 1.72E-03 | 0.113113 | 5.995532 |
|  | 2462.307 | 7.757031 | 27.88976 | 241.5297 | 20114.55 | 3.145702 | 8.61E-04 | 0.122414 | 2.99248 |
|  |  |  |  |  |  |  |  |  |  |
| ID | m/z | Intens. | SN | Quality Fac. | Res. | Area | Rel. Intens. | FWHM | Chi^2 |
| 407 | 771.3093 | 24.20236 | 7.080701 | 97.30966 | 12232.7 | 4.199866 | 1.44E-03 | 6.31E-02 | 19.04533 |
|  | 804.2422 | 33.08668 | 7.649983 | 263.6585 | 8023.651 | 6.085413 | 1.97E-03 | 0.100234 | 11.69556 |
|  | 837.4343 | 177.9818 | 30.0356 | 504.0292 | 10742.07 | 24.45461 | 1.06E-02 | 7.80E-02 | 179.2592 |
|  | 854.4666 | 12398.24 | 2389.98 | 19332.09 | 7424.786 | 2476.506 | 0.736572 | 0.115083 | 119769.5 |
|  | 870.5397 | 350.9637 | 79.19462 | 925.187 | 9483.951 | 54.29665 | 2.09E-02 | 9.18E-02 | 426.9563 |
|  | 1049.425 | 178.3275 | 55.59386 | 777.4665 | 9648.782 | 36.49123 | 1.06E-02 | 0.108762 | 176.3884 |
|  | 1066.463 | 2511.995 | 759.944 | 13606.9 | 9024.889 | 588.1034 | 0.149236 | 0.118169 | 11059.97 |
|  | 1069.545 | 384.9161 | 116.8586 | 1222.765 | 8693.952 | 82.85592 | 2.29E-02 | 0.123022 | 1286.898 |
|  | 1193.614 | 20.80091 | 5.190859 | 55.58785 | 8300.873 | 6.678218 | 1.24E-03 | 0.143794 | 18.00004 |
|  | 1200.72 | 463.4511 | 108.372 | 2786.318 | 10264.93 | 113.6045 | 2.75E-02 | 0.116973 | 1022.304 |
|  | 1228.619 | 6479.396 | 1349.336 | 35471.52 | 7794.445 | 2121.412 | 0.384937 | 0.157627 | 49592.81 |
|  | 1240.618 | 64.73594 | 13.59056 | 486.1149 | 10034.48 | 17.41805 | 3.85E-03 | 0.123635 | 40.06979 |
|  | 1277.723 | 37.92662 | 9.930409 | 653.0128 | 10708.41 | 10.47807 | 2.25E-03 | 0.11932 | 10.60255 |
|  | 1302.722 | 15.98821 | 4.992127 | 109.0879 | 10084.45 | 5.591231 | 9.50E-04 | 0.129181 | 10.38827 |
|  | 1327.744 | 19.62344 | 6.097491 | 94.16606 | 12356.28 | 5.242088 | 1.17E-03 | 0.107455 | 14.46111 |
|  | 1348.634 | 149.3606 | 45.04239 | 2401.563 | 11727.96 | 42.63881 | 8.87E-03 | 0.114993 | 54.33713 |
|  | 1410.761 | 33.398 | 9.858411 | 374.9986 | 10307.63 | 11.41211 | 1.98E-03 | 0.136866 | 18.97354 |
|  | 1502.819 | 35.5263 | 9.789078 | 462.9107 | 10120.1 | 14.01386 | 2.11E-03 | 0.148498 | 18.1295 |
|  | 1531.805 | 58.1705 | 15.59545 | 593.2025 | 8913.742 | 26.63235 | 3.46E-03 | 0.171848 | 53.75484 |
|  | 1550.805 | 71.15349 | 13.16681 | 1323.347 | 12616.5 | 23.84975 | 4.23E-03 | 0.122919 | 22.82118 |
|  | 1566.792 | 42.35928 | 5.95923 | 160.5953 | 17633.89 | 14.34456 | 2.52E-03 | 8.89E-02 | 74.86329 |
|  | 1567.773 | 84.67042 | 11.49766 | 535.0239 | 11181.39 | 30.87981 | 5.03E-03 | 0.140213 | 87.69476 |
|  | 1597.873 | 280.5259 | 26.38178 | 708.2708 | 10143.49 | 105.1345 | 1.67E-02 | 0.157527 | 709.2271 |
|  | 1598.871 | 182.3133 | 17.10785 | 328.8732 | 11764.44 | 66.16766 | 1.08E-02 | 0.135907 | 675.3062 |
|  | 1605.538 | 1147.994 | 100.4252 | 797.9777 | 10036.92 | 543.3495 | 0.068202 | 0.159963 | 21107.32 |
|  | 1606.528 | 525.2612 | 45.22416 | 217.8496 | 10800.11 | 348.1403 | 3.12E-02 | 0.148751 | 13808.48 |
|  | 1645.87 | 16832.35 | 1687.774 | 3163.859 | 9691.202 | 8194.785 | 1 | 0.169831 | 3227667 |
|  | 1648.86 | 930.7324 | 96.49387 | 62.21185 | 10613.21 | 393.3359 | 5.53E-02 | 0.155359 | 110198.4 |
|  | 1661.873 | 3287.577 | 392.1864 | 6270.339 | 11438.18 | 1401.455 | 0.195313 | 0.145292 | 77589.2 |
|  | 1662.851 | 1966.446 | 238.6354 | 690.8596 | 10481.05 | 933.266 | 0.116825 | 0.158653 | 57138.11 |
|  | 1695.83 | 161.6116 | 32.18729 | 1248.529 | 14248.78 | 55.55771 | 9.60E-03 | 0.119016 | 156.1827 |
|  | 1739.835 | 27.33836 | 7.03012 | 784.3528 | 14105.77 | 10.54361 | 1.62E-03 | 0.123342 | 6.37076 |
|  | 1799.962 | 22.92006 | 6.022384 | 52.9361 | 13920.12 | 9.652696 | 1.36E-03 | 0.129306 | 63.21736 |
|  | 1802.827 | 134.5364 | 34.71913 | 1998.04 | 10776.4 | 71.12566 | 7.99E-03 | 0.167294 | 85.41098 |
|  | 1810.401 | 219.5786 | 52.7824 | 1923.181 | 10898.26 | 115.2631 | 1.30E-02 | 0.166118 | 354.306 |
|  | 1850.825 | 8264.769 | 1441.296 | 44208.48 | 10537.77 | 4570.336 | 0.491005 | 0.175637 | 56740.89 |
|  | 1855.013 | 255.0539 | 43.52789 | 685.3009 | 9703.455 | 90.15773 | 1.52E-02 | 0.19117 | 1403.903 |
|  | 1866.82 | 1168.3 | 184.7693 | 12611.55 | 13497.47 | 531.6335 | 6.94E-02 | 0.138309 | 6913.842 |
|  | 1937.968 | 43.48427 | 14.05094 | 507.0893 | 14875.53 | 16.7416 | 2.58E-03 | 0.130279 | 31.62098 |
|  | 1953.974 | 91.1194 | 40.51154 | 621.1151 | 14689.35 | 36.77118 | 5.41E-03 | 0.13302 | 124.3138 |
|  | 1978.918 | 173.2568 | 99.06241 | 1015.378 | 15301.48 | 73.11317 | 1.03E-02 | 0.129329 | 265.8323 |
|  | 2014.053 | 250.3719 | 162.5573 | 7573.51 | 15692.25 | 103.1072 | 1.49E-02 | 0.128347 | 354.1501 |
|  | 2036.156 | 19.77688 | 14.14724 | 235.4199 | 14893.86 | 9.41021 | 1.17E-03 | 0.136711 | 17.67741 |
|  | 2418.171 | 38.55266 | 74.33829 | 1705.603 | 19971.43 | 16.99857 | 2.29E-03 | 0.121081 | 28.47167 |
|  | 2435.2 | 190.4877 | 339.736 | 1945.055 | 18431.64 | 90.13711 | 1.13E-02 | 0.132121 | 1212.669 |
|  | 2536.273 | 47.60663 | 25.16397 | 418.5117 | 21179.33 | 25.18612 | 2.83E-03 | 0.119752 | 71.45989 |
|  | 2553.293 | 4931.586 | 2728.667 | 6565.618 | 14178.21 | 3141.694 | 0.292983 | 0.180086 | 128996.7 |
|  | 2650.205 | 90.88294 | 205.4458 | 1129.128 | 20479.29 | 39.57251 | 5.40E-03 | 0.129409 | 312.5982 |
|  |  |  |  |  |  |  |  |  |  |
| ID | m/z | Intens. | SN | Quality Fac. | Res. | Area | Rel. Intens. | FWHM | Chi^2 |
| 508 | 703.206 | 36.14312 | 4.817163 | 62.66566 | 5073.383 | 8.022955 | 5.25E-03 | 0.138607 | 39.01649 |
|  | 720.6098 | 45.43965 | 6.014158 | 92.76194 | 9727.896 | 5.180204 | 6.61E-03 | 7.41E-02 | 39.93791 |
|  | 738.3622 | 32.98667 | 4.364094 | 89.16659 | 6383.296 | 7.318708 | 4.80E-03 | 0.115671 | 24.93098 |
|  | 765.5727 | 387.3691 | 50.58629 | 109.1748 | 8300.005 | 57.54246 | 5.63E-02 | 9.22E-02 | 3994.335 |
|  | 767.5671 | 355.4463 | 45.71968 | 204.4662 | 8145.111 | 51.98652 | 5.17E-02 | 9.42E-02 | 2015.81 |
|  | 804.2737 | 34.81773 | 4.426259 | 82.16161 | 6914.41 | 8.80667 | 5.06E-03 | 0.116318 | 32.65669 |
|  | 810.3202 | 161.1149 | 19.60875 | 53.72828 | 11694.86 | 19.73383 | 2.34E-02 | 6.93E-02 | 1317.104 |
|  | 812.3256 | 208.9832 | 26.41681 | 121.064 | 9343.725 | 30.11488 | 3.04E-02 | 0.086938 | 1085.453 |
|  | 814.3292 | 94.37106 | 11.8754 | 85.35441 | 12996.93 | 10.16878 | 1.37E-02 | 6.27E-02 | 292.6891 |
|  | 819.4098 | 31.04021 | 3.906091 | 86.00083 | 4909.367 | 10.13892 | 4.51E-03 | 0.166907 | 30.16021 |
|  | 820.41 | 45.80548 | 5.7546 | 207.3873 | 9491.617 | 7.015888 | 6.66E-03 | 8.64E-02 | 26.31886 |
|  | 837.4861 | 950.9671 | 118.9372 | 5181.861 | 6355.597 | 208.9549 | 0.138237 | 0.131771 | 1335.912 |
|  | 856.5059 | 182.6915 | 22.75959 | 663.6449 | 5237.889 | 49.73754 | 2.66E-02 | 0.163521 | 205.0141 |
|  | 870.5442 | 663.0344 | 81.83939 | 2406.51 | 7727.252 | 141.4341 | 9.64E-02 | 0.112659 | 1509.404 |
|  | 904.4376 | 599.5269 | 77.652 | 6380.732 | 6904.981 | 148.4295 | 0.08715 | 0.130983 | 1114.838 |
|  | 921.4632 | 488.8963 | 66.7856 | 3002.853 | 7926.999 | 111.5615 | 7.11E-02 | 0.116244 | 1147.313 |
|  | 937.4697 | 73.69114 | 10.58817 | 415.5007 | 10189.66 | 14.10845 | 1.07E-02 | 9.20E-02 | 45.34891 |
|  | 945.552 | 128.1297 | 19.17827 | 892.6581 | 6685.105 | 32.90087 | 1.86E-02 | 0.141442 | 68.78423 |
|  | 950.5115 | 28.6375 | 4.372573 | 109.2087 | 6639.525 | 8.748512 | 4.16E-03 | 0.14316 | 20.68004 |
|  | 961.5543 | 50.89754 | 8.210547 | 136.3845 | 10676.41 | 10.18645 | 7.40E-03 | 9.01E-02 | 70.75184 |
|  | 1046.131 | 178.6546 | 31.3045 | 61.31231 | 14115.1 | 26.45153 | 2.60E-02 | 7.41E-02 | 1626.614 |
|  | 1048.124 | 100.3907 | 17.99519 | 82.46027 | 12081.63 | 17.94284 | 1.46E-02 | 8.68E-02 | 487.3624 |
|  | 1096.6 | 31.9318 | 5.251008 | 130.1534 | 7247.274 | 11.28997 | 4.64E-03 | 0.151312 | 37.0551 |
|  | 1114.654 | 6879.248 | 1106.679 | 34142.73 | 7479.357 | 2178.989 | 1 | 0.149031 | 62014.48 |
|  | 1130.618 | 34.10615 | 5.708032 | 158.1213 | 8534.384 | 9.699116 | 4.96E-03 | 0.132478 | 23.89587 |
|  | 1180.615 | 33.56778 | 6.304918 | 212.2461 | 9416.49 | 8.273908 | 4.88E-03 | 0.125377 | 15.50955 |
|  | 1207.66 | 26.53949 | 5.018815 | 95.82057 | 10964.92 | 6.817579 | 3.86E-03 | 0.110138 | 18.60664 |
|  | 1217.69 | 33.04684 | 6.210567 | 98.33053 | 7835.317 | 11.41678 | 4.80E-03 | 0.15541 | 31.57053 |
|  | 1247.586 | 151.861 | 27.19988 | 979.5992 | 8362.789 | 48.21863 | 2.21E-02 | 0.149183 | 180.0783 |
|  | 1258.662 | 24.73579 | 4.1195 | 133.1426 | 10046.02 | 9.550453 | 3.60E-03 | 0.12529 | 17.15306 |
|  | 1285.75 | 738.612 | 105.1019 | 4940.287 | 9544.772 | 231.2308 | 0.107368 | 0.134707 | 1646.492 |
|  | 1294.624 | 168.6857 | 22.9662 | 1695.998 | 7574.336 | 62.08323 | 2.45E-02 | 0.170923 | 100.9621 |
|  | 1306.639 | 87.41544 | 11.28362 | 572.7925 | 8976.073 | 30.02035 | 1.27E-02 | 0.145569 | 71.83893 |
|  | 1315.716 | 2389.458 | 322.3228 | 24953.33 | 10130.84 | 740.3141 | 0.347343 | 0.129872 | 9156.24 |
|  | 1327.735 | 30.55044 | 4.277375 | 134.8233 | 10895.76 | 9.58815 | 4.44E-03 | 0.121858 | 21.90372 |
|  | 1337.715 | 75.83296 | 10.91933 | 417.5011 | 7984.756 | 27.33866 | 1.10E-02 | 0.167534 | 71.93353 |
|  | 1397.73 | 248.6302 | 35.90431 | 4958.075 | 8792.986 | 98.45984 | 3.61E-02 | 0.15896 | 159.3806 |
|  | 1405.46 | 65.43462 | 9.150911 | 539.2736 | 9499.421 | 28.64322 | 9.51E-03 | 0.147952 | 51.74764 |
|  | 1416.771 | 82.09565 | 10.95636 | 280.3499 | 15758.14 | 22.63359 | 1.19E-02 | 8.99E-02 | 129.7138 |
|  | 1427.735 | 225.6635 | 28.56172 | 5350.915 | 10414.01 | 77.87217 | 3.28E-02 | 0.137098 | 110.2061 |
|  | 1435.439 | 49.98764 | 6.136174 | 316.2576 | 11311.06 | 19.14562 | 7.27E-03 | 0.126906 | 47.97035 |
|  | 1445.723 | 2185.752 | 272.9152 | 36517.6 | 9223.445 | 819.664 | 0.317731 | 0.156744 | 6414.594 |
|  | 1461.734 | 204.4751 | 27.41611 | 975.2582 | 11395.25 | 68.46324 | 0.029723 | 0.128276 | 258.8475 |
|  | 1477.725 | 172.8384 | 25.62863 | 51.26524 | 13766.5 | 26.78155 | 2.51E-02 | 0.107342 | 2372.035 |
|  | 1491.738 | 202.6544 | 32.65552 | 975.6255 | 11615 | 64.72644 | 2.95E-02 | 0.128432 | 306.1847 |
|  | 1507.731 | 71.56302 | 12.75608 | 705.2722 | 13235.76 | 23.83477 | 1.04E-02 | 0.113913 | 42.16647 |
|  | 1550.79 | 33.99491 | 6.326236 | 117.0578 | 8550.541 | 13.6876 | 4.94E-03 | 0.181367 | 72.82588 |
|  | 1552.805 | 34.57446 | 6.409869 | 155.5 | 8092.933 | 20.28948 | 5.03E-03 | 0.191872 | 70.90293 |
|  | 1568.812 | 5598.187 | 1035.297 | 43434.38 | 9127.193 | 2521.493 | 0.813779 | 0.171883 | 40348.7 |
|  | 1575.81 | 21.94223 | 4.145801 | 134.6477 | 8384.985 | 7.738536 | 3.19E-03 | 0.187932 | 21.11195 |
|  | 1673.818 | 17.11324 | 5.302616 | 65.07134 | 8431.631 | 10.0456 | 2.49E-03 | 0.198517 | 10.82475 |
|  | 1689.82 | 161.6821 | 51.19587 | 3473.336 | 11394.54 | 68.29994 | 2.35E-02 | 0.148301 | 268.0932 |
|  | 1708.991 | 13.13768 | 4.287058 | 133.7897 | 8028.071 | 6.990264 | 1.91E-03 | 0.212877 | 7.416255 |
|  | 1736.924 | 56.02157 | 19.13185 | 1133.8 | 13359.02 | 24.70001 | 8.14E-03 | 0.130019 | 22.87145 |
|  | 1756.99 | 195.1069 | 72.12233 | 5548.955 | 14272.95 | 79.96086 | 2.84E-02 | 0.123099 | 300.9213 |
|  | 1822.038 | 14.78827 | 7.207298 | 180.4039 | 14637.56 | 7.398059 | 2.15E-03 | 0.124477 | 8.531023 |
|  | 1891.925 | 137.9281 | 77.87742 | 5775.93 | 13098.8 | 65.04073 | 2.00E-02 | 0.144435 | 169.8488 |
|  | 2059.03 | 24.23552 | 15.80731 | 582.631 | 12851.14 | 12.61412 | 3.52E-03 | 0.160222 | 9.153302 |
|  | 2116.079 | 450.102 | 218.9108 | 3736.401 | 13664.31 | 240.6398 | 6.54E-02 | 0.154862 | 3403.8 |
|  | 2117.046 | 191.6985 | 94.38683 | 191.3502 | 13215.58 | 97.42514 | 2.79E-02 | 0.160193 | 2413.532 |
|  | 2145.062 | 24.28736 | 10.43269 | 260.8899 | 17970.79 | 13.31414 | 3.53E-03 | 0.119364 | 26.84849 |
|  | 2159.207 | 9.285205 | 3.555255 | 74.67933 | 15536.53 | 4.411978 | 1.35E-03 | 0.138976 | 4.08487 |
|  | 2222.131 | 2761.856 | 826.219 | 12959.26 | 12018.77 | 1648.716 | 0.401476 | 0.184888 | 29448.42 |
|  | 2348.159 | 9.58327 | 4.676318 | 155.1898 | 5786.586 | 14.54734 | 1.39E-03 | 0.405794 | 11.59272 |
|  | 2371.245 | 94.72739 | 39.61192 | 722.2762 | 7751.963 | 87.02207 | 1.38E-02 | 0.30589 | 227.2147 |
|  | 2387.276 | 1330.455 | 514.7434 | 7524.028 | 13445.8 | 820.8703 | 0.193401 | 0.177548 | 13463.96 |
|  |  |  |  |  |  |  |  |  |  |
| ID | m/z | Intens. | SN | Quality Fac. | Res. | Area | Rel. Intens. | FWHM | Chi^2 |
| 536 | 747.4369 | 1265.028 | 90.60238 | 1237.106 | 6103.691 | 275.9025 | 9.45E-02 | 0.122457 | 5038.364 |
|  | 751.3794 | 146.2654 | 10.39185 | 628.0838 | 5081.881 | 36.1876 | 1.09E-02 | 0.147855 | 113.2926 |
|  | 756.3854 | 69.24517 | 4.875559 | 126.3975 | 5830.657 | 17.0488 | 5.17E-03 | 0.129726 | 86.38854 |
|  | 769.3453 | 69.72693 | 4.8937 | 125.5201 | 5302.481 | 20.46665 | 5.21E-03 | 0.145092 | 91.3906 |
|  | 774.4398 | 436.109 | 30.49079 | 2714.13 | 5998.543 | 100.9186 | 3.26E-02 | 0.129105 | 234.4032 |
|  | 802.4432 | 103.5499 | 7.098942 | 265.149 | 5984.842 | 23.1624 | 7.73E-03 | 0.134079 | 138.0471 |
|  | 804.261 | 417.3402 | 28.35209 | 1573.739 | 5452.711 | 107.5089 | 3.12E-02 | 0.147497 | 415.0764 |
|  | 829.2146 | 561.8462 | 37.86377 | 635.9644 | 6009.387 | 141.2046 | 4.20E-02 | 0.137987 | 1876.022 |
|  | 838.4678 | 120.9182 | 8.269515 | 290.9305 | 5671.166 | 28.91933 | 9.03E-03 | 0.147848 | 181.3666 |
|  | 842.5094 | 1857.36 | 129.5454 | 2492.25 | 5922.068 | 460.5354 | 0.138703 | 0.142266 | 7890.902 |
|  | 856.5226 | 240.7617 | 17.374 | 976.3354 | 6600.111 | 53.48968 | 1.80E-02 | 0.129774 | 202.359 |
|  | 870.5464 | 897.7147 | 67.21046 | 1340.908 | 6680.1 | 221.3988 | 6.70E-02 | 0.130319 | 2298.596 |
|  | 908.446 | 58.29442 | 4.566149 | 108.4469 | 8376.902 | 11.46083 | 4.35E-03 | 0.108447 | 75.34664 |
|  | 914.5061 | 535.0463 | 41.9432 | 1674.078 | 7314.305 | 123.0286 | 4.00E-02 | 0.12503 | 626.0522 |
|  | 930.5253 | 367.3972 | 28.66933 | 968.9911 | 7344.961 | 83.92815 | 2.74E-02 | 0.126689 | 565.9389 |
|  | 941.4903 | 68.1402 | 5.385972 | 76.72599 | 6417.247 | 15.75774 | 5.09E-03 | 0.146712 | 164.9094 |
|  | 950.5195 | 73.56865 | 6.064224 | 174.522 | 8046.571 | 16.41023 | 5.49E-03 | 0.118127 | 106.6798 |
|  | 1045.556 | 253.8613 | 22.50393 | 579.2234 | 6493.512 | 79.82793 | 1.90E-02 | 0.161016 | 614.7854 |
|  | 1061.497 | 431.1628 | 37.41122 | 3527.944 | 6366.868 | 142.09 | 3.22E-02 | 0.166722 | 410.9932 |
|  | 1064.537 | 167.8597 | 14.67346 | 760.3782 | 8799.14 | 48.99885 | 1.25E-02 | 0.120982 | 171.6216 |
|  | 1118.579 | 113.2492 | 9.301329 | 747.396 | 6547.548 | 40.39045 | 8.46E-03 | 0.170839 | 81.88234 |
|  | 1128.587 | 80.98972 | 6.625011 | 123.7167 | 6215.05 | 31.02214 | 6.05E-03 | 0.181589 | 205.8039 |
|  | 1132.398 | 54.67579 | 4.484064 | 133.1909 | 5933.5 | 43.96712 | 4.08E-03 | 0.190848 | 126.6344 |
|  | 1152.601 | 52.93882 | 4.309211 | 95.9858 | 6900.205 | 19.22204 | 3.95E-03 | 0.167039 | 85.32684 |
|  | 1165.603 | 63.77053 | 5.115813 | 147.995 | 8536.71 | 18.87237 | 4.76E-03 | 0.13654 | 78.28856 |
|  | 1189.57 | 242.2216 | 17.43883 | 1811.35 | 7873.399 | 77.94697 | 1.81E-02 | 0.151087 | 154.3449 |
|  | 1191.61 | 53.35353 | 3.848839 | 114.8122 | 6786.139 | 20.76493 | 3.98E-03 | 0.175595 | 131.1835 |
|  | 1193.619 | 86.47087 | 6.074708 | 161.3961 | 8064.758 | 24.6124 | 6.46E-03 | 0.148004 | 150.5139 |
|  | 1198.633 | 58.67725 | 4.055856 | 128.4191 | 6840.192 | 22.30128 | 4.38E-03 | 0.175234 | 78.46522 |
|  | 1209.596 | 13390.92 | 871.5995 | 328220.5 | 5924.115 | 6107.377 | 1 | 0.204182 | 23379.28 |
|  | 1223.602 | 113.805 | 6.886395 | 202.4467 | 7841.154 | 40.02659 | 8.50E-03 | 0.156049 | 304.3543 |
|  | 1237.644 | 250.0142 | 14.01523 | 1010.713 | 7990.968 | 83.89978 | 1.87E-02 | 0.15488 | 301.9008 |
|  | 1242.743 | 1716.374 | 98.23447 | 6036.029 | 8066.67 | 605.51 | 0.128175 | 0.154059 | 8250.798 |
|  | 1264.678 | 73.5421 | 4.542603 | 212.2448 | 8377.173 | 24.81158 | 5.49E-03 | 0.150967 | 86.55768 |
|  | 1275.671 | 285.9123 | 18.40257 | 1604.366 | 9889.898 | 95.3958 | 0.021351 | 0.128987 | 300.5296 |
|  | 1309.639 | 260.8553 | 18.88438 | 1799.53 | 7293.017 | 99.06211 | 1.95E-02 | 0.179574 | 221.8843 |
|  | 1317.666 | 73.78816 | 5.304478 | 124.202 | 11068.23 | 23.33463 | 5.51E-03 | 0.119049 | 149.8322 |
|  | 1320.587 | 169.758 | 12.38962 | 785.5107 | 8867.427 | 67.68448 | 1.27E-02 | 0.148926 | 194.3244 |
|  | 1327.727 | 162.8307 | 11.88107 | 357.332 | 6951.802 | 64.8362 | 1.22E-02 | 0.19099 | 442.2933 |
|  | 1329.646 | 361.9203 | 26.76824 | 1394.55 | 7319.62 | 149.6385 | 2.70E-02 | 0.181655 | 544.3614 |
|  | 1333.709 | 935.9825 | 69.38109 | 19514.82 | 7299.936 | 403.6607 | 6.99E-02 | 0.182701 | 936.206 |
|  | 1341.625 | 82.40282 | 6.169225 | 503.6895 | 3967.239 | 63.31534 | 6.15E-03 | 0.338176 | 83.53155 |
|  | 1381.705 | 2045.083 | 159.3219 | 70325.96 | 7542.618 | 894.2503 | 0.152722 | 0.183186 | 2421.031 |
|  | 1397.713 | 258.5711 | 20.86284 | 1339.666 | 8636.393 | 103.0907 | 0.019309 | 0.16184 | 300.5622 |
|  | 1435.721 | 111.7172 | 9.69853 | 400.9814 | 7475.703 | 50.45112 | 8.34E-03 | 0.192052 | 196.6121 |
|  | 1438.707 | 99.69635 | 8.704307 | 610.8671 | 7151.459 | 47.71984 | 7.45E-03 | 0.201177 | 113.27 |
|  | 1461.795 | 560.3897 | 46.62526 | 18426.75 | 8240.576 | 247.0823 | 4.18E-02 | 0.17739 | 296.2044 |
|  | 1469.621 | 45.05037 | 3.685051 | 154.5224 | 5745.54 | 35.59712 | 3.36E-03 | 0.255785 | 77.10908 |
|  | 1483.677 | 584.7262 | 46.43351 | 2610.637 | 8802.855 | 249.148 | 0.043666 | 0.168545 | 1629.148 |
|  | 1489.792 | 50.72342 | 3.968934 | 94.45597 | 10081.52 | 18.28359 | 3.79E-03 | 0.147775 | 165.3236 |
|  | 1493.747 | 169.8466 | 13.05008 | 1554.148 | 9470.558 | 66.48763 | 1.27E-02 | 0.157725 | 108.0017 |
|  | 1499.723 | 60.12433 | 4.617968 | 142.7537 | 12200.97 | 21.45925 | 4.49E-03 | 0.122918 | 142.9245 |
|  | 1509.806 | 3394.986 | 255.6732 | 145386 | 7531.07 | 1704.954 | 0.253529 | 0.200477 | 4445.074 |
|  | 1525.806 | 232.4919 | 19.04613 | 1543.77 | 10307.72 | 90.412 | 1.74E-02 | 0.148026 | 220.2138 |
|  | 1537.791 | 85.74885 | 7.466661 | 547.3518 | 10040.35 | 33.73527 | 6.40E-03 | 0.153161 | 85.42404 |
|  | 1566.822 | 128.1201 | 13.94775 | 1190.91 | 8412.431 | 56.46151 | 9.57E-03 | 0.186251 | 100.9906 |
|  | 1584.809 | 89.3889 | 11.21451 | 862.8194 | 8149.132 | 41.91005 | 6.68E-03 | 0.194476 | 71.62918 |
|  | 1638.862 | 37.90994 | 5.207367 | 215.1746 | 10586.57 | 19.47998 | 2.83E-03 | 0.154806 | 34.05179 |
|  | 1814.818 | 170.9963 | 27.79914 | 5305.694 | 11310.6 | 98.91528 | 1.28E-02 | 0.160453 | 88.96012 |
|  | 1842.881 | 35.46968 | 5.588924 | 392.069 | 10723.01 | 23.05885 | 2.65E-03 | 0.171862 | 28.68523 |
|  | 1941.95 | 349.1157 | 43.87656 | 2853.025 | 9115.459 | 228.6584 | 2.61E-02 | 0.213039 | 837.1806 |
|  | 1969.945 | 41.73569 | 4.898622 | 248.1382 | 8739.22 | 27.31458 | 3.12E-03 | 0.225414 | 44.55595 |
|  | 1990.927 | 379.476 | 46.28284 | 10132.86 | 9567.223 | 259.1005 | 2.83E-02 | 0.208099 | 405.0021 |
|  | 1998.406 | 124.8417 | 16.0196 | 1319.611 | 6516.872 | 143.2731 | 9.32E-03 | 0.306651 | 191.9356 |
|  | 1999.002 | 55.01494 | 6.871981 | 68.30621 | 9521.445 | 39.32173 | 4.11E-03 | 0.209947 | 159.1554 |
|  | 2038.922 | 1379.632 | 190.8871 | 25812.43 | 9381.062 | 983.6508 | 0.103027 | 0.217345 | 3732.417 |
|  | 2054.937 | 283.7736 | 40.00589 | 8575.519 | 9587.744 | 190.8527 | 2.12E-02 | 0.21433 | 271.4653 |
|  | 2111.018 | 50.22444 | 7.329713 | 232.8362 | 10791.57 | 37.39826 | 3.75E-03 | 0.195617 | 90.44018 |
|  | 2120.1 | 162.6433 | 23.86348 | 1665.643 | 9229.171 | 124.259 | 1.21E-02 | 0.229717 | 207.9998 |
|  | 2127.572 | 116.728 | 17.10762 | 1471.82 | 11518.16 | 91.78226 | 8.72E-03 | 0.184715 | 116.6827 |
|  | 2168.102 | 465.3702 | 78.0654 | 9861.964 | 9555.47 | 343.0198 | 3.48E-02 | 0.226896 | 714.4127 |
|  | 2184.121 | 180.6228 | 32.39125 | 1872.339 | 10404.67 | 128.7304 | 1.35E-02 | 0.209917 | 225.6275 |
|  | 2211.104 | 282.1756 | 56.79157 | 6401.611 | 11580.78 | 180.4939 | 2.11E-02 | 0.190929 | 603.97 |
|  | 2655.695 | 26.49834 | 13.34248 | 604.8738 | 8057.992 | 25.43389 | 1.98E-03 | 0.329573 | 21.16081 |
|  | 2696.362 | 45.67934 | 24.01393 | 697.7751 | 11667.57 | 34.4099 | 3.41E-03 | 0.231099 | 53.67026 |
|  | 2712.367 | 43.43594 | 26.06846 | 805.0912 | 11248.7 | 36.45872 | 3.24E-03 | 0.241127 | 40.91022 |
|  | 2993.474 | 409.8411 | 563.0875 | 1279.152 | 13167.44 | 375.6107 | 3.06E-02 | 0.227339 | 4301.42 |
|  |  |  |  |  |  |  |  |  |  |
| ID | m/z | Intens. | SN | Quality Fac. | Res. | Area | Rel. Intens. | FWHM | Chi^2 |
| 554 | 842.5094 | 76.81917 | 46.84881 | 424.1936 | 11959.82 | 11.83155 | 2.39E-03 | 7.04E-02 | 45.78453 |
|  | 1059.582 | 195.3302 | 85.67833 | 470.5291 | 11717.11 | 37.08993 | 6.09E-03 | 9.04E-02 | 330.9049 |
|  | 1065.54 | 1307.964 | 573.0496 | 637.8837 | 10165.56 | 267.2467 | 4.08E-02 | 0.104819 | 10267.4 |
|  | 1114.52 | 591.1396 | 223.5576 | 609.2752 | 10456.58 | 128.4888 | 1.84E-02 | 0.106586 | 2339.343 |
|  | 1146.514 | 75.21414 | 27.04489 | 513.8087 | 9347.79 | 18.17176 | 2.34E-03 | 0.122651 | 46.53412 |
|  | 1193.65 | 2229.698 | 678.8465 | 3609.161 | 9557.136 | 584.2252 | 6.95E-02 | 0.124896 | 23108.41 |
|  | 1248.71 | 77.03974 | 7.342551 | 567.1947 | 11232.95 | 20.47744 | 2.40E-03 | 0.111165 | 55.33751 |
|  | 1259.708 | 2631.145 | 175.1339 | 8324.134 | 9637.741 | 755.3467 | 8.20E-02 | 0.130706 | 16162.68 |
|  | 1263.704 | 152.5066 | 9.054332 | 1410.42 | 9200.917 | 49.67374 | 4.75E-03 | 0.137345 | 90.25339 |
|  | 1278.766 | 32096.58 | 1383.721 | 84720.84 | 7525.144 | 12290.66 | 1 | 0.169932 | 392416.2 |
|  | 1292.702 | 21851.56 | 758.1795 | 91816.61 | 8213.077 | 7791.409 | 0.680807 | 0.157396 | 217636.1 |
|  | 1308.696 | 4530.666 | 135.5185 | 9235.333 | 9768.597 | 1352.729 | 0.141157 | 0.13397 | 37172.52 |
|  | 1320.666 | 236.8033 | 8.256955 | 168.0591 | 9896.268 | 71.09212 | 7.38E-03 | 0.133451 | 2675.557 |
|  | 1324.686 | 2402.871 | 90.40629 | 1812.462 | 10979.27 | 655.0942 | 7.49E-02 | 0.120653 | 17139.21 |
|  | 1335.775 | 152.3305 | 6.782816 | 1519.031 | 11714.5 | 40.9713 | 4.75E-03 | 0.114027 | 80.44326 |
|  | 1428.749 | 59.96813 | 9.086821 | 177.3967 | 10730.29 | 20.73846 | 1.87E-03 | 0.133151 | 150.1994 |
|  | 1431.87 | 59.71808 | 8.816212 | 54.55277 | 12176.28 | 18.84698 | 1.86E-03 | 0.117595 | 405.8258 |
|  | 1432.843 | 134.3896 | 19.7581 | 246.7915 | 8753.402 | 52.23306 | 4.19E-03 | 0.16369 | 466.5692 |
|  | 1434.755 | 79.44978 | 11.35248 | 183.813 | 9716.497 | 30.13407 | 2.48E-03 | 0.147662 | 231.3188 |
|  | 1448.795 | 4485.823 | 570.0046 | 22621.81 | 10736.61 | 1480.065 | 0.13976 | 0.13494 | 32214.67 |
|  | 1464.786 | 579.6561 | 72.65415 | 2678.067 | 11736.47 | 180.0668 | 1.81E-02 | 0.124806 | 744.1498 |
|  | 1480.778 | 572.4936 | 81.5512 | 1370.221 | 13389.33 | 162.3319 | 1.78E-02 | 0.110594 | 1408.86 |
|  | 1487.92 | 96.65041 | 14.68033 | 566.1442 | 13301.09 | 28.29559 | 3.01E-03 | 0.111864 | 90.27135 |
|  | 1493.766 | 63.29062 | 10.10744 | 602.3414 | 11542.87 | 19.91435 | 1.97E-03 | 0.12941 | 41.16043 |
|  | 1534.897 | 232.4334 | 63.91703 | 866.9879 | 13850.7 | 65.83337 | 7.24E-03 | 0.110817 | 366.9965 |
|  | 1562.895 | 593.416 | 188.9757 | 1962.897 | 13186.79 | 188.0795 | 1.85E-02 | 0.11852 | 1736.136 |
|  | 1699.944 | 263.5069 | 83.83238 | 983.9295 | 15222.58 | 82.25125 | 8.21E-03 | 0.111673 | 489.6917 |
|  | 1739.949 | 208.9781 | 38.03458 | 524.5603 | 13692.03 | 81.79081 | 6.51E-03 | 0.127078 | 623.5067 |
|  | 1741.958 | 153.6595 | 27.19032 | 638.2747 | 10244.96 | 86.49485 | 4.79E-03 | 0.170031 | 387.766 |
|  | 1757.968 | 24584.02 | 3729.332 | 41886.59 | 9734.998 | 13487.12 | 0.765939 | 0.180582 | 354138.4 |
|  | 1785.945 | 175.4806 | 21.20029 | 1052.047 | 15219.96 | 61.82043 | 5.47E-03 | 0.117342 | 227.4412 |
|  | 1879.943 | 735.3044 | 342.3497 | 2852.322 | 16236.54 | 279.2914 | 0.022909 | 0.115785 | 4579.64 |
|  | 1917.959 | 69.49458 | 32.98365 | 847.7408 | 15335.1 | 26.04993 | 2.17E-03 | 0.12507 | 48.78667 |
|  | 1965.949 | 699.8541 | 363.1555 | 3687.217 | 16458.53 | 270.6293 | 2.18E-02 | 0.119449 | 3321.253 |
|  | 1981.936 | 42.64431 | 21.95685 | 601.9972 | 17412.02 | 14.38171 | 1.33E-03 | 0.113826 | 25.81332 |
|  | 2056.136 | 1192.296 | 658.1801 | 6097.885 | 16642.92 | 503.4157 | 3.71E-02 | 0.123544 | 12641.53 |
|  | 2084.123 | 140.2798 | 87.55854 | 1024.355 | 17105.36 | 54.24481 | 4.37E-03 | 0.12184 | 169.8315 |
|  | 2191.112 | 735.723 | 643.2176 | 5772.739 | 16491.37 | 323.4733 | 2.29E-02 | 0.132864 | 6343.192 |
|  | 2199.162 | 487.0747 | 425.8474 | 2177.062 | 17359.55 | 200.4761 | 1.52E-02 | 0.126683 | 4689.842 |
|  | 2560.231 | 48.04148 | 21.93522 | 651.9696 | 14332.08 | 26.84053 | 1.50E-03 | 0.178636 | 46.89623 |
|  | 2578.299 | 1119.538 | 550.2211 | 12334.82 | 17230.55 | 589.009 | 3.49E-02 | 0.149635 | 10953.83 |
|  | 2594.301 | 57.14307 | 30.39241 | 601.1597 | 20903.26 | 24.61927 | 1.78E-03 | 0.12411 | 68.62345 |
|  | 2642.343 | 35.62911 | 23.64226 | 211.4861 | 17719.12 | 24.87389 | 0.00111 | 0.149124 | 78.70481 |
|  | 2756.288 | 173.4499 | 48.95704 | 851.2244 | 17691 | 102.708 | 5.40E-03 | 0.155802 | 612.9783 |
|  | 2763.592 | 79.37716 | 24.20519 | 760.6519 | 12751.88 | 65.51752 | 2.47E-03 | 0.21672 | 157.5319 |
|  | 2804.302 | 3678.498 | 1500.986 | 4327.671 | 15446.52 | 2568.782 | 0.114607 | 0.181549 | 73353.99 |
|  | 2820.298 | 304.6925 | 143.3557 | 4031.451 | 20125.87 | 158.4048 | 9.49E-03 | 0.140133 | 1537.908 |
|  | 2960.42 | 111.1925 | 126.158 | 1442.224 | 22136.41 | 58.93216 | 3.46E-03 | 0.133735 | 487.0192 |
|  |  |  |  |  |  |  |  |  |  |
| ID | m/z | Intens. | SN | Quality Fac. | Res. | Area | Rel. Intens. | FWHM | Chi^2 |
| 583 | 804.2672 | 70.56246 | 8.644974 | 276.2583 | 7166.691 | 16.57745 | 2.71E-03 | 0.112223 | 64.9628 |
|  | 819.4705 | 9634.353 | 1105.706 | 9908.818 | 5881.613 | 2311.282 | 0.370277 | 0.139328 | 55471.3 |
|  | 838.4727 | 72.36986 | 8.440463 | 303.0832 | 7281.17 | 13.58795 | 2.78E-03 | 0.115156 | 53.90145 |
|  | 842.5094 | 2323.223 | 275.549 | 1376.296 | 6549.54 | 513.2218 | 8.93E-02 | 0.128636 | 13479.27 |
|  | 856.5213 | 124.2649 | 15.71544 | 758.0694 | 8205.187 | 23.7725 | 4.78E-03 | 0.104388 | 67.95056 |
|  | 870.5446 | 652.9953 | 87.03823 | 791.5581 | 7111.281 | 136.9868 | 2.51E-02 | 0.122417 | 2023.258 |
|  | 911.5215 | 84.92366 | 12.89227 | 442.1535 | 8629.393 | 17.33508 | 3.26E-03 | 0.10563 | 56.52221 |
|  | 1004.591 | 3065.783 | 464.5047 | 2820.54 | 6901.917 | 832.1192 | 0.117827 | 0.145552 | 20020.27 |
|  | 1030.565 | 116.5864 | 17.45752 | 186.7171 | 7201.049 | 29.08191 | 4.48E-03 | 0.143113 | 337.1673 |
|  | 1032.585 | 637.2771 | 96.06876 | 2866.257 | 7323.093 | 171.5356 | 2.45E-02 | 0.141004 | 601.4142 |
|  | 1042.565 | 100.5643 | 14.85339 | 289.7175 | 7937.825 | 24.68809 | 3.86E-03 | 0.131341 | 155.802 |
|  | 1045.565 | 355.5253 | 52.32086 | 1415.502 | 8196.33 | 87.6907 | 1.37E-02 | 0.127565 | 404.2869 |
|  | 1114.655 | 113.1802 | 20.63411 | 718.1445 | 7776.089 | 32.11381 | 4.35E-03 | 0.143344 | 79.85295 |
|  | 1294.629 | 106.8878 | 13.60397 | 800.6578 | 9850.201 | 29.37687 | 4.11E-03 | 0.131432 | 69.95933 |
|  | 1315.651 | 1076.421 | 144.015 | 3649.961 | 9138.635 | 366.0451 | 4.14E-02 | 0.143966 | 1879.436 |
|  | 1324.791 | 4079.482 | 579.1298 | 13719.35 | 8453.707 | 1457.815 | 0.156787 | 0.156711 | 23774.18 |
|  | 1475.752 | 81.74167 | 13.59683 | 564.3927 | 11343.96 | 31.75168 | 3.14E-03 | 0.130091 | 76.21133 |
|  | 1492.786 | 164.646 | 24.84971 | 844.591 | 7128.021 | 88.74869 | 6.33E-03 | 0.209425 | 281.4792 |
|  | 1509.89 | 92.98883 | 12.83176 | 736.1813 | 8942.474 | 39.67027 | 3.57E-03 | 0.168845 | 77.18645 |
|  | 1545.873 | 5209.152 | 200.5612 | 77521.36 | 7967.911 | 2718.361 | 0.200203 | 0.194012 | 12850.42 |
|  | 1553.559 | 5579.294 | 185.2347 | 7230.352 | 6243.2 | 3921.524 | 0.214429 | 0.24884 | 36887.18 |
|  | 1575.858 | 262.1682 | 6.256098 | 843.1016 | 9148.37 | 116.2954 | 1.01E-02 | 0.172256 | 606.5117 |
|  | 1593.88 | 26019.31 | 669.6226 | 125823.8 | 6941.033 | 16450.94 | 1 | 0.229632 | 156013.1 |
|  | 1596.547 | 213.0205 | 5.657988 | 90.19558 | 6319.537 | 124.0365 | 8.19E-03 | 0.252637 | 5530.768 |
|  | 1609.886 | 10116.05 | 335.5832 | 149074.7 | 8025.941 | 5632.642 | 0.38879 | 0.200585 | 32959.31 |
|  | 1625.884 | 153.6347 | 7.039078 | 995.2719 | 11090.7 | 61.93116 | 5.90E-03 | 0.146599 | 166.3036 |
|  | 1636.888 | 109.9198 | 7.089316 | 265.473 | 10730.14 | 45.77745 | 4.22E-03 | 0.15255 | 362.8752 |
|  | 1796.945 | 200.1456 | 40.08135 | 1909.462 | 10924.73 | 101.6676 | 7.69E-03 | 0.164484 | 197.3632 |
|  | 1844.939 | 953.7346 | 242.7169 | 4728.221 | 12217.5 | 481.1245 | 3.67E-02 | 0.151008 | 4976.867 |
|  | 1860.924 | 84.93717 | 24.39676 | 1895.348 | 13354.81 | 38.26536 | 3.26E-03 | 0.139345 | 32.23597 |
|  | 2211.104 | 1083.515 | 504.8553 | 5791.463 | 13045.33 | 599.1066 | 4.16E-02 | 0.169494 | 11175.79 |
|  | 2222.122 | 41.86208 | 18.31064 | 299.6248 | 10851.82 | 22.52081 | 1.61E-03 | 0.204769 | 73.57227 |
|  | 2495.338 | 174.9384 | 176.7141 | 2814.274 | 14038.16 | 104.1449 | 6.72E-03 | 0.177754 | 494.3802 |
|  | 2512.359 | 193.4193 | 219.7891 | 1591.767 | 15354.96 | 112.2086 | 7.43E-03 | 0.163619 | 953.0204 |
|  | 752.353 | 88.82426 | 15.90816 | 490.5587 | 6214.092 | 16.01589 | 2.78E-02 | 0.121072 | 56.43282 |
|  | 774.4208 | 31.10696 | 5.767017 | 144.0127 | 7367.27 | 5.826767 | 9.74E-03 | 0.105116 | 14.10552 |
|  | 804.2661 | 53.23109 | 9.847686 | 257.526 | 12395.43 | 7.275742 | 1.67E-02 | 0.064884 | 31.97884 |
|  | 838.4729 | 37.54733 | 6.913353 | 228.6166 | 9550.251 | 7.681386 | 1.18E-02 | 8.78E-02 | 22.3095 |
|  | 856.5245 | 125.7539 | 24.37569 | 452.4733 | 12106.14 | 15.65644 | 3.94E-02 | 7.08E-02 | 96.08874 |
|  | 870.5462 | 1118.708 | 227.8916 | 2196.151 | 9748.282 | 172.7476 | 0.350204 | 8.93E-02 | 5502.01 |
|  | 932.4476 | 52.14037 | 11.77086 | 316.7304 | 9023.996 | 9.782632 | 1.63E-02 | 0.10333 | 30.14401 |
|  | 950.5218 | 41.24667 | 9.223823 | 275.2579 | 7975.326 | 8.504149 | 1.29E-02 | 0.119183 | 22.61008 |
|  | 979.531 | 300.7622 | 70.2215 | 750.7765 | 12081.34 | 47.75728 | 0.094151 | 8.11E-02 | 385.4519 |
|  | 995.5331 | 34.66856 | 8.244812 | 133.5484 | 17717.02 | 6.094903 | 1.09E-02 | 5.62E-02 | 30.99266 |
|  | 1209.638 | 1408.857 | 372.5224 | 7867.303 | 10666.06 | 329.5193 | 0.441033 | 0.11341 | 8686.85 |
|  | 1219.689 | 14.09052 | 3.798746 | 66.36872 | 8101.821 | 4.461837 | 4.41E-03 | 0.150545 | 7.126248 |
|  | 1277.721 | 16.39641 | 4.38707 | 118.2871 | 6759.132 | 6.925515 | 5.13E-03 | 0.189036 | 7.562558 |
|  | 1317.717 | 33.1921 | 9.096087 | 262.1386 | 13897.12 | 7.915683 | 1.04E-02 | 9.48E-02 | 19.13353 |
|  | 1327.739 | 35.05866 | 9.595824 | 513.9289 | 11164.34 | 10.2541 | 1.10E-02 | 0.118927 | 13.1892 |
|  | 1439.779 | 15.34859 | 4.867193 | 183.213 | 12568.48 | 5.119606 | 4.80E-03 | 0.114555 | 4.135037 |
|  | 1503.884 | 278.1273 | 83.55697 | 6898.851 | 13617.83 | 75.82287 | 8.71E-02 | 0.110435 | 407.5928 |
|  | 1534.831 | 194.0011 | 54.66434 | 2166.857 | 13783.14 | 53.04036 | 6.07E-02 | 0.111356 | 360.5217 |
|  | 1549.76 | 295.15 | 88.98607 | 6893.461 | 15080.43 | 87.73551 | 9.24E-02 | 0.102766 | 458.3746 |
|  | 1610.815 | 13.91059 | 5.107013 | 199.621 | 15507.85 | 5.050588 | 0.004355 | 0.103871 | 6.754894 |
|  | 1626.801 | 26.4446 | 10.01135 | 454.636 | 14121.8 | 8.865956 | 8.28E-03 | 0.115198 | 10.09888 |
|  | 1885.847 | 438.5429 | 225.5774 | 4248.661 | 15572.92 | 164.7611 | 0.137283 | 0.121098 | 1516.236 |
|  | 1901.838 | 29.21407 | 16.54025 | 357.7422 | 15129.32 | 10.31117 | 9.15E-03 | 0.125705 | 20.66351 |
|  | 1998.811 | 28.7128 | 27.1311 | 300.1079 | 14081.4 | 12.53859 | 8.99E-03 | 0.141947 | 26.35172 |
|  | 2100.969 | 70.48125 | 79.50841 | 3112.414 | 17684.68 | 25.83642 | 2.21E-02 | 0.118802 | 82.64741 |
|  | 2225.1 | 9.681575 | 9.852173 | 121.4264 | 19651.99 | 3.891565 | 3.03E-03 | 0.113225 | 5.44896 |
|  | 2233.072 | 14.16664 | 14.58716 | 301.2176 | 18531.99 | 5.261028 | 4.43E-03 | 0.120498 | 6.504701 |
|  | 2438.244 | 37.10118 | 69.51494 | 2395.227 | 20462.13 | 14.80332 | 1.16E-02 | 0.119159 | 27.66981 |
|  |  |  |  |  |  |  |  |  |  |
| ID | m/z | Intens. | SN | Quality Fac. | Res. | Area | Rel. Intens. | FWHM | Chi^2 |
| 618 | 752.353 | 88.82426 | 15.90816 | 490.5587 | 6214.092 | 16.01589 | 2.78E-02 | 0.121072 | 56.43282 |
|  | 774.4208 | 31.10696 | 5.767017 | 144.0127 | 7367.27 | 5.826767 | 9.74E-03 | 0.105116 | 14.10552 |
|  | 804.2661 | 53.23109 | 9.847686 | 257.526 | 12395.43 | 7.275742 | 1.67E-02 | 0.064884 | 31.97884 |
|  | 838.4729 | 37.54733 | 6.913353 | 228.6166 | 9550.251 | 7.681386 | 1.18E-02 | 8.78E-02 | 22.3095 |
|  | 856.5245 | 125.7539 | 24.37569 | 452.4733 | 12106.14 | 15.65644 | 3.94E-02 | 7.08E-02 | 96.08874 |
|  | 870.5462 | 1118.708 | 227.8916 | 2196.151 | 9748.282 | 172.7476 | 0.350204 | 8.93E-02 | 5502.01 |
|  | 932.4476 | 52.14037 | 11.77086 | 316.7304 | 9023.996 | 9.782632 | 1.63E-02 | 0.10333 | 30.14401 |
|  | 950.5218 | 41.24667 | 9.223823 | 275.2579 | 7975.326 | 8.504149 | 1.29E-02 | 0.119183 | 22.61008 |
|  | 979.531 | 300.7622 | 70.2215 | 750.7765 | 12081.34 | 47.75728 | 0.094151 | 8.11E-02 | 385.4519 |
|  | 995.5331 | 34.66856 | 8.244812 | 133.5484 | 17717.02 | 6.094903 | 1.09E-02 | 5.62E-02 | 30.99266 |
|  | 1209.638 | 1408.857 | 372.5224 | 7867.303 | 10666.06 | 329.5193 | 0.441033 | 0.11341 | 8686.85 |
|  | 1219.689 | 14.09052 | 3.798746 | 66.36872 | 8101.821 | 4.461837 | 4.41E-03 | 0.150545 | 7.126248 |
|  | 1277.721 | 16.39641 | 4.38707 | 118.2871 | 6759.132 | 6.925515 | 5.13E-03 | 0.189036 | 7.562558 |
|  | 1317.717 | 33.1921 | 9.096087 | 262.1386 | 13897.12 | 7.915683 | 1.04E-02 | 9.48E-02 | 19.13353 |
|  | 1327.739 | 35.05866 | 9.595824 | 513.9289 | 11164.34 | 10.2541 | 1.10E-02 | 0.118927 | 13.1892 |
|  | 1439.779 | 15.34859 | 4.867193 | 183.213 | 12568.48 | 5.119606 | 4.80E-03 | 0.114555 | 4.135037 |
|  | 1503.884 | 278.1273 | 83.55697 | 6898.851 | 13617.83 | 75.82287 | 8.71E-02 | 0.110435 | 407.5928 |
|  | 1534.831 | 194.0011 | 54.66434 | 2166.857 | 13783.14 | 53.04036 | 6.07E-02 | 0.111356 | 360.5217 |
|  | 1549.76 | 295.15 | 88.98607 | 6893.461 | 15080.43 | 87.73551 | 9.24E-02 | 0.102766 | 458.3746 |
|  | 1610.815 | 13.91059 | 5.107013 | 199.621 | 15507.85 | 5.050588 | 0.004355 | 0.103871 | 6.754894 |
|  | 1626.801 | 26.4446 | 10.01135 | 454.636 | 14121.8 | 8.865956 | 8.28E-03 | 0.115198 | 10.09888 |
|  | 1885.847 | 438.5429 | 225.5774 | 4248.661 | 15572.92 | 164.7611 | 0.137283 | 0.121098 | 1516.236 |
|  | 1901.838 | 29.21407 | 16.54025 | 357.7422 | 15129.32 | 10.31117 | 9.15E-03 | 0.125705 | 20.66351 |
|  | 1998.811 | 28.7128 | 27.1311 | 300.1079 | 14081.4 | 12.53859 | 8.99E-03 | 0.141947 | 26.35172 |
|  | 2100.969 | 70.48125 | 79.50841 | 3112.414 | 17684.68 | 25.83642 | 2.21E-02 | 0.118802 | 82.64741 |
|  | 2225.1 | 9.681575 | 9.852173 | 121.4264 | 19651.99 | 3.891565 | 3.03E-03 | 0.113225 | 5.44896 |
|  | 2233.072 | 14.16664 | 14.58716 | 301.2176 | 18531.99 | 5.261028 | 4.43E-03 | 0.120498 | 6.504701 |
|  | 2438.244 | 37.10118 | 69.51494 | 2395.227 | 20462.13 | 14.80332 | 1.16E-02 | 0.119159 | 27.66981 |
|  |  |  |  |  |  |  |  |  |  |
|  |  |  |  |  |  |  |  |  |  |
|  |  |  |  |  |  |  |  |  |  |
|  |  |  |  |  |  |  |  |  |  |
|  |  |  |  |  |  |  |  |  |  |
|  |  |  |  |  |  |  |  |  |  |
|  |  |  |  |  |  |  |  |  |  |
| ID | m/z | Intens. | SN | Quality Fac. | Res. | Area | Rel. Intens. | FWHM | Chi^2 |
| 672 | 757.485 | 34.50552 | 5.010995 | 82.92071 | 5208.337 | 8.936558 | 1.89E-03 | 0.145437 | 32.53607 |
|  | 765.5665 | 79.47322 | 11.44774 | 72.47324 | 12462.06 | 8.280257 | 4.34E-03 | 6.14E-02 | 215.2614 |
|  | 767.556 | 45.51251 | 6.304906 | 71.23374 | 12580.93 | 4.253723 | 2.49E-03 | 6.10E-02 | 61.41007 |
|  | 773.3707 | 250.7383 | 35.77514 | 970.5183 | 8207.939 | 40.05353 | 1.37E-02 | 9.42E-02 | 203.7346 |
|  | 777.4332 | 750.3504 | 107.9448 | 1799.727 | 8164.508 | 129.1124 | 0.041012 | 9.52E-02 | 2327.215 |
|  | 804.2542 | 107.4002 | 16.01881 | 577.9879 | 7352.794 | 21.28392 | 5.87E-03 | 0.109381 | 64.08862 |
|  | 812.3255 | 54.06807 | 7.914776 | 73.36167 | 12743.51 | 5.656412 | 2.96E-03 | 6.37E-02 | 100.9663 |
|  | 856.5321 | 49.47315 | 7.31287 | 201.0067 | 7770.864 | 9.809683 | 2.70E-03 | 0.110224 | 33.56631 |
|  | 870.5496 | 254.0865 | 36.61276 | 632.9 | 8039.693 | 45.46755 | 1.39E-02 | 0.108281 | 375.5558 |
|  | 883.5337 | 2233.017 | 325.6073 | 4439.023 | 8863.957 | 410.5511 | 0.12205 | 9.97E-02 | 12956.68 |
|  | 913.5506 | 49.86378 | 7.963407 | 205.3434 | 9230.581 | 10.66799 | 2.73E-03 | 9.90E-02 | 43.08439 |
|  | 950.5136 | 39.50312 | 6.868864 | 160.1244 | 14796.16 | 6.083658 | 2.16E-03 | 6.42E-02 | 29.25444 |
|  | 964.5228 | 80.9101 | 14.67929 | 697.14 | 11353.9 | 13.10542 | 4.42E-03 | 8.50E-02 | 28.63799 |
|  | 1004.543 | 51.21476 | 9.322294 | 248.6628 | 8014.19 | 11.89061 | 2.80E-03 | 0.125346 | 40.79922 |
|  | 1048.543 | 26.57099 | 4.313277 | 61.42634 | 12388.83 | 4.618233 | 1.45E-03 | 8.46E-02 | 27.32583 |
|  | 1078.571 | 128.8214 | 18.00062 | 394.3925 | 8606.99 | 30.0672 | 7.04E-03 | 0.125313 | 165.1926 |
|  | 1095.6 | 33.54763 | 4.330659 | 86.53215 | 7542.443 | 11.47506 | 1.83E-03 | 0.145258 | 32.80393 |
|  | 1113.598 | 3855.003 | 454.613 | 14852.99 | 9199.171 | 1015.255 | 0.210703 | 0.121054 | 36595.71 |
|  | 1119.545 | 62.64796 | 6.855747 | 320.4933 | 10558.24 | 12.74711 | 3.42E-03 | 0.106035 | 42.4065 |
|  | 1127.541 | 32.69801 | 3.296702 | 98.77841 | 4066.674 | 17.57642 | 1.79E-03 | 0.277264 | 35.06996 |
|  | 1135.57 | 408.7226 | 37.74927 | 5899.188 | 8574.842 | 106.266 | 2.23E-02 | 0.13243 | 237.98 |
|  | 1144.595 | 106.9827 | 9.095491 | 609.267 | 11252.65 | 22.49757 | 5.85E-03 | 0.101718 | 70.36459 |
|  | 1147.564 | 76.37452 | 6.343785 | 237.4761 | 7235.291 | 24.08376 | 4.17E-03 | 0.158606 | 129.9081 |
|  | 1151.563 | 316.2118 | 25.17094 | 895.6213 | 12924.15 | 68.18251 | 1.73E-02 | 8.91E-02 | 426.7124 |
|  | 1162.591 | 18295.89 | 1329.81 | 76681.92 | 7916.966 | 5785.287 | 1 | 0.146848 | 171996.3 |
|  | 1167.565 | 221.3939 | 15.32111 | 487.5068 | 10331.16 | 50.7354 | 1.21E-02 | 0.113014 | 426.7342 |
|  | 1174.599 | 113.0465 | 7.602532 | 373.2864 | 11646.58 | 27.02983 | 6.18E-03 | 0.100854 | 153.2057 |
|  | 1181.724 | 123.8064 | 8.96348 | 361.5726 | 9346.116 | 28.422 | 6.77E-03 | 0.12644 | 181.0953 |
|  | 1183.557 | 345.067 | 25.57793 | 2338.383 | 10123.29 | 89.1806 | 1.89E-02 | 0.116914 | 316.2055 |
|  | 1206.613 | 2231.621 | 216.7704 | 31614.63 | 10001.57 | 644.3069 | 0.121974 | 0.120642 | 6600.151 |
|  | 1290.633 | 336.2976 | 42.78885 | 12074.4 | 10050.72 | 104.7075 | 1.84E-02 | 0.128412 | 153.2579 |
|  | 1294.63 | 51.29448 | 6.378805 | 223.6721 | 9426.245 | 15.70377 | 2.80E-03 | 0.137343 | 68.4516 |
|  | 1298.442 | 386.3362 | 47.12087 | 1638.548 | 7664.965 | 180.5276 | 2.11E-02 | 0.1694 | 648.9599 |
|  | 1320.743 | 969.1379 | 105.8213 | 16417.13 | 10098.46 | 306.0981 | 0.05297 | 0.130787 | 1533.235 |
|  | 1324.647 | 67.19308 | 7.068465 | 144.8727 | 9402.847 | 20.50429 | 3.67E-03 | 0.140877 | 166.2503 |
|  | 1338.63 | 8262.817 | 807.8543 | 57353.12 | 9065.779 | 2889.365 | 0.451622 | 0.147657 | 49542.73 |
|  | 1343.722 | 49.79515 | 4.837859 | 214.7909 | 8543.151 | 16.45342 | 2.72E-03 | 0.157286 | 57.21325 |
|  | 1354.631 | 1371.499 | 124.4008 | 13188.59 | 10831.33 | 409.0663 | 7.50E-02 | 0.125066 | 4559.04 |
|  | 1371.781 | 180.9561 | 15.7064 | 1182.758 | 10335.91 | 56.31549 | 9.89E-03 | 0.13272 | 145.3931 |
|  | 1399.723 | 590.2026 | 71.29993 | 7144.27 | 10500.64 | 179.9337 | 3.23E-02 | 0.133299 | 1060.97 |
|  | 1401.692 | 272.048 | 34.29762 | 786.7512 | 12873.36 | 68.89758 | 1.49E-02 | 0.108883 | 451.9864 |
|  | 1461.742 | 25.39444 | 7.48014 | 300.3258 | 9096.384 | 9.487623 | 1.39E-03 | 0.160695 | 11.4965 |
|  | 1478.776 | 83.80728 | 24.04817 | 1212.018 | 14656.91 | 24.76429 | 4.58E-03 | 0.100893 | 42.18989 |
|  | 1483.716 | 18.58883 | 5.423314 | 106.6741 | 11840.36 | 9.093458 | 1.02E-03 | 0.12531 | 25.28263 |
|  | 1532.826 | 54.72024 | 17.55466 | 717.1906 | 10334.35 | 20.327 | 2.99E-03 | 0.148323 | 26.75782 |
|  | 1575.801 | 16.06382 | 6.122068 | 111.7338 | 10812.59 | 5.794543 | 8.78E-04 | 0.145738 | 9.886596 |
|  | 1694.855 | 25.9398 | 10.78575 | 427.0603 | 9590.29 | 12.33639 | 1.42E-03 | 0.176726 | 13.12325 |
|  | 1722.949 | 814.5042 | 316.4971 | 15449.84 | 12581.74 | 312.5202 | 4.45E-02 | 0.13694 | 2444.088 |
|  | 1750.937 | 18.36603 | 7.851974 | 285.9808 | 11178.84 | 8.89699 | 1.00E-03 | 0.15663 | 10.39591 |
|  | 2079.157 | 57.97052 | 43.23061 | 1577.77 | 14747.05 | 25.93127 | 3.17E-03 | 0.140988 | 47.71677 |
|  | 2093.99 | 1521.803 | 1122.576 | 10480.4 | 14611.02 | 748.7948 | 0.083177 | 0.143316 | 16506.89 |
|  | 2300.143 | 11.77671 | 16.57682 | 192.8536 | 15915.94 | 6.956871 | 6.44E-04 | 0.144518 | 10.04948 |
|  |  |  |  |  |  |  |  |  |  |
| ID | m/z | Intens. | SN | Quality Fac. | Res. | Area | Rel. Intens. | FWHM | Chi^2 |
| 725 | 703.3701 | 30.92489 | 6.406199 | 83.20105 | 9919.277 | 3.688575 | 6.24E-03 | 7.09E-02 | 24.23163 |
|  | 733.3862 | 29.46234 | 6.199778 | 114.9022 | 8019.428 | 4.351211 | 5.94E-03 | 9.15E-02 | 14.70768 |
|  | 742.4479 | 1209.843 | 258.3545 | 2747.185 | 8015.107 | 181.0708 | 0.244123 | 9.26E-02 | 6445.955 |
|  | 781.3949 | 1001.048 | 219.9627 | 8344.023 | 7583.145 | 165.6467 | 0.201992 | 0.103044 | 2322.869 |
|  | 831.4463 | 25.24694 | 5.591252 | 76.15276 | 6904.839 | 5.465844 | 5.09E-03 | 0.120415 | 22.33548 |
|  | 833.432 | 25.00784 | 5.475917 | 74.58962 | 9001.059 | 4.504277 | 5.05E-03 | 9.26E-02 | 22.12006 |
|  | 834.424 | 30.775 | 6.791083 | 128.5521 | 9334.161 | 4.11116 | 6.21E-03 | 8.94E-02 | 21.77889 |
|  | 838.4588 | 27.36749 | 6.01104 | 188.7193 | 8170.511 | 4.817085 | 5.52E-03 | 0.10262 | 11.16017 |
|  | 870.5347 | 546.3837 | 128.8492 | 2882.77 | 9597.362 | 86.2887 | 0.11025 | 9.07E-02 | 1159.049 |
|  | 912.5296 | 62.29975 | 15.52458 | 613.6502 | 9930.551 | 10.29412 | 1.26E-02 | 0.091891 | 26.15099 |
|  | 941.6213 | 4955.882 | 1204.557 | 11858.54 | 8537.042 | 980.9918 | 1 | 0.110298 | 60385.86 |
|  | 1084.58 | 52.34928 | 12.52603 | 398.5585 | 10538.24 | 11.90858 | 1.06E-02 | 0.102919 | 32.43769 |
|  | 1100.561 | 40.78564 | 9.690806 | 319.2442 | 10348.45 | 8.462119 | 8.23E-03 | 0.10635 | 20.63102 |
|  | 1205.654 | 274.2202 | 65.75007 | 1888.406 | 10079.55 | 71.29556 | 5.53E-02 | 0.119614 | 880.2894 |
|  | 1206.688 | 197.2447 | 47.38598 | 454.6822 | 10647.54 | 38.4606 | 0.0398 | 0.11333 | 560.6715 |
|  | 1222.68 | 86.96042 | 20.14407 | 920.2701 | 11167.68 | 19.34998 | 1.75E-02 | 0.109484 | 34.74699 |
|  | 1259.708 | 23.42963 | 5.593057 | 107.4702 | 8244.091 | 7.548859 | 4.73E-03 | 0.152801 | 15.67011 |
|  | 1300.722 | 47.09771 | 11.46541 | 729.0247 | 10309.88 | 13.258 | 9.50E-03 | 0.126163 | 13.95163 |
|  | 1327.735 | 37.82112 | 9.301226 | 288.0291 | 9310.986 | 12.00787 | 7.63E-03 | 0.142599 | 25.87937 |
|  | 1508.796 | 540.1872 | 122.6845 | 9831.933 | 14263.44 | 158.7223 | 0.108999 | 0.105781 | 1517.244 |
|  | 1515.769 | 22.5134 | 5.074558 | 199.1832 | 11314.89 | 7.520952 | 4.54E-03 | 0.133962 | 14.26487 |
|  | 1556.823 | 69.46995 | 16.47094 | 2232.677 | 12532.36 | 22.43412 | 1.40E-02 | 0.124224 | 26.99329 |
|  | 1563.752 | 44.27045 | 10.84727 | 638.4128 | 11718.17 | 16.51981 | 8.93E-03 | 0.133447 | 20.64834 |
|  | 1579.763 | 53.6621 | 14.17651 | 667.2714 | 15403.29 | 15.53145 | 1.08E-02 | 0.10256 | 25.74208 |
|  | 1595.756 | 35.82555 | 10.45078 | 444.467 | 13364.93 | 13.02927 | 7.23E-03 | 0.119399 | 20.3227 |
|  | 1673.851 | 13.51593 | 4.069154 | 160.8414 | 10612.74 | 7.898736 | 2.73E-03 | 0.157721 | 6.528879 |
|  | 1708.831 | 2184.986 | 626.9343 | 13440.59 | 12611.73 | 825.7485 | 0.440887 | 0.135495 | 21485.91 |
|  | 1845 | 42.03242 | 15.39333 | 706.33 | 13830.74 | 17.24555 | 8.48E-03 | 0.133399 | 42.88109 |
|  | 1861.944 | 10.70118 | 3.972407 | 163.6881 | 12072.31 | 5.167752 | 2.16E-03 | 0.154233 | 3.151009 |
|  | 1867.012 | 15.12078 | 5.616919 | 128.088 | 13215.61 | 6.645909 | 3.05E-03 | 0.141273 | 10.61856 |
|  | 1873.021 | 24.73803 | 9.331846 | 462.5888 | 15239.26 | 10.79869 | 4.99E-03 | 0.122908 | 12.08814 |
|  | 2038.016 | 21.63178 | 12.73352 | 330.46 | 15920.3 | 9.43569 | 4.36E-03 | 0.128014 | 12.72037 |
|  | 2233.086 | 14.76375 | 10.65715 | 352.7505 | 12705.55 | 8.819399 | 2.98E-03 | 0.175757 | 7.795224 |
|  |  |  |  |  |  |  |  |  |  |
|  |  |  |  |  |  |  |  |  |  |
|  |  |  |  |  |  |  |  |  |  |
|  |  |  |  |  |  |  |  |  |  |
|  |  |  |  |  |  |  |  |  |  |
|  |  |  |  |  |  |  |  |  |  |
|  |  |  |  |  |  |  |  |  |  |
|  |  |  |  |  |  |  |  |  |  |
|  |  |  |  |  |  |  |  |  |  |
| ID | m/z | Intens. | SN | Quality Fac. | Res. | Area | Rel. Intens. | FWHM | Chi^2 |
| 728 | 743.4526 | 51.08335 | 14.03301 | 220.6566 | 7411.59 | 8.835499 | 2.16E-03 | 0.100309 | 35.94726 |
|  | 748.4 | 59.55455 | 16.29516 | 182.8101 | 6092.855 | 12.59159 | 2.51E-03 | 0.122832 | 67.3346 |
|  | 756.9118 | 176.5517 | 48.15869 | 279.001 | 10476.07 | 22.93665 | 7.45E-03 | 7.23E-02 | 316.0204 |
|  | 758.9123 | 52.07769 | 13.88758 | 126.4235 | 9400.224 | 6.71802 | 2.20E-03 | 8.07E-02 | 62.55864 |
|  | 764.3959 | 214.4898 | 58.48888 | 414.2236 | 6625.115 | 48.83105 | 9.05E-03 | 0.115379 | 428.5305 |
|  | 765.5504 | 166.2655 | 44.20904 | 143.3462 | 11238.82 | 18.03544 | 7.02E-03 | 6.81E-02 | 490.3223 |
|  | 767.5573 | 101.5547 | 27.52466 | 165.4194 | 9507.135 | 13.91097 | 4.29E-03 | 8.07E-02 | 193.4666 |
|  | 810.2987 | 99.73959 | 29.117 | 52.13867 | 11116.26 | 12.52736 | 4.21E-03 | 7.29E-02 | 528.8787 |
|  | 812.2987 | 143.3644 | 41.72222 | 134.5762 | 11638.05 | 17.46423 | 6.05E-03 | 6.98E-02 | 426.9188 |
|  | 814.3021 | 56.30711 | 16.27418 | 92.47832 | 14290.35 | 5.488735 | 2.38E-03 | 5.70E-02 | 86.5933 |
|  | 858.4486 | 56.72044 | 15.72199 | 686.349 | 8824.307 | 10.14938 | 2.39E-03 | 9.73E-02 | 14.46624 |
|  | 863.4284 | 74.17964 | 20.13441 | 446.3879 | 7761.019 | 14.2921 | 3.13E-03 | 0.111252 | 44.62148 |
|  | 870.535 | 81.40639 | 21.85647 | 295.534 | 7562.596 | 17.10698 | 3.43E-03 | 0.115111 | 86.23129 |
|  | 876.465 | 1247.574 | 325.7798 | 4474.168 | 6779.362 | 274.0782 | 5.26E-02 | 0.129284 | 5355.327 |
|  | 892.4945 | 79.30895 | 20.92093 | 456.5879 | 8579.206 | 14.79221 | 3.35E-03 | 0.10403 | 48.16055 |
|  | 904.4589 | 41.02012 | 11.35621 | 208.2709 | 12201.25 | 8.166739 | 1.73E-03 | 0.074128 | 32.32704 |
|  | 919.4986 | 218.4485 | 62.70023 | 4865.039 | 6875.487 | 50.65035 | 9.22E-03 | 0.133736 | 66.77124 |
|  | 927.5436 | 116.2688 | 34.63393 | 823.1364 | 6558.333 | 33.79966 | 4.91E-03 | 0.14143 | 73.56596 |
|  | 934.4795 | 45.04993 | 13.63175 | 247.7887 | 8707.335 | 9.391035 | 1.90E-03 | 0.107321 | 30.24292 |
|  | 967.5 | 3330.137 | 887.7506 | 14567.02 | 7601.365 | 783.2072 | 0.140517 | 0.12728 | 13515.48 |
|  | 983.5036 | 693.418 | 172.6588 | 986.9073 | 8483.343 | 146.452 | 2.93E-02 | 0.115933 | 1810.661 |
|  | 991.5319 | 141.8277 | 34.49293 | 711.7897 | 11315.99 | 24.4146 | 5.98E-03 | 8.76E-02 | 96.2981 |
|  | 1034.557 | 49.18912 | 13.85474 | 50.32895 | 14860.83 | 10.44778 | 2.08E-03 | 6.96E-02 | 190.5927 |
|  | 1035.527 | 189.1395 | 53.15268 | 815.5777 | 8586.695 | 43.3007 | 7.98E-03 | 0.120597 | 181.4711 |
|  | 1048.111 | 112.2737 | 32.05557 | 97.88804 | 12378.64 | 17.37638 | 4.74E-03 | 8.47E-02 | 479.7102 |
|  | 1083.516 | 820.301 | 254.761 | 2391.789 | 8876.049 | 198.1361 | 3.46E-02 | 0.122072 | 2993.682 |
|  | 1099.516 | 61.95771 | 19.8361 | 497.5576 | 8942.513 | 15.76799 | 2.61E-03 | 0.122954 | 37.07637 |
|  | 1258.659 | 43.88756 | 6.804955 | 431.8865 | 9789.808 | 11.64962 | 1.85E-03 | 0.128568 | 22.95475 |
|  | 1302.678 | 12617.58 | 809.512 | 109304.2 | 7927.967 | 4620.759 | 0.532408 | 0.164314 | 62191.25 |
|  | 1316.671 | 239.9215 | 18.1467 | 56.2962 | 9800.836 | 73.76473 | 1.01E-02 | 0.134343 | 6142.538 |
|  | 1318.674 | 1743.639 | 133.9714 | 9205.994 | 9636.56 | 530.836 | 7.36E-02 | 0.136841 | 7582.59 |
|  | 1334.665 | 6321.821 | 621.0711 | 29117.5 | 8770.65 | 2143.181 | 0.266754 | 0.152174 | 60574.66 |
|  | 1358.733 | 1197.904 | 202.9769 | 5501.348 | 11902.01 | 316.6001 | 5.05E-02 | 0.11416 | 2093.344 |
|  | 1398.778 | 51.17898 | 11.53663 | 704.0101 | 11096.23 | 15.49803 | 2.16E-03 | 0.126059 | 19.78361 |
|  | 1408.584 | 31.93986 | 6.943042 | 165.4704 | 20858.52 | 9.937739 | 1.35E-03 | 6.75E-02 | 39.27122 |
|  | 1416.754 | 43.87583 | 9.16554 | 488.3314 | 10520.18 | 12.81123 | 1.85E-03 | 0.13467 | 22.26954 |
|  | 1444.783 | 121.8932 | 19.09491 | 1136.275 | 10428.54 | 41.46954 | 5.14E-03 | 0.138541 | 77.84012 |
|  | 1462.737 | 91.84236 | 8.926243 | 250.4648 | 11179.13 | 31.56841 | 3.88E-03 | 0.130845 | 234.9705 |
|  | 1465.767 | 693.4982 | 63.25604 | 1968.145 | 12103.82 | 209.1314 | 2.93E-02 | 0.1211 | 1499.174 |
|  | 1471.821 | 388.2802 | 31.45361 | 2814.74 | 11112.06 | 124.4528 | 1.64E-02 | 0.132453 | 299.4424 |
|  | 1474.762 | 94.21171 | 7.214722 | 521.7457 | 8332.902 | 44.62229 | 3.98E-03 | 0.176981 | 124.1739 |
|  | 1478.745 | 150.5426 | 10.89056 | 105.9148 | 12222.24 | 46.70092 | 6.35E-03 | 0.120988 | 1450.334 |
|  | 1489.841 | 23699.1 | 1459.72 | 23858.59 | 7665.73 | 11660.74 | 1 | 0.194351 | 195423.7 |
|  | 1494.783 | 94.38107 | 5.473195 | 97.54825 | 8211.137 | 45.82104 | 3.98E-03 | 0.182043 | 778.0288 |
|  | 1497.756 | 1380.704 | 76.88225 | 2541.337 | 12361.41 | 422.1521 | 5.83E-02 | 0.121164 | 4710.764 |
|  | 1505.809 | 111.8239 | 5.647259 | 652.1475 | 10832.9 | 43.21388 | 4.72E-03 | 0.139003 | 130.6258 |
|  | 1511.754 | 189.9922 | 9.325502 | 1411.899 | 8537.158 | 85.9614 | 8.02E-03 | 0.177079 | 198.3842 |
|  | 1517.832 | 701.7538 | 37.06996 | 3581.229 | 10957.85 | 238.4364 | 2.96E-02 | 0.138516 | 851.6354 |
|  | 1543.827 | 54.63749 | 4.27448 | 111.3581 | 11415.15 | 19.4174 | 2.31E-03 | 0.135244 | 185.1745 |
|  | 1608.896 | 198.9915 | 40.75422 | 1292.236 | 14107 | 69.97605 | 8.40E-03 | 0.114049 | 230.4231 |
|  | 1618.806 | 830.0852 | 155.1146 | 7607.142 | 13295.87 | 287.0159 | 3.50E-02 | 0.121753 | 2331.531 |
|  | 1626.912 | 6156.73 | 1068.903 | 50457.12 | 9632.534 | 2801.904 | 0.259788 | 0.168898 | 39107.4 |
|  | 1637.854 | 42.13935 | 6.646785 | 117.5517 | 12538.66 | 17.26342 | 1.78E-03 | 0.130624 | 69.54459 |
|  | 1654.907 | 470.8885 | 66.76715 | 2407.968 | 14045.88 | 164.9652 | 1.99E-02 | 0.117822 | 1006.83 |
|  | 1682.906 | 34.54331 | 5.013809 | 142.4683 | 11900.12 | 12.94689 | 1.46E-03 | 0.141419 | 40.25221 |
|  | 1694.843 | 129.6956 | 18.85224 | 874.3109 | 11911.45 | 50.50956 | 5.47E-03 | 0.142287 | 162.9354 |
|  | 1702.445 | 154.2727 | 22.95728 | 922.9601 | 11178.08 | 67.80073 | 6.51E-03 | 0.152302 | 198.784 |
|  | 1742.831 | 1963.966 | 316.999 | 25768.4 | 10907.09 | 903.2613 | 8.29E-02 | 0.159789 | 8623.066 |
|  | 1755.987 | 85.79758 | 14.72692 | 144.7296 | 11125.11 | 45.63952 | 3.62E-03 | 0.15784 | 493.358 |
|  | 1758.84 | 485.4835 | 82.59812 | 2059.082 | 12021.18 | 223.5779 | 2.05E-02 | 0.146312 | 899.4855 |
|  | 1760.991 | 85.87749 | 15.01466 | 131.6188 | 7123.463 | 28.9682 | 3.62E-03 | 0.24721 | 407.7394 |
|  | 1787.994 | 512.8491 | 97.53974 | 5703.215 | 12787.08 | 213.7826 | 2.16E-02 | 0.139828 | 1510.401 |
|  | 1802.056 | 1276.384 | 263.329 | 20234.96 | 12114.44 | 572.4704 | 5.39E-02 | 0.148753 | 4727.855 |
|  | 1816.005 | 76.55466 | 17.09649 | 1144.631 | 14889.79 | 28.63242 | 3.23E-03 | 0.121963 | 37.52264 |
|  | 1830.055 | 168.9494 | 43.3853 | 978.0883 | 12830.76 | 71.90664 | 7.13E-03 | 0.14263 | 252.6924 |
|  | 842.5094 | 294.3607 | 86.68508 | 830.8729 | 7712.487 | 57.18855 | 1.24E-02 | 0.10924 | 377.118 |
|  | 1045.554 | 37.83251 | 11.10911 | 327.5273 | 9720.253 | 8.66103 | 1.60E-03 | 0.107564 | 18.28726 |
|  | 2211.104 | 65.77583 | 32.22399 | 707.5318 | 14951.27 | 28.76112 | 2.78E-03 | 0.147887 | 62.58971 |
|  |  |  |  |  |  |  |  |  |  |
| ID | m/z | Intens. | SN | Quality Fac. | Res. | Area | Rel. Intens. | FWHM | Chi^2 |
| 898 | 747.4224 | 270.8281 | 42.00643 | 834.0003 | 7853.657 | 40.8628 | 6.58E-02 | 9.52E-02 | 364.3012 |
|  | 765.5883 | 88.38895 | 13.36271 | 85.25469 | 11036.09 | 10.2044 | 2.15E-02 | 0.069371 | 225.3896 |
|  | 767.5814 | 53.37172 | 8.389722 | 141.7038 | 10271.02 | 5.820596 | 1.30E-02 | 7.47E-02 | 57.79813 |
|  | 774.4182 | 48.92318 | 7.76662 | 218.6772 | 8482.487 | 6.990011 | 1.19E-02 | 9.13E-02 | 25.87314 |
|  | 796.5197 | 28.42627 | 4.567357 | 56.31262 | 8810.482 | 4.275983 | 6.91E-03 | 9.04E-02 | 20.441 |
|  | 804.2672 | 52.45879 | 8.421737 | 404.3726 | 8502.662 | 8.190201 | 1.27E-02 | 9.46E-02 | 19.30198 |
|  | 812.3234 | 42.99406 | 7.132945 | 107.8066 | 10422.16 | 6.567309 | 1.04E-02 | 0.077942 | 44.92648 |
|  | 870.55 | 252.963 | 44.69489 | 1567.928 | 9219.323 | 40.62768 | 6.15E-02 | 9.44E-02 | 347.2963 |
|  | 888.5703 | 27.37031 | 5.165051 | 70.68606 | 8501.395 | 5.496345 | 6.65E-03 | 0.104521 | 28.0997 |
|  | 930.5489 | 35.50757 | 6.917273 | 179.2153 | 12147.14 | 5.230987 | 8.63E-03 | 7.66E-02 | 20.09533 |
|  | 1046.195 | 47.08544 | 9.989376 | 54.02317 | 13335.1 | 7.782086 | 1.14E-02 | 7.85E-02 | 132.9572 |
|  | 1048.194 | 31.89271 | 6.861068 | 86.52832 | 10599.56 | 5.966428 | 7.75E-03 | 9.89E-02 | 44.22665 |
|  | 1061.493 | 33.75524 | 7.210364 | 132.3006 | 7760.316 | 8.513433 | 8.20E-03 | 0.136785 | 22.72987 |
|  | 1118.636 | 17.5706 | 3.626971 | 50.31765 | 8288.005 | 5.391421 | 4.27E-03 | 0.13497 | 13.36516 |
|  | 1189.613 | 40.16803 | 7.528651 | 195.5061 | 20537.3 | 5.203087 | 9.76E-03 | 5.79E-02 | 21.30516 |
|  | 1209.639 | 4114.718 | 720.1963 | 14021.46 | 9637.139 | 1081.251 | 1 | 0.125518 | 49148.41 |
|  | 1242.781 | 125.0688 | 21.03201 | 1534.732 | 13483.66 | 29.68086 | 3.04E-02 | 9.22E-02 | 94.034 |
|  | 1275.711 | 72.32862 | 12.56668 | 181.0732 | 9430.225 | 21.66732 | 1.76E-02 | 0.135279 | 148.3712 |
|  | 1277.758 | 100.1366 | 17.50055 | 286.1351 | 13796.25 | 24.95547 | 2.43E-02 | 9.26E-02 | 162.2409 |
|  | 1302.718 | 44.04109 | 7.71984 | 175.8175 | 7880.474 | 13.8975 | 1.07E-02 | 0.16531 | 33.59835 |
|  | 1327.761 | 30.18462 | 5.454658 | 126.2196 | 8444.653 | 12.45664 | 7.34E-03 | 0.157231 | 27.74129 |
|  | 1329.686 | 31.25346 | 5.660216 | 73.8908 | 8888.838 | 10.1879 | 7.60E-03 | 0.149591 | 64.75165 |
|  | 1333.752 | 168.718 | 30.57071 | 2379.493 | 10159.7 | 53.25538 | 4.10E-02 | 0.131279 | 164.639 |
|  | 1381.76 | 573.6069 | 106.5016 | 7448.381 | 11969.41 | 158.0017 | 0.139404 | 0.115441 | 1194.868 |
|  | 1397.762 | 64.86071 | 12.00684 | 381.3107 | 9576.212 | 19.93717 | 1.58E-02 | 0.145962 | 59.38651 |
|  | 1461.849 | 91.87175 | 18.75886 | 1261.309 | 12616.92 | 28.11592 | 2.23E-02 | 0.115864 | 50.13318 |
|  | 1483.733 | 47.43592 | 9.926774 | 583.997 | 11083.49 | 16.17339 | 1.15E-02 | 0.133869 | 23.73408 |
|  | 1489.871 | 36.22461 | 7.732296 | 252.8308 | 11613.92 | 13.35566 | 8.80E-03 | 0.128283 | 33.4135 |
|  | 1509.848 | 1003.006 | 219.0402 | 17666.07 | 12540.38 | 308.684 | 0.243761 | 0.120399 | 2501.406 |
|  | 1525.842 | 88.96191 | 19.76966 | 1078.975 | 12517.82 | 27.23613 | 2.16E-02 | 0.121894 | 71.80284 |
|  | 1537.835 | 41.7915 | 9.451003 | 575.0418 | 15557.05 | 12.51868 | 1.02E-02 | 9.89E-02 | 18.6992 |
|  | 1814.829 | 19.36509 | 10.03394 | 537.6754 | 10622.98 | 8.380787 | 4.71E-03 | 0.17084 | 5.656934 |
|  | 1941.976 | 90.71919 | 52.2337 | 7414.449 | 15615.55 | 35.38282 | 2.20E-02 | 0.124362 | 39.81594 |
|  | 1969.966 | 25.8191 | 14.60475 | 533.1481 | 18255.78 | 8.718017 | 6.27E-03 | 0.107909 | 13.61861 |
|  | 1990.952 | 19.5371 | 12.08477 | 246.4801 | 11138.72 | 9.580982 | 4.75E-03 | 0.178741 | 14.45333 |
|  | 2038.938 | 404.4937 | 277.0861 | 8732.716 | 17657.51 | 152.8426 | 9.83E-02 | 0.115471 | 1425.09 |
|  | 2054.926 | 16.29234 | 11.70925 | 353.4779 | 20487.55 | 6.694516 | 3.96E-03 | 0.100301 | 7.457221 |
|  | 2168.118 | 73.58171 | 62.36723 | 3708.116 | 18776.87 | 26.55276 | 1.79E-02 | 0.115467 | 71.88088 |
|  | 2184.122 | 7.957953 | 6.848419 | 110.5695 | 18139.47 | 3.290941 | 1.93E-03 | 0.120407 | 4.263268 |
|  | 2696.388 | 31.62516 | 85.46299 | 2160.512 | 22389.13 | 14.15451 | 7.69E-03 | 0.120433 | 42.87058 |
|  | 2993.552 | 27.9491 | 152.9182 | 292.9075 | 26204 | 17.05908 | 6.79E-03 | 0.11424 | 49.44245 |
|  |  |  |  |  |  |  |  |  |  |
| ID | m/z | Intens. | SN | Quality Fac. | Res. | Area | Rel. Intens. | FWHM | Chi^2 |
| 966 | 701.1648 | 159.6472 | 10.41905 | 323.4645 | 6434.826 | 32.57187 | 1.26E-02 | 0.108964 | 250.8416 |
|  | 703.1803 | 78.63044 | 5.115727 | 117.5965 | 6583.94 | 13.98464 | 6.23E-03 | 0.106802 | 127.6926 |
|  | 704.1743 | 62.80053 | 4.121316 | 99.81756 | 5367.8 | 14.589 | 4.97E-03 | 0.131185 | 123.3656 |
|  | 720.1715 | 2293.696 | 148.8857 | 4694.523 | 5965.533 | 439.7153 | 0.1817 | 0.120722 | 11980.84 |
|  | 746.5489 | 84.42122 | 5.85233 | 105.1455 | 11759.26 | 9.608292 | 6.69E-03 | 6.35E-02 | 126.6013 |
|  | 765.5636 | 164.757 | 11.87294 | 94.72847 | 9164.312 | 22.76762 | 1.31E-02 | 8.35E-02 | 760.1796 |
|  | 767.5652 | 163.1945 | 11.54168 | 163.2747 | 9638.904 | 20.329 | 1.29E-02 | 7.96E-02 | 428.6608 |
|  | 774.4153 | 157.5627 | 11.65307 | 644.9624 | 6356.192 | 30.04195 | 1.25E-02 | 0.121836 | 117.4382 |
|  | 776.2336 | 162.5059 | 12.03537 | 419.6662 | 6247.956 | 37.41741 | 1.29E-02 | 0.124238 | 212.8329 |
|  | 780.3953 | 366.2344 | 27.50304 | 893.7151 | 8107.863 | 67.83471 | 2.90E-02 | 9.63E-02 | 471.6799 |
|  | 804.2607 | 137.292 | 10.68513 | 493.0447 | 6595.272 | 30.54512 | 1.09E-02 | 0.121945 | 133.1271 |
|  | 842.5094 | 1337.4 | 110.9974 | 2085.659 | 6566.611 | 289.0664 | 0.105945 | 0.128302 | 4654.827 |
|  | 856.522 | 124.9017 | 10.63768 | 520.6645 | 5936.704 | 31.69465 | 9.89E-03 | 0.144276 | 104.1387 |
|  | 870.5352 | 527.6546 | 46.11819 | 1566.468 | 7040.235 | 115.7049 | 4.18E-02 | 0.123651 | 714.0956 |
|  | 950.5156 | 68.85921 | 6.860572 | 306.7402 | 6755.094 | 17.01542 | 5.45E-03 | 0.140711 | 51.92685 |
|  | 979.4714 | 345.067 | 32.71071 | 2747.618 | 7160.577 | 84.32099 | 2.73E-02 | 0.136787 | 225.5285 |
|  | 988.5022 | 125.9875 | 11.79154 | 166.5811 | 8332.019 | 28.06648 | 9.98E-03 | 0.118639 | 354.8493 |
|  | 991.4804 | 88.21392 | 8.159305 | 651.1674 | 7889.067 | 22.82995 | 6.99E-03 | 0.125678 | 47.83726 |
|  | 1007.467 | 2569.002 | 247.4496 | 6419.39 | 7871.33 | 644.3138 | 0.203509 | 0.127992 | 24314.2 |
|  | 1045.542 | 103.2219 | 10.69436 | 396.6276 | 7882.189 | 27.72908 | 8.18E-03 | 0.132646 | 119.6545 |
|  | 1106.619 | 2934.328 | 318.8041 | 22704.1 | 8021.494 | 834.716 | 0.232449 | 0.137957 | 15081.87 |
|  | 1134.584 | 62.5171 | 6.80191 | 392.0586 | 9223.804 | 17.08768 | 4.95E-03 | 0.123006 | 41.2077 |
|  | 1171.631 | 6485.021 | 711.7312 | 30397.63 | 7341.243 | 2143.101 | 0.513724 | 0.159596 | 57116.39 |
|  | 1251.534 | 65.69832 | 6.187202 | 547.9345 | 6035.78 | 35.26035 | 5.20E-03 | 0.207352 | 44.64917 |
|  | 1258.554 | 12623.56 | 1216.824 | 38940.72 | 7502.089 | 4824.624 | 1 | 0.167761 | 136062 |
|  | 1274.551 | 49.7345 | 5.04681 | 207.0785 | 11075.58 | 13.03182 | 3.94E-03 | 0.115078 | 45.4761 |
|  | 1307.663 | 107.2267 | 12.18712 | 960.7191 | 8867.28 | 36.47144 | 8.49E-03 | 0.147471 | 64.82418 |
|  | 1315.479 | 308.4504 | 35.79417 | 1649.502 | 6917.462 | 164.093 | 2.44E-02 | 0.190168 | 444.7641 |
|  | 1327.699 | 71.5247 | 8.641155 | 352.5108 | 10625.92 | 21.89161 | 5.67E-03 | 0.124949 | 74.37687 |
|  | 1355.705 | 44.94223 | 5.886014 | 163.7925 | 8460.152 | 18.38594 | 3.56E-03 | 0.160246 | 69.85552 |
|  | 1358.614 | 34.63233 | 4.625674 | 165.5163 | 12035.09 | 10.57346 | 2.74E-03 | 0.112888 | 37.22526 |
|  | 1371.682 | 1280.973 | 178.5091 | 10614.07 | 9716.559 | 417.5267 | 0.101475 | 0.14117 | 5015.056 |
|  | 1513.738 | 123.1919 | 33.27388 | 2182.335 | 12504.74 | 41.45377 | 9.76E-03 | 0.121053 | 79.53295 |
|  | 1586.732 | 1118.363 | 356.6342 | 14682.76 | 10809.57 | 433.0364 | 8.86E-02 | 0.14679 | 4822.648 |
|  | 1675.776 | 62.07596 | 22.8276 | 660.111 | 12069.26 | 24.03115 | 4.92E-03 | 0.138847 | 59.80579 |
|  | 1687.807 | 38.81271 | 15.1511 | 117.013 | 12633.22 | 19.1679 | 3.07E-03 | 0.133601 | 106.926 |
|  | 1693.812 | 158.4917 | 62.74245 | 3001.753 | 10530.78 | 70.47073 | 1.26E-02 | 0.160844 | 150.4567 |
|  | 1703.784 | 237.5296 | 97.2689 | 8521.485 | 11618.76 | 93.69314 | 1.88E-02 | 0.146641 | 194.5164 |
|  | 1869.802 | 15.82933 | 13.54566 | 108.7137 | 27708.53 | 5.486855 | 1.25E-03 | 6.75E-02 | 20.00047 |
|  | 1920.983 | 52.84068 | 58.75389 | 954.0561 | 14533.59 | 21.91349 | 4.19E-03 | 0.132175 | 29.51724 |
|  |  |  |  |  |  |  |  |  |  |
| ID | m/z | Intens. | SN | Quality Fac. | Res. | Area | Rel. Intens. | FWHM | Chi^2 |
| 991 | 765.5758 | 69.47023 | 10.31545 | 67.91878 | 8541.187 | 9.590385 | 2.21E-02 | 8.96E-02 | 186.0498 |
|  | 767.5817 | 45.87473 | 6.559534 | 64.06011 | 9718.731 | 5.067911 | 1.46E-02 | 7.90E-02 | 76.80422 |
|  | 804.263 | 29.91754 | 4.527835 | 106.0771 | 9068.983 | 6.000347 | 9.51E-03 | 8.87E-02 | 18.11602 |
|  | 856.5175 | 141.5511 | 23.70928 | 1354.805 | 8878.773 | 22.4118 | 4.50E-02 | 9.65E-02 | 45.54337 |
|  | 870.5445 | 1295.518 | 218.1614 | 2294.202 | 9591.783 | 193.3036 | 0.411932 | 9.08E-02 | 7771.227 |
|  | 914.5533 | 476.1694 | 92.99537 | 2151.914 | 9982.203 | 75.85281 | 0.151406 | 9.16E-02 | 924.5006 |
|  | 942.535 | 19.8089 | 4.148821 | 51.53789 | 6580.802 | 5.252774 | 6.30E-03 | 0.143225 | 17.67141 |
|  | 1026.612 | 23.4203 | 5.230618 | 92.24019 | 8704.711 | 5.612329 | 7.45E-03 | 0.117938 | 14.84119 |
|  | 1043.637 | 62.18357 | 13.99064 | 67.33435 | 10977.6 | 11.42138 | 1.98E-02 | 9.51E-02 | 228.7034 |
|  | 1080.639 | 33.47272 | 7.65365 | 205.5374 | 8280.292 | 8.426085 | 1.06E-02 | 0.130507 | 17.98731 |
|  | 1118.695 | 45.84295 | 10.7508 | 494.9473 | 11794.6 | 8.567685 | 1.46E-02 | 9.48E-02 | 14.6859 |
|  | 1166.635 | 63.22746 | 15.80288 | 668.0161 | 15295.58 | 10.98974 | 2.01E-02 | 7.63E-02 | 20.48088 |
|  | 1236.743 | 595.1645 | 131.8936 | 4446.841 | 12135.57 | 127.0129 | 0.189243 | 0.101911 | 1960.594 |
|  | 1277.732 | 140.6294 | 32.15737 | 2355.734 | 13345.96 | 30.61684 | 4.47E-02 | 9.57E-02 | 64.545 |
|  | 1327.741 | 46.65863 | 10.94 | 337.0357 | 12260.84 | 12.18821 | 1.48E-02 | 0.108291 | 33.886 |
|  | 1464.798 | 23.63633 | 6.454437 | 266.7959 | 16490.05 | 7.55859 | 7.52E-03 | 8.88E-02 | 12.11636 |
|  | 1523.795 | 22.14606 | 6.797876 | 197.526 | 12093.29 | 7.798232 | 7.04E-03 | 0.126003 | 14.54721 |
|  | 1552.919 | 27.67962 | 8.616235 | 304.6339 | 11077.1 | 10.06694 | 8.80E-03 | 0.140192 | 15.6649 |
|  | 1583.738 | 32.25761 | 9.794489 | 329.9572 | 10200.78 | 14.63536 | 1.03E-02 | 0.155257 | 14.35826 |
|  | 1599.761 | 1230.806 | 366.4946 | 11528 | 13686.68 | 388.2438 | 0.391356 | 0.116885 | 7265.887 |
|  | 1614.889 | 112.8708 | 33.26476 | 2425.396 | 18525.34 | 29.38073 | 3.59E-02 | 8.72E-02 | 79.80884 |
|  | 1708.844 | 33.8889 | 14.04337 | 515.7701 | 12656.44 | 12.15831 | 0.010776 | 0.135018 | 15.79346 |
|  | 1769.939 | 1398.631 | 477.9677 | 13319.53 | 15104.08 | 481.236 | 0.444719 | 0.117183 | 9756.733 |
|  | 1790.935 | 1038.234 | 356.064 | 11022.53 | 15733.39 | 360.669 | 0.330124 | 0.11383 | 6509.209 |
|  | 1818.937 | 22.11966 | 8.45374 | 519.2948 | 18723.68 | 6.659694 | 7.03E-03 | 9.71E-02 | 6.395482 |
|  | 1930.008 | 519.0192 | 300.9674 | 5498.955 | 15697.41 | 195.1227 | 0.165031 | 0.122951 | 2075.161 |
|  | 1946.009 | 35.25666 | 20.80203 | 491.3968 | 15171.62 | 13.89876 | 1.12E-02 | 0.128266 | 21.53426 |
|  | 2447.261 | 30.36866 | 56.27381 | 515.5809 | 22062.64 | 11.15464 | 9.66E-03 | 0.110923 | 24.76015 |
|  | 2709.383 | 769.6035 | 1091.335 | 2051.176 | 19646.22 | 384.0788 | 0.244709 | 0.137909 | 9879.877 |
|  | 2726.414 | 948.8138 | 1131.898 | 866.038 | 19221.08 | 481.4348 | 0.301692 | 0.141845 | 19585.36 |
|  |  |  |  |  |  |  |  |  |  |
|  |  |  |  |  |  |  |  |  |  |
|  |  |  |  |  |  |  |  |  |  |
|  |  |  |  |  |  |  |  |  |  |
|  |  |  |  |  |  |  |  |  |  |
| ID | m/z | Intens. | SN | Quality Fac. | Res. | Area | Rel. Intens. | FWHM | Chi^2 |
| 1011 | 804.253 | 45.68144 | 7.887055 | 275.9414 | 7277.707 | 9.712951 | 1.36E-02 | 0.110509 | 21.53549 |
|  | 821.4517 | 109.146 | 18.79915 | 1867.637 | 7872.661 | 18.91233 | 3.26E-02 | 0.104342 | 43.46583 |
|  | 838.4312 | 40.07991 | 7.027047 | 210.6239 | 10678.26 | 5.359801 | 1.20E-02 | 7.85E-02 | 23.3168 |
|  | 856.5336 | 130.8819 | 23.72721 | 508.7036 | 8664.542 | 22.52149 | 3.91E-02 | 9.89E-02 | 122.2232 |
|  | 870.5466 | 1178.357 | 222.7404 | 4365.588 | 9702.128 | 188.5408 | 0.351853 | 8.97E-02 | 5853.464 |
|  | 995.5844 | 69.12792 | 15.08159 | 1236.771 | 9521.168 | 13.91628 | 0.020641 | 0.104565 | 28.67538 |
|  | 1191.7 | 94.24444 | 19.4281 | 1312.025 | 11321.02 | 22.35901 | 2.81E-02 | 0.105264 | 63.20554 |
|  | 1197.641 | 25.77153 | 5.240003 | 146.4947 | 9009.255 | 8.460439 | 7.70E-03 | 0.132935 | 15.0829 |
|  | 1219.713 | 92.35482 | 19.22623 | 1944.582 | 11512.78 | 21.87472 | 2.76E-02 | 0.105944 | 38.34461 |
|  | 1277.74 | 33.95502 | 6.543175 | 159.7561 | 8460.511 | 10.96768 | 1.01E-02 | 0.151024 | 30.15999 |
|  | 1279.645 | 23.65051 | 4.589701 | 129.5783 | 7277.92 | 11.03054 | 7.06E-03 | 0.175826 | 19.38885 |
|  | 1285.661 | 25.11854 | 4.694491 | 135.3939 | 11137.92 | 7.453749 | 7.50E-03 | 0.115431 | 19.51227 |
|  | 1327.754 | 36.1608 | 7.148913 | 395.6885 | 8095.597 | 13.86467 | 1.08E-02 | 0.164009 | 15.76476 |
|  | 1333.686 | 73.35017 | 14.69172 | 434.9559 | 13513.75 | 21.13141 | 2.19E-02 | 9.87E-02 | 80.24716 |
|  | 1391.739 | 50.00969 | 11.62816 | 1128.882 | 11838.45 | 13.38488 | 1.49E-02 | 0.117561 | 16.26562 |
|  | 1422.707 | 25.61678 | 6.187617 | 298.7224 | 10510.86 | 6.633725 | 7.65E-03 | 0.135356 | 6.529923 |
|  | 1519.831 | 25.36374 | 7.706709 | 255.744 | 11858.73 | 8.08539 | 7.57E-03 | 0.128161 | 13.33623 |
|  | 1613.761 | 2019.781 | 472.7355 | 15774.02 | 13032.59 | 659.9857 | 0.603099 | 0.123825 | 14470.37 |
|  | 1635.843 | 26.18377 | 6.401126 | 157.5508 | 15660.27 | 9.4574 | 7.82E-03 | 0.104458 | 25.60842 |
|  | 1644.862 | 28.15394 | 7.061255 | 206.9045 | 10971.84 | 11.66915 | 8.41E-03 | 0.149917 | 23.59717 |
|  | 1645.877 | 35.14688 | 8.739757 | 591.7761 | 14353.56 | 12.68137 | 0.010495 | 0.114667 | 25.08641 |
|  | 1656.787 | 57.81511 | 14.8201 | 679.495 | 16119.74 | 19.22819 | 0.017263 | 0.10278 | 85.44496 |
|  | 1689.784 | 63.3494 | 17.44017 | 60.60063 | 12554.87 | 24.33203 | 1.89E-02 | 0.134592 | 1573.735 |
|  | 1692.875 | 515.0442 | 141.8662 | 5058.325 | 14016.45 | 171.4854 | 0.15379 | 0.120778 | 2357.898 |
|  | 1698.833 | 133.5721 | 37.73641 | 6599.645 | 15332.65 | 44.28196 | 3.99E-02 | 0.110798 | 94.46202 |
|  | 1708.871 | 69.66265 | 20.31491 | 1084.875 | 15998.66 | 22.06809 | 2.08E-02 | 0.106813 | 29.94495 |
|  | 1741.961 | 91.78511 | 29.98562 | 785.9782 | 14038.99 | 32.11633 | 2.74E-02 | 0.12408 | 78.28155 |
|  | 1771.94 | 26.71222 | 10.12919 | 560.7376 | 13797.73 | 9.868733 | 7.98E-03 | 0.128423 | 9.384851 |
|  | 1977.055 | 317.443 | 180.7832 | 4753.528 | 17066.67 | 118.2728 | 9.48E-02 | 0.115843 | 1177.569 |
|  | 2066.906 | 12.33682 | 8.535475 | 341.1935 | 18578.29 | 5.046629 | 3.68E-03 | 0.111254 | 4.449796 |
|  | 2088.049 | 18.92836 | 14.56235 | 422.2913 | 13992.98 | 9.338354 | 5.65E-03 | 0.149221 | 9.312072 |
|  | 2225.099 | 14.76396 | 11.69532 | 320.956 | 25314.7 | 4.261893 | 4.41E-03 | 8.79E-02 | 6.542315 |
|  | 2233.086 | 9.448059 | 7.760591 | 236.2542 | 14602.27 | 4.694079 | 2.82E-03 | 0.152927 | 4.059112 |
|  | 2707.421 | 7.605149 | 21.55005 | 226.484 | 14757.33 | 4.615914 | 2.27E-03 | 0.183463 | 3.30496 |
|  |  |  |  |  |  |  |  |  |  |
|  |  |  |  |  |  |  |  |  |  |
|  |  |  |  |  |  |  |  |  |  |
|  |  |  |  |  |  |  |  |  |  |
|  |  |  |  |  |  |  |  |  |  |
| ID | m/z | Intens. | SN | Quality Fac. | Res. | Area | Rel. Intens. | FWHM | Chi^2 |
| 1095 | 702.1915 | 40.31984 | 7.107672 | 92.43397 | 4044.734 | 10.15049 | 4.02E-03 | 0.173606 | 45.72154 |
|  | 720.6111 | 70.4275 | 12.39182 | 154.4813 | 10144.96 | 10.00644 | 7.02E-03 | 7.10E-02 | 93.7766 |
|  | 730.1788 | 25.31053 | 4.385576 | 75.21585 | 6786.747 | 5.409027 | 2.52E-03 | 0.107589 | 17.39935 |
|  | 756.9165 | 134.6549 | 21.06263 | 196.7292 | 12108.87 | 13.71911 | 1.34E-02 | 6.25E-02 | 218.9045 |
|  | 758.9057 | 34.60402 | 5.442345 | 73.62144 | 12802.23 | 3.155842 | 3.45E-03 | 5.93E-02 | 33.00168 |
|  | 765.5643 | 587.3978 | 91.30231 | 129.3328 | 9130.612 | 81.88644 | 5.85E-02 | 8.38E-02 | 7804.743 |
|  | 767.5652 | 466.0246 | 71.53336 | 208.8209 | 8757.759 | 68.35658 | 4.64E-02 | 8.76E-02 | 3583.914 |
|  | 774.4201 | 56.05616 | 8.443975 | 389.5956 | 6761.617 | 12.29227 | 5.58E-03 | 0.114532 | 27.60222 |
|  | 778.358 | 289.3949 | 43.63838 | 452.2116 | 7219.732 | 50.47282 | 2.88E-02 | 0.10781 | 657.415 |
|  | 794.3985 | 269.8304 | 41.85418 | 644.1974 | 9320.701 | 44.43358 | 2.69E-02 | 8.52E-02 | 360.5011 |
|  | 812.3205 | 360.1945 | 56.23666 | 122.4117 | 9750.671 | 51.53143 | 3.59E-02 | 8.33E-02 | 3244.166 |
|  | 814.3222 | 161.9327 | 25.59866 | 128.3367 | 10248.18 | 21.69217 | 1.61E-02 | 7.95E-02 | 687.1196 |
|  | 838.4524 | 49.29269 | 7.627152 | 291.5061 | 10263.29 | 8.473193 | 4.91E-03 | 8.17E-02 | 24.89232 |
|  | 856.5113 | 82.81658 | 11.64825 | 514.3883 | 6758.878 | 18.08169 | 8.25E-03 | 0.126724 | 50.6724 |
|  | 860.5041 | 390.3563 | 54.17899 | 707.3229 | 8271.797 | 73.84776 | 3.89E-02 | 0.104029 | 767.5565 |
|  | 868.4625 | 364.7934 | 48.68554 | 329.3167 | 7970.134 | 68.95803 | 3.63E-02 | 0.108965 | 1816.287 |
|  | 870.5212 | 1072.2 | 142.5961 | 2342.929 | 6746.206 | 238.7606 | 0.106804 | 0.129039 | 7290.464 |
|  | 872.5035 | 2065.79 | 271.1249 | 9644.508 | 7261.144 | 434.7048 | 0.205777 | 0.120161 | 7257.146 |
|  | 876.4875 | 49.61315 | 6.364217 | 114.6857 | 10210.97 | 7.910368 | 4.94E-03 | 8.58E-02 | 61.68058 |
|  | 892.487 | 403.712 | 55.56332 | 898.4767 | 8866.358 | 76.30636 | 4.02E-02 | 0.10066 | 841.8944 |
|  | 927.4452 | 66.42918 | 11.94308 | 522.7648 | 9073.942 | 14.31375 | 6.62E-03 | 0.10221 | 32.73072 |
|  | 950.5147 | 31.68899 | 6.242242 | 200.9054 | 6917.531 | 8.777603 | 3.16E-03 | 0.137407 | 19.05845 |
|  | 956.1985 | 26.3562 | 4.953493 | 54.72112 | 16589.09 | 3.117728 | 2.63E-03 | 5.76E-02 | 28.73781 |
|  | 991.4514 | 24.9403 | 5.13754 | 98.63595 | 7630.53 | 9.872465 | 2.48E-03 | 0.129932 | 18.48898 |
|  | 1032.533 | 1390.21 | 240.8571 | 11723.51 | 8229.593 | 328.2861 | 0.138481 | 0.125466 | 3141.606 |
|  | 1042.556 | 43.98737 | 7.352711 | 91.61558 | 9611.371 | 10.64859 | 4.38E-03 | 0.108471 | 87.18963 |
|  | 1046.118 | 319.4417 | 52.55782 | 69.14534 | 12969.81 | 52.94862 | 3.18E-02 | 8.07E-02 | 5116.809 |
|  | 1048.123 | 225.0633 | 36.88857 | 92.76683 | 12200.61 | 37.11022 | 2.24E-02 | 8.59E-02 | 2069.975 |
|  | 1054.538 | 30.44227 | 4.961401 | 131.2506 | 9825.387 | 8.522271 | 3.03E-03 | 0.107328 | 27.53501 |
|  | 1089.604 | 110.8044 | 20.2344 | 755.2925 | 8999.874 | 30.14016 | 1.10E-02 | 0.121069 | 106.0884 |
|  | 1137.579 | 33.23282 | 6.623334 | 195.7053 | 7624.358 | 10.0781 | 3.31E-03 | 0.149203 | 19.13481 |
|  | 1138.609 | 25.51432 | 4.990028 | 77.98715 | 13463.91 | 3.030437 | 2.54E-03 | 8.46E-02 | 22.35671 |
|  | 1150.616 | 178.2282 | 35.27641 | 314.8148 | 8659.426 | 47.93106 | 1.78E-02 | 0.132874 | 514.5329 |
|  | 1206.663 | 40.35765 | 5.883487 | 72.00574 | 9052.457 | 11.25748 | 4.02E-03 | 0.133297 | 63.33424 |
|  | 1226.617 | 29.53847 | 3.739892 | 147.4052 | 6192.114 | 13.40781 | 2.94E-03 | 0.198093 | 21.14636 |
|  | 1241.617 | 10038.99 | 1206.111 | 79901.22 | 7383.47 | 3725.641 | 1 | 0.168162 | 61702.93 |
|  | 1253.631 | 77.77647 | 10.0968 | 106.6593 | 9327.365 | 23.48625 | 7.75E-03 | 0.134404 | 265.1279 |
|  | 1257.671 | 177.7195 | 23.87735 | 1178.247 | 10849.53 | 54.05008 | 1.77E-02 | 0.115919 | 179.5257 |
|  | 1281.916 | 51.7351 | 8.383069 | 69.16921 | 19511.34 | 7.226308 | 5.15E-03 | 6.57E-02 | 146.5736 |
|  | 1302.674 | 24.94926 | 4.943191 | 142.248 | 6281.168 | 11.43945 | 2.49E-03 | 0.207394 | 16.0535 |
|  | 1327.715 | 78.71702 | 15.83279 | 581.7422 | 8194.805 | 26.92313 | 7.84E-03 | 0.162019 | 55.20143 |
|  | 1333.728 | 48.65407 | 9.781125 | 104.6891 | 11565.49 | 18.18034 | 4.85E-03 | 0.11532 | 126.6578 |
|  | 1335.707 | 27.61767 | 5.551701 | 149.5827 | 8304.892 | 10.57314 | 2.75E-03 | 0.160834 | 30.64731 |
|  | 1345.606 | 232.5844 | 46.87915 | 4294.438 | 11252.3 | 71.00172 | 0.023168 | 0.119585 | 194.3262 |
|  | 1360.691 | 156.0852 | 31.3893 | 2551.438 | 9518.412 | 52.69553 | 1.55E-02 | 0.142954 | 86.46188 |
|  | 1436.785 | 24.95654 | 5.263476 | 112.4564 | 7471.355 | 11.15126 | 2.49E-03 | 0.192306 | 29.87813 |
|  | 1450.767 | 69.17397 | 13.74966 | 1310.777 | 10273.78 | 26.9336 | 6.89E-03 | 0.141211 | 22.02422 |
|  | 1484.739 | 2703.233 | 449.3891 | 25485.2 | 9106.861 | 1084.71 | 0.269273 | 0.163035 | 17365.43 |
|  | 1509.76 | 569.3395 | 83.54012 | 14599.81 | 9913.51 | 215.1578 | 5.67E-02 | 0.152293 | 686.9882 |
|  | 1554.61 | 554.0871 | 85.14459 | 8077.816 | 10607.24 | 225.3419 | 5.52E-02 | 0.146561 | 1368.343 |
|  | 1570.599 | 35.44095 | 5.535891 | 451.4718 | 11380.09 | 15.31843 | 3.53E-03 | 0.138013 | 17.96465 |
|  | 1611.741 | 4111.517 | 754.2481 | 36363.43 | 9097.306 | 1966.797 | 0.409555 | 0.177167 | 27799.52 |
|  | 1662.805 | 116.7302 | 28.42318 | 3001.275 | 12166.3 | 48.59813 | 1.16E-02 | 0.136673 | 78.02854 |
|  | 1681.868 | 25.6421 | 5.852388 | 229.0582 | 13083.39 | 10.9395 | 2.55E-03 | 0.12855 | 21.44497 |
|  | 1718.879 | 50.80637 | 10.59031 | 253.261 | 9848.958 | 25.67672 | 5.06E-03 | 0.174524 | 45.2542 |
|  | 1734.901 | 4051.501 | 829.2061 | 31010.21 | 9468.922 | 2163.659 | 0.403577 | 0.183221 | 33757.72 |
|  | 1804.799 | 18.07026 | 4.434162 | 91.45225 | 12360.7 | 8.582989 | 1.80E-03 | 0.146011 | 14.0319 |
|  | 1852.796 | 271.5085 | 61.93089 | 6746.157 | 11272.56 | 130.0913 | 0.027045 | 0.164363 | 474.2376 |
|  | 1865.892 | 381.9565 | 83.53981 | 27637.3 | 11735.16 | 190.8482 | 0.038047 | 0.159 | 317.7802 |
|  | 1868.798 | 52.32498 | 11.4462 | 495.9115 | 12221.45 | 21.47304 | 5.21E-03 | 0.152911 | 43.34317 |
|  | 1884.759 | 28.43035 | 6.003069 | 251.398 | 9576.851 | 19.1225 | 2.83E-03 | 0.196804 | 29.50629 |
|  | 1924.99 | 41.30274 | 10.11784 | 446.2264 | 9899.299 | 25.74892 | 4.11E-03 | 0.194457 | 39.77495 |
|  | 1997.009 | 24.07929 | 7.261853 | 446.7013 | 10630.88 | 14.43354 | 2.40E-03 | 0.18785 | 12.88616 |
|  | 2014.943 | 315.1308 | 97.45989 | 8148.919 | 12261.53 | 172.6961 | 3.14E-02 | 0.164331 | 607.7833 |
|  | 2025.015 | 37.94755 | 11.79961 | 880.6733 | 14830.82 | 17.92793 | 3.78E-03 | 0.136541 | 16.36304 |
|  | 2040.965 | 20.54063 | 6.474249 | 205.7168 | 12739.83 | 11.71162 | 2.05E-03 | 0.160203 | 11.7894 |
|  | 2068.014 | 121.0369 | 40.83607 | 4907.202 | 12938.57 | 61.25876 | 1.21E-02 | 0.159833 | 108.0423 |
|  | 2110.953 | 16.74951 | 7.359763 | 381.9785 | 4507.678 | 32.78544 | 1.67E-03 | 0.468302 | 19.63708 |
|  | 2230.072 | 213.953 | 74.21511 | 8973.545 | 13184.21 | 121.6548 | 2.13E-02 | 0.169147 | 306.1783 |
|  | 2233.124 | 29.10771 | 10.14022 | 234.7121 | 14582.34 | 11.628 | 2.90E-03 | 0.153139 | 33.74009 |
|  | 2358.181 | 61.21341 | 26.66633 | 1093.783 | 16601.79 | 29.20841 | 6.10E-03 | 0.142044 | 61.65643 |
|  | 2422.192 | 363.1506 | 95.25029 | 2623.721 | 14277.39 | 239.9831 | 3.62E-02 | 0.169652 | 3021.293 |
|  | 2429.577 | 198.0445 | 47.51517 | 690.9957 | 12171.51 | 154.6151 | 1.97E-02 | 0.199612 | 965.3865 |
|  | 2453.182 | 29.3946 | 5.56452 | 383.9623 | 10619.7 | 20.16993 | 2.93E-03 | 0.231003 | 30.50104 |
|  | 2470.197 | 4147.64 | 679.3104 | 2855.252 | 11952.73 | 3205.492 | 0.413153 | 0.206664 | 151028.4 |
|  | 2486.202 | 481.1513 | 70.0097 | 2723.448 | 16767.7 | 299.2131 | 4.79E-02 | 0.148273 | 4590.254 |
|  | 2494.239 | 35.25774 | 5.407615 | 757.584 | 11897.99 | 26.46005 | 3.51E-03 | 0.209635 | 24.9914 |
|  | 2510.297 | 411.0867 | 76.12958 | 1928.635 | 13616.59 | 266.5194 | 0.040949 | 0.184356 | 2337.02 |
|  | 2866.501 | 10.36001 | 34.40013 | 525.8074 | 16825.58 | 6.736517 | 1.03E-03 | 0.170366 | 6.190183 |
|  |  |  |  |  |  |  |  |  |  |
| ID | m/z | Intens. | SN | Quality Fac. | Res. | Area | Rel. Intens. | FWHM | Chi^2 |
| 1153 | 764.3979 | 78.2757 | 17.04315 | 627.9999 | 8262.758 | 12.27661 | 8.01E-03 | 9.25E-02 | 37.49395 |
|  | 804.2665 | 18.80559 | 4.201411 | 51.70848 | 9174.695 | 3.514631 | 1.92E-03 | 8.77E-02 | 17.01598 |
|  | 856.5217 | 87.90371 | 19.85624 | 568.2844 | 9265.58 | 13.42854 | 9.00E-03 | 9.24E-02 | 41.57671 |
|  | 870.5415 | 834.3349 | 197.7005 | 3020.961 | 9590.084 | 128.0374 | 8.54E-02 | 9.08E-02 | 2410.764 |
|  | 883.4402 | 123.745 | 30.32263 | 837.8966 | 10039.3 | 21.04114 | 1.27E-02 | 8.80E-02 | 102.1247 |
|  | 1085.63 | 24.61898 | 5.783176 | 137.4736 | 7416.656 | 8.360161 | 2.52E-03 | 0.146377 | 21.8249 |
|  | 1101.575 | 887.6275 | 203.9377 | 4011.428 | 12567.15 | 171.023 | 9.08E-02 | 8.77E-02 | 3901.433 |
|  | 1113.569 | 29.52198 | 6.601308 | 193.7755 | 11252.42 | 7.374432 | 3.02E-03 | 9.90E-02 | 18.35052 |
|  | 1159.601 | 2586.042 | 513.4254 | 8509.924 | 10493.6 | 601.3287 | 0.264628 | 0.110506 | 29471.56 |
|  | 1176.64 | 218.7302 | 41.13922 | 1007.796 | 11347.21 | 49.64639 | 2.24E-02 | 0.103694 | 541.4826 |
|  | 1203.63 | 119.8448 | 20.60206 | 1479.93 | 9725.542 | 31.07095 | 1.23E-02 | 0.12376 | 67.86178 |
|  | 1211.531 | 38.81635 | 6.448834 | 389.2058 | 6623.554 | 18.82933 | 3.97E-03 | 0.182913 | 24.03716 |
|  | 1233.633 | 32.66514 | 5.055437 | 154.0165 | 10000.73 | 8.788376 | 3.34E-03 | 0.123354 | 24.49351 |
|  | 1251.636 | 3745.757 | 590.5039 | 16671.78 | 10864.8 | 923.0352 | 0.383301 | 0.115201 | 34438.16 |
|  | 1267.639 | 595.5582 | 97.29239 | 4016.719 | 12140.18 | 139.8203 | 6.09E-02 | 0.104417 | 1361.142 |
|  | 1283.706 | 38.11312 | 6.281658 | 129.8542 | 14668.52 | 8.660107 | 3.90E-03 | 0.087514 | 29.19044 |
|  | 1312.749 | 73.53589 | 11.25925 | 714.9707 | 13836.93 | 17.98644 | 7.52E-03 | 9.49E-02 | 35.96672 |
|  | 1352.704 | 252.5985 | 23.44625 | 1228.873 | 12243.11 | 63.91663 | 2.58E-02 | 0.110487 | 260.6536 |
|  | 1360.483 | 94.74398 | 8.150891 | 451.2912 | 11422.8 | 35.95847 | 9.70E-03 | 0.119102 | 115.5669 |
|  | 1379.736 | 779.2514 | 57.3982 | 2718.907 | 12206.13 | 208.3087 | 7.97E-02 | 0.113036 | 2148.417 |
|  | 1385.682 | 53.13346 | 4.050513 | 573.8411 | 7495.854 | 22.06041 | 5.44E-03 | 0.18486 | 28.64956 |
|  | 1395.727 | 94.40263 | 7.718756 | 404.9903 | 12475.78 | 23.82343 | 9.66E-03 | 0.111875 | 101.5715 |
|  | 1400.718 | 9772.368 | 835.7123 | 4129.755 | 9681.706 | 3247.656 | 1 | 0.144677 | 551808.3 |
|  | 1401.752 | 6807.556 | 584.1105 | 3509.704 | 11140.6 | 2111.316 | 0.696613 | 0.125824 | 440936.4 |
|  | 1416.707 | 1215.027 | 123.3462 | 14952.32 | 13470.67 | 306.3828 | 0.124333 | 0.10517 | 3359.553 |
|  | 1429.731 | 131.4044 | 15.20091 | 810.0848 | 14842.97 | 31.61011 | 1.34E-02 | 9.63E-02 | 109.7337 |
|  | 1457.743 | 34.02767 | 6.077916 | 161.6752 | 16743.74 | 8.444545 | 3.48E-03 | 0.087062 | 29.00219 |
|  | 1473.749 | 37.67119 | 6.799637 | 186.7016 | 12323.09 | 10.36885 | 3.85E-03 | 0.119592 | 28.10109 |
|  | 1518.749 | 468.8247 | 91.46242 | 6305.637 | 14972.27 | 128.2353 | 4.80E-02 | 0.101437 | 1185.665 |
|  | 1534.759 | 50.97969 | 10.12313 | 845.8943 | 9358.234 | 19.97982 | 5.22E-03 | 0.164001 | 18.80444 |
|  | 1554.837 | 75.03343 | 14.81966 | 172.0416 | 14939.28 | 23.64469 | 7.68E-03 | 0.104077 | 96.87084 |
|  | 1583.875 | 28.70833 | 5.458382 | 172.6681 | 16799.95 | 7.588303 | 2.94E-03 | 9.43E-02 | 23.91176 |
|  | 1610.888 | 28.33278 | 5.422443 | 201.1351 | 12916.79 | 8.922166 | 2.90E-03 | 0.124713 | 22.19033 |
|  | 1636.863 | 249.6696 | 50.6338 | 2425.634 | 15196.11 | 75.00146 | 0.025549 | 0.107716 | 794.2566 |
|  | 1643.891 | 387.9462 | 80.98779 | 4161.156 | 14153.4 | 107.7891 | 0.039698 | 0.116148 | 925.086 |
|  | 1695.781 | 42.18738 | 10.59363 | 765.3306 | 21249.12 | 10.73441 | 4.32E-03 | 7.98E-02 | 13.99029 |
|  | 1737.852 | 78.84943 | 23.70925 | 2139.756 | 12948.4 | 29.0114 | 0.008069 | 0.134214 | 35.37636 |
|  | 1764.944 | 19.94817 | 6.656687 | 155.0856 | 12187.97 | 8.3183 | 2.04E-03 | 0.14481 | 14.2303 |
|  | 2006.012 | 99.67065 | 46.50787 | 3954.8 | 17139.47 | 37.26335 | 1.02E-02 | 0.117041 | 87.93944 |
|  | 2013.528 | 150.3634 | 69.45253 | 1003.219 | 14046.02 | 82.60075 | 1.54E-02 | 0.143352 | 243.9343 |
|  | 2054.015 | 625.6389 | 255.4518 | 9630.704 | 17770.53 | 238.4251 | 6.40E-02 | 0.115585 | 3003.676 |
|  | 2070.015 | 484.1373 | 216.8118 | 6975.222 | 18374.21 | 175.2532 | 4.95E-02 | 0.112659 | 2480.591 |
|  | 2178.117 | 15.93071 | 11.03096 | 88.24917 | 24708.51 | 4.708867 | 1.63E-03 | 8.82E-02 | 8.680131 |
|  | 2763.468 | 12.56756 | 29.04502 | 500.1083 | 19470.37 | 6.011002 | 1.29E-03 | 0.141932 | 9.050202 |
|  |  |  |  |  |  |  |  |  |  |
| ID | m/z | Intens. | SN | Quality Fac. | Res. | Area | Rel. Intens. | FWHM | Chi^2 |
| 1186 | 765.5732 | 91.91248 | 11.94032 | 77.71777 | 11100.9 | 10.42135 | 1.53E-02 | 6.90E-02 | 278.8497 |
|  | 767.5703 | 89.64262 | 11.68365 | 123.0418 | 12053.46 | 9.673083 | 1.50E-02 | 6.37E-02 | 174.4945 |
|  | 856.5273 | 194.1198 | 27.09898 | 791.4377 | 9665.424 | 27.54164 | 3.24E-02 | 8.86E-02 | 141.3503 |
|  | 870.5441 | 1749.578 | 254.8021 | 5255.614 | 8933.068 | 287.0698 | 0.291978 | 9.75E-02 | 10819.84 |
|  | 1057.575 | 32.22838 | 5.238161 | 111.8364 | 6891.927 | 11.16923 | 5.38E-03 | 0.153451 | 36.8697 |
|  | 1091.603 | 120.7173 | 19.46521 | 1512.316 | 9978.576 | 25.8951 | 2.01E-02 | 0.109395 | 66.03823 |
|  | 1158.604 | 188.5341 | 31.19042 | 2776.946 | 9561.786 | 46.86974 | 3.15E-02 | 0.12117 | 293.112 |
|  | 1195.618 | 40.31204 | 6.501797 | 82.49343 | 11913.27 | 9.290465 | 6.73E-03 | 0.10036 | 60.51986 |
|  | 1245.631 | 40.39709 | 6.145105 | 214.7871 | 10477.13 | 11.00256 | 6.74E-03 | 0.11889 | 25.13996 |
|  | 1277.74 | 743.522 | 110.1169 | 7455.798 | 11716.4 | 168.4639 | 0.124082 | 0.109056 | 1996.166 |
|  | 1284.702 | 321.4973 | 46.94707 | 6373.558 | 11355.51 | 82.24925 | 5.37E-02 | 0.113135 | 360.3979 |
|  | 1327.749 | 30.87706 | 4.451866 | 161.8983 | 9751.661 | 8.787168 | 5.15E-03 | 0.136156 | 19.16527 |
|  | 1347.735 | 92.88367 | 14.01575 | 616.0164 | 14243.96 | 19.85418 | 0.015501 | 9.46E-02 | 60.46166 |
|  | 1372.73 | 543.6826 | 83.93394 | 4042.584 | 13848.29 | 130.1353 | 9.07E-02 | 0.099126 | 2311.646 |
|  | 1373.728 | 259.6704 | 40.36451 | 195.6338 | 13178.76 | 76.01937 | 4.33E-02 | 0.104238 | 1960.862 |
|  | 1412.797 | 42.17349 | 6.970265 | 546.4894 | 16437.95 | 8.853084 | 7.04E-03 | 0.085947 | 10.3946 |
|  | 1523.827 | 38.5752 | 7.427745 | 334.9328 | 12417.76 | 12.34122 | 6.44E-03 | 0.122714 | 24.30804 |
|  | 1568.887 | 2029.736 | 416.1431 | 12748.07 | 13470.4 | 615.7707 | 0.338732 | 0.116469 | 17526.61 |
|  | 1584.945 | 34.13265 | 7.492147 | 312.1144 | 12422.38 | 12.73126 | 5.70E-03 | 0.127588 | 23.58352 |
|  | 1716.831 | 29.90944 | 8.765613 | 637.0192 | 14926.69 | 9.457436 | 0.004991 | 0.115018 | 8.962717 |
|  | 1841.939 | 24.225 | 7.907605 | 367.5632 | 14420.02 | 9.7619 | 4.04E-03 | 0.127735 | 12.11043 |
|  | 1856.962 | 27.34388 | 8.607692 | 184.8701 | 13526.92 | 11.41573 | 4.56E-03 | 0.137279 | 27.53138 |
|  | 1877.051 | 45.15155 | 14.70114 | 439.8855 | 15792.75 | 18.1594 | 7.54E-03 | 0.118855 | 39.12459 |
|  | 1901.952 | 16.02391 | 5.72212 | 115.2388 | 18012.01 | 6.77383 | 2.67E-03 | 0.105594 | 8.699596 |
|  | 1932.042 | 52.43744 | 22.14147 | 915.675 | 14955.61 | 20.48992 | 8.75E-03 | 0.129185 | 26.20745 |
|  | 1993.976 | 29.49204 | 15.93088 | 174.5618 | 16175.36 | 11.47433 | 4.92E-03 | 0.123272 | 41.47129 |
|  | 1996.96 | 27.74113 | 14.4447 | 521.9442 | 20961.67 | 10.00583 | 4.63E-03 | 9.53E-02 | 17.49306 |
|  | 2192.047 | 34.80052 | 18.84287 | 631.297 | 16598.92 | 15.79985 | 5.81E-03 | 0.13206 | 18.99733 |
|  | 2233.06 | 18.60002 | 9.729157 | 318.073 | 20408.35 | 7.449731 | 3.10E-03 | 0.109419 | 4.382039 |
|  | 2281.198 | 87.75108 | 58.48618 | 6696.443 | 22391.2 | 31.3119 | 1.46E-02 | 0.101879 | 64.36043 |
|  | 2689.361 | 17.4871 | 34.68156 | 419.6206 | 21656.08 | 8.976975 | 2.92E-03 | 0.124185 | 9.905803 |
|  | 2705.182 | 13.3505 | 25.47776 | 303.3703 | 21675.27 | 7.875004 | 2.23E-03 | 0.124805 | 8.103336 |
|  | 2707.423 | 12.75936 | 25.15972 | 246.5188 | 19819.94 | 4.67527 | 2.13E-03 | 0.136601 | 7.726013 |
|  | 2746.386 | 24.85572 | 46.24007 | 1175.256 | 27318.11 | 9.822592 | 4.15E-03 | 0.100534 | 15.74821 |
|  |  |  |  |  |  |  |  |  |  |
| ID | m/z | Intens. | SN | Quality Fac. | Res. | Area | Rel. Intens. | FWHM | Chi^2 |
| 1206 | 746.5748 | 225.2563 | 20.26562 | 643.9196 | 6885.519 | 41.42725 | 1.48E-02 | 0.108427 | 232.8966 |
|  | 748.5663 | 88.75992 | 7.966588 | 242.4079 | 6163.883 | 17.01246 | 5.83E-03 | 0.121444 | 98.57446 |
|  | 765.571 | 203.5446 | 17.90366 | 145.9477 | 8117.291 | 31.08262 | 1.34E-02 | 9.43E-02 | 801.4618 |
|  | 767.5666 | 187.4349 | 16.4884 | 200.3931 | 9316.648 | 25.62206 | 1.23E-02 | 0.082387 | 527.145 |
|  | 779.3726 | 5146.289 | 451.6362 | 7903.48 | 6713.624 | 1033.888 | 0.337832 | 0.116088 | 57378 |
|  | 788.477 | 59.28348 | 5.316427 | 260.4239 | 5368.87 | 19.6909 | 3.89E-03 | 0.146861 | 46.46996 |
|  | 798.4511 | 55.39724 | 5.07763 | 100.7371 | 7546.036 | 9.562283 | 3.64E-03 | 0.105811 | 56.1939 |
|  | 812.3152 | 104.9998 | 9.895266 | 66.94284 | 13831.44 | 11.65225 | 6.89E-03 | 5.87E-02 | 410.8939 |
|  | 828.4771 | 784.959 | 76.5978 | 1628.324 | 7462.288 | 135.8398 | 5.15E-02 | 0.111022 | 1205.34 |
|  | 844.474 | 273.1826 | 26.90935 | 796.2893 | 7741.717 | 49.05024 | 0.017933 | 0.109081 | 290.8727 |
|  | 856.5152 | 97.96669 | 9.937659 | 583.5702 | 7975.278 | 18.21 | 6.43E-03 | 0.107396 | 52.32626 |
|  | 870.5469 | 494.2011 | 51.02639 | 160.4228 | 7946.884 | 99.30029 | 3.24E-02 | 0.109546 | 5943.293 |
|  | 872.5215 | 1557.388 | 160.637 | 1153.754 | 8435.561 | 289.5764 | 0.102236 | 0.103434 | 8729.276 |
|  | 917.4485 | 1201.398 | 125.4099 | 3167.503 | 7797.881 | 258.1685 | 7.89E-02 | 0.117654 | 5498.043 |
|  | 933.4482 | 124.8392 | 13.01646 | 503.8263 | 8169.052 | 28.57518 | 8.20E-03 | 0.114266 | 119.7269 |
|  | 949.4403 | 1141.321 | 121.1836 | 1928.942 | 8805.804 | 237.6116 | 7.49E-02 | 0.10782 | 6090.719 |
|  | 1013.575 | 84.56196 | 8.75884 | 603.9457 | 6882.952 | 23.60303 | 5.55E-03 | 0.147259 | 53.47568 |
|  | 1028.604 | 6827.747 | 658.1214 | 20693.61 | 7201.559 | 1844.097 | 0.448212 | 0.142831 | 69243.16 |
|  | 1032.594 | 151.8931 | 14.25785 | 546.9839 | 8237.602 | 36.82079 | 9.97E-03 | 0.125351 | 160.6084 |
|  | 1048.125 | 101.5802 | 8.471961 | 66.58231 | 13260.71 | 14.82344 | 6.67E-03 | 7.90E-02 | 510.2576 |
|  | 1059.55 | 63.27931 | 5.49507 | 73.14988 | 8611.204 | 12.41654 | 4.15E-03 | 0.123043 | 125.4628 |
|  | 1069.56 | 59.01302 | 5.448088 | 146.2324 | 11700.41 | 10.6605 | 3.87E-03 | 9.14E-02 | 62.61621 |
|  | 1077.543 | 75.73882 | 7.318929 | 209.4263 | 10111.25 | 16.65565 | 4.97E-03 | 0.106569 | 94.78742 |
|  | 1081.551 | 251.764 | 24.55074 | 794.8144 | 9411.7 | 59.13111 | 1.65E-02 | 0.114916 | 320.1126 |
|  | 1129.606 | 212.159 | 25.63182 | 1329.353 | 9353.602 | 49.63597 | 0.013927 | 0.120767 | 140.3285 |
|  | 1198.713 | 871.3013 | 113.4903 | 2339.067 | 10331.75 | 209.471 | 0.057197 | 0.116022 | 3033.188 |
|  | 1241.617 | 64.2225 | 8.276699 | 361.3279 | 10594.37 | 13.90612 | 4.22E-03 | 0.117196 | 43.98092 |
|  | 1302.719 | 112.1518 | 13.5776 | 688.0419 | 10604.43 | 30.975 | 7.36E-03 | 0.122847 | 93.80602 |
|  | 1316.184 | 49.26396 | 4.613825 | 415.9975 | 9560.538 | 18.01618 | 3.23E-03 | 0.137668 | 29.14487 |
|  | 1335.682 | 194.355 | 13.55901 | 956.2332 | 12415.21 | 48.01423 | 1.28E-02 | 0.107584 | 180.8937 |
|  | 1359.662 | 4029.327 | 213.2527 | 18261.88 | 10020.54 | 1334.404 | 0.264508 | 0.135687 | 37652.51 |
|  | 1372.686 | 190.0283 | 9.176488 | 768.229 | 11467.34 | 51.12568 | 1.25E-02 | 0.119704 | 225.0979 |
|  | 1384.736 | 1343.961 | 71.49882 | 10267.83 | 10560.52 | 434.7783 | 8.82E-02 | 0.131124 | 3303.101 |
|  | 1388.68 | 86.31252 | 4.736196 | 271.3425 | 9926.583 | 25.19448 | 5.67E-03 | 0.139895 | 141.6954 |
|  | 1398.707 | 69.13364 | 4.277678 | 170.5885 | 9489.454 | 23.98552 | 4.54E-03 | 0.147396 | 159.6338 |
|  | 1400.705 | 82.9821 | 5.214988 | 196.1236 | 12145.31 | 22.32847 | 5.45E-03 | 0.115329 | 173.5999 |
|  | 1404.703 | 171.6665 | 11.19659 | 332.5125 | 12634.74 | 47.63111 | 1.13E-02 | 0.111178 | 413.4114 |
|  | 1409.705 | 104.2554 | 7.295814 | 319.7102 | 10803.79 | 29.66285 | 6.84E-03 | 0.130482 | 172.8379 |
|  | 1412.733 | 326.4887 | 23.72009 | 1799.094 | 11441.1 | 100.2855 | 2.14E-02 | 0.123479 | 328.8534 |
|  | 1415.679 | 81.51961 | 6.013666 | 169.534 | 9239.199 | 29.51558 | 5.35E-03 | 0.153225 | 243.3209 |
|  | 1416.697 | 277.1745 | 20.7344 | 984.7435 | 11221.86 | 82.39746 | 1.82E-02 | 0.126244 | 414.3319 |
|  | 1431.756 | 79.52852 | 7.560152 | 174.7759 | 14813.66 | 20.20655 | 5.22E-03 | 9.67E-02 | 175.5877 |
|  | 1513.757 | 88.40259 | 15.68381 | 532.2156 | 12304.42 | 28.34096 | 5.80E-03 | 0.123025 | 86.54566 |
|  | 1575.819 | 40.79934 | 8.519525 | 508.2649 | 11990.12 | 15.87541 | 2.68E-03 | 0.131426 | 21.23815 |
|  | 1625.984 | 58.59537 | 11.29213 | 477.7841 | 9912.638 | 25.40266 | 3.85E-03 | 0.164031 | 53.51951 |
|  | 1662.491 | 31.30672 | 5.633482 | 236.4116 | 25090.74 | 8.837408 | 2.06E-03 | 6.63E-02 | 23.97889 |
|  | 1702.874 | 737.4438 | 110.5151 | 16321.96 | 12760.53 | 293.3565 | 4.84E-02 | 0.133448 | 1638.972 |
|  | 1718.855 | 107.5319 | 15.0791 | 595.3556 | 16632.2 | 38.39831 | 7.06E-03 | 0.103345 | 134.3906 |
|  | 1739.818 | 2729.849 | 352.5876 | 39099.88 | 12338.76 | 1168.95 | 0.179203 | 0.141004 | 12541.94 |
|  | 1787.968 | 798.8861 | 88.31031 | 345.8688 | 12534.31 | 333.8253 | 5.24E-02 | 0.142646 | 14531.86 |
|  | 1789.842 | 5003.827 | 551.7021 | 12153.93 | 9655.588 | 2795.835 | 0.32848 | 0.185369 | 25506.53 |
|  | 1813.845 | 52.65595 | 5.432377 | 281.0152 | 11064.38 | 23.27669 | 3.46E-03 | 0.163935 | 76.36967 |
|  | 1823.929 | 89.41501 | 9.1734 | 519.2517 | 16824.11 | 32.9617 | 5.87E-03 | 0.108412 | 122.2633 |
|  | 1830.96 | 55.38509 | 5.613319 | 300.4859 | 17172.57 | 19.08172 | 3.64E-03 | 0.106621 | 68.35146 |
|  | 1835.862 | 77.89822 | 8.053293 | 185.8925 | 12275.13 | 38.96359 | 5.11E-03 | 0.149559 | 283.0097 |
|  | 1852.902 | 6104.65 | 628.0832 | 1491.556 | 10227.91 | 3422.752 | 0.400744 | 0.181161 | 545190.8 |
|  | 1867.917 | 494.2071 | 49.98191 | 6720.886 | 14555.16 | 211.0183 | 3.24E-02 | 0.128334 | 975.5999 |
|  | 1909.924 | 155.6962 | 19.47238 | 527.8693 | 11462.4 | 81.76965 | 1.02E-02 | 0.166625 | 512.4552 |
|  | 1957.043 | 46.9237 | 10.40299 | 58.11145 | 14625.98 | 23.45638 | 3.08E-03 | 0.133806 | 382.7448 |
|  | 1959.06 | 159.9655 | 36.37556 | 573.9876 | 14136.6 | 69.89954 | 1.05E-02 | 0.138581 | 411.2603 |
|  | 1969.935 | 97.83601 | 26.67952 | 194.1785 | 13749.22 | 38.03608 | 6.42E-03 | 0.143276 | 450.713 |
|  | 1970.943 | 95.34843 | 26.23166 | 641.5607 | 17288.23 | 42.30553 | 6.26E-03 | 0.114005 | 401.7035 |
|  | 2290.039 | 31.86793 | 24.35069 | 481.2825 | 15220.17 | 14.2439 | 2.09E-03 | 0.150461 | 24.17837 |
|  |  |  |  |  |  |  |  |  |  |
| ID | m/z | Intens. | SN | Quality Fac. | Res. | Area | Rel. Intens. | FWHM | Chi^2 |
| 1236 | 720.6142 | 94.10022 | 13.60166 | 162.3663 | 10648.72 | 9.57608 | 4.26E-02 | 6.77E-02 | 138.6632 |
|  | 765.569 | 901.9755 | 130.0444 | 157.141 | 9171.769 | 128.2553 | 0.408666 | 8.35E-02 | 16811.24 |
|  | 767.5781 | 703.4022 | 98.63347 | 415.2563 | 9252.382 | 98.96416 | 0.318696 | 8.30E-02 | 8287.758 |
|  | 769.5776 | 134.1628 | 18.49895 | 111.5173 | 10439.84 | 15.48561 | 6.08E-02 | 7.37E-02 | 443.3806 |
|  | 774.4261 | 27.73823 | 3.94612 | 51.38401 | 6586.074 | 5.744147 | 1.26E-02 | 0.117585 | 24.09079 |
|  | 804.2829 | 62.78761 | 8.906594 | 274.2016 | 8704.053 | 10.55025 | 2.84E-02 | 9.24E-02 | 44.13165 |
|  | 810.3296 | 349.1063 | 48.44957 | 72.85341 | 9611.202 | 47.41527 | 0.158173 | 8.43E-02 | 6992.782 |
|  | 812.3226 | 476.8927 | 68.24878 | 150.6803 | 9889.685 | 67.56291 | 0.21607 | 8.21E-02 | 5567.365 |
|  | 814.3242 | 215.2209 | 30.86221 | 300.2574 | 9200.273 | 34.65727 | 9.75E-02 | 0.088511 | 1123.111 |
|  | 838.4803 | 53.59586 | 7.896313 | 205.2667 | 11579.91 | 7.152706 | 2.43E-02 | 7.24E-02 | 35.40412 |
|  | 856.5141 | 125.6715 | 19.48205 | 2427.666 | 8348.183 | 22.28192 | 5.69E-02 | 0.102599 | 46.95728 |
|  | 870.534 | 794.4617 | 127.311 | 6506.731 | 8192.044 | 144.233 | 0.359953 | 0.106266 | 2026.003 |
|  | 938.5401 | 25.96869 | 4.6102 | 70.05472 | 5384.116 | 8.455806 | 0.011766 | 0.174316 | 26.82518 |
|  | 950.5455 | 35.45448 | 6.344884 | 123.6886 | 6980.41 | 9.228939 | 1.61E-02 | 0.136173 | 37.06007 |
|  | 958.4998 | 60.83545 | 10.93235 | 332.0254 | 8420.144 | 12.81171 | 2.76E-02 | 0.113834 | 38.45802 |
|  | 978.5128 | 376.4474 | 68.58076 | 8694.25 | 9292.899 | 72.07431 | 0.17056 | 0.105297 | 263.3202 |
|  | 982.4229 | 46.18731 | 8.263572 | 269.5242 | 9191.701 | 11.1624 | 2.09E-02 | 0.106882 | 36.59334 |
|  | 994.5113 | 82.18096 | 15.12434 | 1094.548 | 9411.663 | 16.10766 | 3.72E-02 | 0.105668 | 22.21263 |
|  | 1037.529 | 32.38917 | 5.2058 | 151.0037 | 8977.374 | 9.149382 | 0.014675 | 0.115572 | 28.10265 |
|  | 1046.126 | 516.867 | 79.66256 | 173.3179 | 11423.2 | 92.44818 | 0.234181 | 9.16E-02 | 13478.99 |
|  | 1048.126 | 368.4725 | 56.70678 | 88.02735 | 11619.73 | 61.82473 | 0.166947 | 9.02E-02 | 5799.895 |
|  | 1050.127 | 117.8224 | 18.33622 | 68.87657 | 13795.94 | 17.21754 | 5.34E-02 | 7.61E-02 | 760.8365 |
|  | 1076.51 | 774.9631 | 125.9237 | 4250.252 | 11101.53 | 161.7984 | 0.351119 | 9.70E-02 | 4156.717 |
|  | 1092.523 | 77.59719 | 12.84494 | 1139.567 | 7945.403 | 21.01257 | 3.52E-02 | 0.137504 | 34.18799 |
|  | 1119.649 | 2207.124 | 383.5224 | 11414.92 | 9659.981 | 521.1161 | 1 | 0.115906 | 16762.97 |
|  | 1125.707 | 91.75217 | 16.29041 | 823.0846 | 9503.491 | 20.56915 | 4.16E-02 | 0.118452 | 37.22635 |
|  | 1156.636 | 43.24302 | 8.543 | 93.98201 | 10497.52 | 13.79605 | 1.96E-02 | 0.110182 | 96.02569 |
|  | 1158.598 | 31.29983 | 6.251584 | 62.81986 | 9350.911 | 7.848967 | 1.42E-02 | 0.123902 | 81.75558 |
|  | 1180.632 | 215.4035 | 45.82301 | 527.9047 | 10513.14 | 55.24069 | 9.76E-02 | 0.112301 | 1204.021 |
|  | 1219.688 | 18.9694 | 4.369244 | 68.67196 | 9147.978 | 5.227761 | 8.59E-03 | 0.133329 | 9.422834 |
|  | 1281.929 | 68.62914 | 15.46508 | 59.52515 | 16395.17 | 11.90388 | 3.11E-02 | 7.82E-02 | 307.908 |
|  | 1308.658 | 41.22942 | 9.233807 | 280.5614 | 10822.83 | 13.91267 | 1.87E-02 | 0.120916 | 39.67549 |
|  | 1327.72 | 52.0154 | 12.73941 | 342.6039 | 9773.697 | 17.14817 | 2.36E-02 | 0.135846 | 43.24576 |
|  | 1332.669 | 14.14078 | 3.619048 | 63.56374 | 6961.347 | 5.994878 | 6.41E-03 | 0.191438 | 12.54254 |
|  | 1487.758 | 54.13243 | 19.13984 | 1618.37 | 15840.16 | 14.8686 | 2.45E-02 | 9.39E-02 | 26.33895 |
|  | 1515.738 | 19.08403 | 6.774581 | 83.86105 | 19027.3 | 4.076647 | 8.65E-03 | 7.97E-02 | 24.29699 |
|  | 1552.811 | 49.19441 | 20.14594 | 1377.48 | 12798.99 | 15.40846 | 2.23E-02 | 0.121323 | 9.690038 |
|  | 1571.824 | 75.8331 | 32.08918 | 3275.402 | 12374.08 | 23.69929 | 3.44E-02 | 0.127026 | 37.68825 |
|  | 1575.858 | 25.17743 | 10.97122 | 155.1292 | 17730.95 | 7.019726 | 1.14E-02 | 8.89E-02 | 27.14812 |
|  | 1629.929 | 49.73258 | 24.17699 | 830.2605 | 12765.23 | 17.53163 | 2.25E-02 | 0.127685 | 19.46622 |
|  | 2237.201 | 88.12846 | 151.3114 | 9761.367 | 19829.59 | 34.99005 | 3.99E-02 | 0.112821 | 65.48631 |
|  |  |  |  |  |  |  |  |  |  |
| ID | m/z | Intens. | SN | Quality Fac. | Res. | Area | Rel. Intens. | FWHM | Chi^2 |
| 1348 | 765.4556 | 60.13845 | 11.47638 | 248.3848 | 8484.93 | 8.818857 | 1.85E-02 | 9.02E-02 | 40.28881 |
|  | 774.4071 | 31.65176 | 6.202688 | 162.2669 | 11053.91 | 3.973105 | 9.76E-03 | 7.01E-02 | 11.32276 |
|  | 804.2569 | 27.17883 | 5.051186 | 89.18514 | 8275.171 | 5.469335 | 8.38E-03 | 9.72E-02 | 16.27169 |
|  | 826.4739 | 125.1759 | 22.32282 | 701.5433 | 9415.238 | 17.44537 | 3.86E-02 | 8.78E-02 | 65.34545 |
|  | 833.4455 | 39.18425 | 7.09407 | 217.5343 | 11653.28 | 5.249115 | 1.21E-02 | 7.15E-02 | 20.56871 |
|  | 838.4584 | 58.67562 | 10.52731 | 283.9657 | 12358.93 | 7.518418 | 1.81E-02 | 6.78E-02 | 32.19023 |
|  | 856.5247 | 124.4803 | 23.26764 | 435.2647 | 11440.77 | 15.63319 | 0.03837 | 7.49E-02 | 92.27472 |
|  | 870.5418 | 1053.475 | 207.9172 | 4581.274 | 10410.55 | 149.568 | 0.324724 | 8.36E-02 | 3264.715 |
|  | 1217.676 | 14.70324 | 4.129528 | 76.12333 | 9559.293 | 4.8026 | 4.53E-03 | 0.127381 | 10.97888 |
|  | 1226.716 | 36.16836 | 10.11243 | 195.7513 | 10951.19 | 9.121776 | 1.11E-02 | 0.112017 | 27.37696 |
|  | 1277.731 | 70.20872 | 16.44486 | 504.5442 | 11645.82 | 17.29172 | 2.16E-02 | 0.109716 | 46.97596 |
|  | 1290.707 | 389.9758 | 90.4803 | 6865.977 | 13321.59 | 84.57102 | 0.120207 | 9.69E-02 | 461.8767 |
|  | 1295.749 | 39.39529 | 8.926286 | 344.865 | 9545.46 | 12.10872 | 1.21E-02 | 0.135745 | 22.86511 |
|  | 1327.731 | 44.43269 | 10.13329 | 234.6721 | 11964.67 | 11.46304 | 1.37E-02 | 0.110971 | 41.80585 |
|  | 1330.68 | 138.1119 | 32.50285 | 2490.959 | 13839.25 | 31.74742 | 0.042572 | 9.62E-02 | 71.24162 |
|  | 1349.759 | 70.36547 | 17.34307 | 1396.694 | 11177.07 | 19.65683 | 2.17E-02 | 0.120761 | 18.55171 |
|  | 1377.744 | 24.87412 | 6.586595 | 96.75832 | 33205.96 | 3.953227 | 7.67E-03 | 0.041491 | 24.53435 |
|  | 1443.781 | 602.5673 | 146.2208 | 11938.66 | 13524.78 | 151.0093 | 0.185736 | 0.106751 | 1148.808 |
|  | 1449.733 | 117.9532 | 27.85705 | 1932.077 | 13019.53 | 32.45079 | 3.64E-02 | 0.111351 | 109.2272 |
|  | 1482.923 | 119.0885 | 29.74762 | 553.2393 | 13380.46 | 32.18228 | 3.67E-02 | 0.110828 | 143.1721 |
|  | 1618.882 | 1049.832 | 330.2221 | 14894.92 | 14969.66 | 298.2241 | 0.323602 | 0.108144 | 3986.033 |
|  | 1671.92 | 24.05434 | 8.379718 | 487.6832 | 14704.67 | 8.469304 | 7.41E-03 | 0.1137 | 7.365752 |
|  | 1680.865 | 53.4342 | 19.1133 | 1296.258 | 16187 | 15.06759 | 1.65E-02 | 0.10384 | 29.77963 |
|  | 1728.916 | 62.70688 | 25.59344 | 3297.736 | 17221.85 | 19.61765 | 1.93E-02 | 0.100391 | 21.08587 |
|  | 1783.96 | 39.26853 | 19.9845 | 2176.467 | 18527.61 | 11.73765 | 1.21E-02 | 9.63E-02 | 13.1015 |
|  | 1869.962 | 10.77383 | 6.304817 | 179.1352 | 13906.09 | 4.869515 | 3.32E-03 | 0.134471 | 5.381684 |
|  | 1914.005 | 14.58904 | 9.718936 | 161.6984 | 14898.15 | 5.549849 | 4.50E-03 | 0.128473 | 11.07963 |
|  | 1934.975 | 13.36834 | 9.455115 | 147.3436 | 18077.94 | 4.461276 | 4.12E-03 | 0.107035 | 8.717314 |
|  | 2233.086 | 22.13664 | 21.92549 | 959.3119 | 16895.88 | 8.434816 | 6.82E-03 | 0.132168 | 9.510392 |
|  | 2293.076 | 10.18627 | 10.06903 | 146.6629 | 21982.08 | 2.938828 | 3.14E-03 | 0.104316 | 4.396894 |
|  | 2300.182 | 11.27572 | 12.688 | 113.9599 | 24314.24 | 4.039257 | 3.48E-03 | 9.46E-02 | 7.707395 |
|  | 2338.14 | 184.6305 | 232.8635 | 3741.932 | 22512.25 | 66.24526 | 5.69E-02 | 0.103861 | 654.24 |
|  | 2707.418 | 4.622009 | 21.97262 | 95.30152 | 21072.59 | 2.02998 | 1.42E-03 | 0.128481 | 3.199857 |
|  |  |  |  |  |  |  |  |  |  |
| ID | m/z | Intens. | SN | Quality Fac. | Res. | Area | Rel. Intens. | FWHM | Chi^2 |
| 1463 | 759.4416 | 60.96619 | 9.429894 | 153.2592 | 6715.116 | 11.486 | 4.61E-03 | 0.113094 | 75.35855 |
|  | 798.4474 | 33.31192 | 4.83283 | 84.27943 | 5076.874 | 9.180277 | 2.52E-03 | 0.157271 | 28.38949 |
|  | 804.2801 | 134.6308 | 19.39827 | 764.4725 | 7045.555 | 27.30158 | 1.02E-02 | 0.114154 | 81.99957 |
|  | 813.4418 | 56.48459 | 8.003446 | 155.2145 | 7077.864 | 11.32924 | 4.27E-03 | 0.114928 | 56.88518 |
|  | 827.4907 | 84.45289 | 11.69073 | 718.9054 | 7922.963 | 13.06737 | 6.38E-03 | 0.104442 | 27.3894 |
|  | 838.4693 | 47.50658 | 6.740388 | 194.1362 | 5987.592 | 10.59531 | 3.59E-03 | 0.140034 | 38.61482 |
|  | 845.5225 | 2480.231 | 355.4916 | 4683.738 | 7126.926 | 499.3043 | 0.187499 | 0.118638 | 16724.18 |
|  | 856.5134 | 67.13013 | 10.04083 | 314.2587 | 9298.228 | 10.5754 | 5.07E-03 | 9.21E-02 | 42.68843 |
|  | 870.5414 | 232.3906 | 35.72313 | 1249.134 | 8750.544 | 39.45533 | 1.76E-02 | 9.95E-02 | 147.1134 |
|  | 877.4795 | 44.94405 | 7.100509 | 105.1571 | 9738.623 | 7.696616 | 3.40E-03 | 9.01E-02 | 45.31825 |
|  | 930.5251 | 97.71429 | 15.07828 | 562.7703 | 7941.86 | 18.30123 | 0.007387 | 0.117167 | 55.79243 |
|  | 933.5281 | 61.04025 | 9.447263 | 378.2045 | 8224.293 | 13.74689 | 4.61E-03 | 0.113509 | 35.48858 |
|  | 950.5231 | 50.68809 | 7.385248 | 179.4834 | 12008.48 | 8.243919 | 3.83E-03 | 7.92E-02 | 48.5911 |
|  | 955.6039 | 48.6478 | 6.960829 | 187.6076 | 7752.608 | 10.97738 | 3.68E-03 | 0.123262 | 37.7039 |
|  | 959.5423 | 100.0389 | 13.91077 | 555.6737 | 9082.771 | 19.19244 | 7.56E-03 | 0.105644 | 57.74263 |
|  | 974.5336 | 228.9858 | 29.92643 | 1508.369 | 8862.229 | 45.06438 | 1.73E-02 | 0.109965 | 129.1526 |
|  | 991.5571 | 7509.357 | 943.5601 | 24247.03 | 7343.434 | 1901.326 | 0.567688 | 0.135026 | 77786.93 |
|  | 1007.549 | 407.4166 | 53.2804 | 1659.118 | 9742.41 | 78.04152 | 3.08E-02 | 0.103419 | 354.21 |
|  | 1023.535 | 82.14371 | 11.28365 | 378.3367 | 9397.443 | 17.86806 | 6.21E-03 | 0.108916 | 67.32576 |
|  | 1046.557 | 209.9432 | 31.79507 | 531.0539 | 9325.576 | 47.67136 | 1.59E-02 | 0.112224 | 354.0015 |
|  | 1048.588 | 114.9179 | 17.81218 | 482.9016 | 10847.69 | 19.91992 | 8.69E-03 | 9.67E-02 | 101.7899 |
|  | 1089.637 | 45.28681 | 6.850575 | 142.3841 | 11358.45 | 9.090243 | 3.42E-03 | 9.59E-02 | 45.94879 |
|  | 1095.535 | 541.3031 | 81.48938 | 396.0339 | 8471.199 | 137.9527 | 4.09E-02 | 0.129325 | 10853.75 |
|  | 1096.581 | 1237.829 | 186.1646 | 1826.648 | 8443.755 | 325.5652 | 9.36E-02 | 0.129869 | 10859.25 |
|  | 1112.598 | 81.41112 | 11.89746 | 442.8316 | 10356.21 | 19.66427 | 6.15E-03 | 0.107433 | 58.3745 |
|  | 1181.556 | 13227.97 | 1495.958 | 56634.26 | 7428.88 | 4323.573 | 1 | 0.159049 | 146309.3 |
|  | 1187.648 | 29.31678 | 3.467477 | 65.97882 | 4987.348 | 14.91457 | 2.22E-03 | 0.238132 | 36.51734 |
|  | 1226.653 | 67.52004 | 10.17432 | 664.54 | 9487.016 | 19.45551 | 5.10E-03 | 0.129298 | 29.9193 |
|  | 1280.716 | 102.6423 | 10.11955 | 982.8222 | 10392.57 | 29.19805 | 7.76E-03 | 0.123234 | 52.45217 |
|  | 1286.764 | 95.79946 | 8.870587 | 461.3281 | 7460.993 | 33.70465 | 7.24E-03 | 0.172466 | 110.8563 |
|  | 1296.696 | 92.06597 | 7.935553 | 634.5926 | 8625.784 | 29.42512 | 6.96E-03 | 0.150328 | 65.54319 |
|  | 1308.711 | 436.9298 | 36.56324 | 9842.037 | 9102.62 | 151.6995 | 3.30E-02 | 0.143773 | 305.0569 |
|  | 1323.787 | 13197.05 | 1187.018 | 89739.72 | 7796.488 | 5133.609 | 0.997663 | 0.169793 | 58315.91 |
|  | 1330.737 | 54.52491 | 5.119175 | 229.8573 | 8504.253 | 18.59042 | 4.12E-03 | 0.156479 | 66.94613 |
|  | 1340.81 | 4557.469 | 456.073 | 31025.67 | 9000.495 | 1577.66 | 0.344533 | 0.148971 | 35895.94 |
|  | 1347.733 | 47.05285 | 4.813042 | 174.233 | 11385.92 | 12.92363 | 3.56E-03 | 0.118368 | 49.98671 |
|  | 1385.721 | 111.1564 | 13.96581 | 531.4525 | 10443.65 | 34.28798 | 8.40E-03 | 0.132686 | 118.5707 |
|  | 1388.69 | 32.13686 | 3.970825 | 93.85893 | 13840.93 | 7.795332 | 2.43E-03 | 0.100332 | 39.13917 |
|  | 1413.743 | 2570.828 | 339.931 | 48355.97 | 10188.98 | 871.4113 | 0.194348 | 0.138752 | 8145.392 |
|  | 1416.782 | 92.84379 | 11.92886 | 315.6079 | 15586.36 | 19.41046 | 7.02E-03 | 9.09E-02 | 124.3994 |
|  | 1417.764 | 44.29694 | 5.631321 | 92.30168 | 17489.13 | 6.280288 | 3.35E-03 | 8.11E-02 | 72.16725 |
|  | 1423.694 | 42.19827 | 5.602907 | 231.6662 | 12285.69 | 12.58295 | 3.19E-03 | 0.115882 | 24.07916 |
|  | 1435.731 | 36.39389 | 4.958832 | 111.6878 | 16209.24 | 9.011266 | 2.75E-03 | 8.86E-02 | 55.15218 |
|  | 1439.784 | 28.58151 | 3.946738 | 83.88369 | 16231.94 | 6.860044 | 2.16E-03 | 8.87E-02 | 47.30605 |
|  | 1466.795 | 32.21347 | 4.655568 | 58.20463 | 11439.09 | 10.57947 | 2.44E-03 | 0.128227 | 53.27644 |
|  | 1469.803 | 38.183 | 5.657357 | 170.4702 | 10521.03 | 12.6595 | 2.89E-03 | 0.139701 | 35.38639 |
|  | 1479.764 | 30.04471 | 4.541808 | 78.86508 | 11321.29 | 9.711135 | 2.27E-03 | 0.130706 | 34.05021 |
|  | 1485.797 | 46.07062 | 7.092201 | 321.4789 | 13391.19 | 13.0519 | 3.48E-03 | 0.110953 | 34.30902 |
|  | 1502.812 | 39.74801 | 6.271778 | 100.4719 | 11719.83 | 12.43774 | 3.00E-03 | 0.128228 | 58.03092 |
|  | 1525.761 | 104.4202 | 18.01832 | 996.973 | 12022.6 | 34.48949 | 7.89E-03 | 0.126908 | 60.1895 |
|  | 1534.832 | 57.36791 | 10.12527 | 516.5034 | 14359.43 | 18.57321 | 4.34E-03 | 0.106887 | 37.57663 |
|  | 1549.771 | 289.5644 | 54.39125 | 6011.575 | 13063.82 | 99.58929 | 2.19E-02 | 0.118631 | 391.9433 |
|  | 1576.829 | 19.26673 | 3.994014 | 54.40046 | 12781.18 | 6.626999 | 1.46E-03 | 0.123371 | 18.66139 |
|  | 1581.922 | 42.78779 | 8.764934 | 279.3109 | 11775.51 | 13.891 | 3.23E-03 | 0.13434 | 34.07306 |
|  | 1609.904 | 24.77648 | 5.516793 | 214.4113 | 7982.614 | 14.92031 | 1.87E-03 | 0.201676 | 24.22486 |
|  | 1639.895 | 22.53703 | 5.2875 | 187.2521 | 10824.97 | 9.528476 | 1.70E-03 | 0.151492 | 12.15539 |
|  | 1670.879 | 26.81906 | 6.740584 | 217.7563 | 10018.96 | 12.51099 | 2.03E-03 | 0.166772 | 27.52354 |
|  | 1700.918 | 23.91579 | 6.325575 | 169.6035 | 11001.4 | 10.78576 | 1.81E-03 | 0.154609 | 18.59398 |
|  | 1703.836 | 17.5014 | 4.626204 | 256.5317 | 9209.93 | 8.7869 | 1.32E-03 | 0.185 | 10.01192 |
|  | 1889.088 | 28.73314 | 9.062621 | 400.4592 | 10236.59 | 16.00736 | 2.17E-03 | 0.184543 | 21.43871 |
|  | 1918.016 | 255.6812 | 78.69005 | 7273.193 | 14645.41 | 116.7691 | 1.93E-02 | 0.130964 | 531.1513 |
|  | 1975.114 | 38.32008 | 11.46544 | 632.5342 | 12237.89 | 18.86028 | 2.90E-03 | 0.161393 | 22.49151 |
|  | 2001.021 | 1598.22 | 500.6176 | 14903.38 | 12469.22 | 850.1911 | 0.120821 | 0.160477 | 10303.69 |
|  | 2022.956 | 498.5479 | 167.0553 | 7678.165 | 13521.04 | 254.1582 | 3.77E-02 | 0.149615 | 2255.626 |
|  | 2051.957 | 17.64176 | 6.204635 | 446.622 | 12265.67 | 10.72307 | 1.33E-03 | 0.167293 | 7.97355 |
|  | 2099.99 | 12.53303 | 5.123701 | 75.16722 | 15194.58 | 6.041104 | 9.47E-04 | 0.138207 | 9.877421 |
|  | 2115.983 | 17.77974 | 7.719245 | 528.7994 | 11251.34 | 11.60414 | 1.34E-03 | 0.188065 | 5.402082 |
|  | 2346.231 | 175.7364 | 100.3113 | 6144.8 | 15645.42 | 94.8468 | 1.33E-02 | 0.149963 | 303.4654 |
|  | 2366.115 | 556.4082 | 314.6416 | 12744.17 | 14690.51 | 302.7606 | 4.21E-02 | 0.161064 | 1864.242 |
|  | 2463.379 | 96.40902 | 67.93137 | 1818.109 | 16218.55 | 49.79353 | 7.29E-03 | 0.151886 | 202.2893 |
|  | 3057.454 | 15.27467 | 78.84043 | 513.5457 | 16581.87 | 10.8106 | 1.15E-03 | 0.184385 | 13.53509 |
|  |  |  |  |  |  |  |  |  |  |
| ID | m/z | Intens. | SN | Quality Fac. | Res. | Area | Rel. Intens. | FWHM | Chi^2 |
| 1487 | 765.5875 | 90.30761 | 12.96694 | 89.71093 | 12702.8 | 9.066942 | 1.03E-02 | 6.03E-02 | 215.5735 |
|  | 767.5917 | 68.02379 | 9.618394 | 108.1878 | 11891.2 | 7.305259 | 7.77E-03 | 6.46E-02 | 115.6521 |
|  | 774.3971 | 35.27278 | 4.877264 | 59.84838 | 8459.185 | 5.107747 | 4.03E-03 | 0.091545 | 37.05265 |
|  | 804.2886 | 73.75138 | 10.45843 | 489.9896 | 6677.396 | 15.11689 | 8.42E-03 | 0.120449 | 35.58664 |
|  | 838.4858 | 45.78543 | 6.661718 | 87.63266 | 10786.69 | 7.60805 | 5.23E-03 | 7.77E-02 | 70.12682 |
|  | 856.5411 | 141.6515 | 20.96779 | 416.5398 | 10745.7 | 18.68555 | 1.62E-02 | 7.97E-02 | 132.9816 |
|  | 870.5581 | 460.25 | 71.79005 | 1016.555 | 8656.396 | 77.38444 | 0.052571 | 0.100568 | 945.049 |
|  | 903.4656 | 586.4184 | 97.09399 | 1415.383 | 9429.788 | 105.6861 | 6.70E-02 | 9.58E-02 | 1401.491 |
|  | 906.4914 | 30.39473 | 5.039543 | 170.6221 | 8419.014 | 5.793292 | 3.47E-03 | 0.107672 | 15.04873 |
|  | 950.5492 | 45.34649 | 7.740683 | 289.8342 | 10851.53 | 7.559512 | 5.18E-03 | 8.76E-02 | 17.1952 |
|  | 1025.546 | 222.0105 | 39.10721 | 1454.066 | 9782.544 | 46.5814 | 2.54E-02 | 0.104834 | 229.9346 |
|  | 1096.552 | 40.01395 | 5.821088 | 138.5292 | 7549.423 | 11.08536 | 4.57E-03 | 0.14525 | 30.53844 |
|  | 1099.576 | 27.54167 | 3.975318 | 87.19197 | 6115.012 | 10.25936 | 3.15E-03 | 0.179816 | 25.28326 |
|  | 1114.58 | 8754.754 | 1205.851 | 45252.05 | 8436.858 | 2309.971 | 1 | 0.132108 | 71057.8 |
|  | 1130.564 | 32.34035 | 4.519875 | 56.89433 | 8034.585 | 9.879568 | 3.69E-03 | 0.140712 | 54.25962 |
|  | 1142.565 | 57.75775 | 8.161102 | 553.7295 | 8890.735 | 14.90258 | 6.60E-03 | 0.128512 | 24.58276 |
|  | 1151.633 | 3550.458 | 510.2863 | 17535.29 | 9312.566 | 895.3886 | 0.405546 | 0.123664 | 23732.59 |
|  | 1186.549 | 234.8312 | 35.9649 | 2057.282 | 10366.07 | 59.47487 | 2.68E-02 | 0.114465 | 296.9949 |
|  | 1243.664 | 77.54246 | 15.08026 | 374.0751 | 16247.49 | 17.38677 | 8.86E-03 | 7.65E-02 | 80.2464 |
|  | 1264.645 | 38.64425 | 7.147819 | 108.8027 | 8974.305 | 12.83194 | 4.41E-03 | 0.140918 | 22.48594 |
|  | 1272.466 | 48.84898 | 9.044374 | 684.1904 | 6161.706 | 24.41449 | 5.58E-03 | 0.206512 | 21.24274 |
|  | 1312.654 | 1102.877 | 187.1972 | 46432.97 | 10655.14 | 296.2823 | 0.125975 | 0.123194 | 940.7861 |
|  | 1328.667 | 339.6737 | 56.54817 | 2899.737 | 9894.498 | 103.2098 | 3.88E-02 | 0.134283 | 718.3523 |
|  | 1342.687 | 30.98127 | 5.012094 | 90.92142 | 11684.96 | 8.672015 | 3.54E-03 | 0.114907 | 40.28086 |
|  | 1360.693 | 4777.998 | 769.7535 | 27219.1 | 10510.78 | 1479.349 | 0.54576 | 0.129457 | 39191.98 |
|  | 1369.674 | 44.04197 | 6.988076 | 218.8118 | 10339.71 | 14.68454 | 5.03E-03 | 0.132467 | 51.83742 |
|  | 1416.674 | 49.19248 | 9.193355 | 118.465 | 11154.11 | 15.37039 | 5.62E-03 | 0.127009 | 82.20708 |
|  | 1432.661 | 44.47707 | 8.823028 | 163.2516 | 12925.13 | 12.53139 | 5.08E-03 | 0.110843 | 47.74751 |
|  | 1438.705 | 25.66302 | 5.238574 | 71.97954 | 11719.54 | 10.10134 | 2.93E-03 | 0.122761 | 32.70814 |
|  | 1440.631 | 21.30113 | 4.207875 | 51.25881 | 26067.77 | 2.888984 | 2.43E-03 | 5.53E-02 | 22.33649 |
|  | 1455.647 | 39.594 | 7.383095 | 593.7637 | 14374.04 | 10.89343 | 4.52E-03 | 0.101269 | 12.06836 |
|  | 1485.728 | 99.18217 | 17.14549 | 755.7372 | 11632.92 | 35.02038 | 1.13E-02 | 0.127717 | 79.40408 |
|  | 1487.66 | 68.93201 | 11.76998 | 64.13184 | 7708.187 | 24.56364 | 7.87E-03 | 0.192997 | 568.3703 |
|  | 1490.732 | 1324.889 | 226.2077 | 25009.66 | 12314.14 | 412.5253 | 0.151334 | 0.121059 | 4082.548 |
|  | 1494.731 | 36.5399 | 6.146015 | 259.9219 | 10644.14 | 13.24563 | 4.17E-03 | 0.140428 | 32.25548 |
|  | 1496.781 | 48.698 | 7.911594 | 655.4 | 11661.11 | 14.57537 | 5.56E-03 | 0.128357 | 19.22392 |
|  | 1502.747 | 513.6596 | 83.92403 | 8707.036 | 13119.07 | 149.9505 | 5.87E-02 | 0.114547 | 973.2212 |
|  | 1512.763 | 33.29034 | 5.4374 | 207.3459 | 11815.02 | 11.66037 | 3.80E-03 | 0.128037 | 23.78334 |
|  | 1558.811 | 36.07928 | 8.018521 | 375.3193 | 15913.05 | 9.81476 | 4.12E-03 | 9.80E-02 | 19.43472 |
|  | 1574.839 | 119.4742 | 29.36456 | 1858.119 | 15286.98 | 31.72279 | 1.36E-02 | 0.103018 | 42.28249 |
|  | 1646.827 | 241.4699 | 54.27449 | 5184.366 | 14507.63 | 84.95045 | 2.76E-02 | 0.113515 | 348.9151 |
|  | 1652.891 | 220.1084 | 47.28363 | 6937.389 | 11890.97 | 80.6244 | 2.51E-02 | 0.139004 | 159.7032 |
|  | 1668.883 | 70.62947 | 16.18677 | 637.3652 | 13841.74 | 23.87054 | 8.07E-03 | 0.120569 | 50.80573 |
|  | 1678.826 | 158.3858 | 37.40952 | 1876.687 | 13332.48 | 55.0682 | 1.81E-02 | 0.12592 | 91.75815 |
|  | 1734.809 | 31.70226 | 10.37604 | 174.0368 | 12662.58 | 12.53636 | 3.62E-03 | 0.137003 | 39.82321 |
|  | 1735.823 | 35.02867 | 11.28766 | 225.5691 | 15103.02 | 13.26442 | 4.00E-03 | 0.114932 | 42.11165 |
|  | 1750.819 | 32.21782 | 10.43887 | 122.6511 | 11727.32 | 14.35666 | 3.68E-03 | 0.149294 | 19.44704 |
|  | 1818.921 | 21.88531 | 7.646321 | 326.6362 | 11526.23 | 12.87849 | 2.50E-03 | 0.157807 | 14.16499 |
|  | 1834.925 | 446.0623 | 167.6544 | 7190.531 | 14070.68 | 182.1422 | 5.10E-02 | 0.130408 | 1439.021 |
|  | 2099.066 | 193.7587 | 221.7951 | 4853.117 | 16524.53 | 76.69025 | 2.21E-02 | 0.127027 | 526.2949 |
|  | 3346.619 | 5.422867 | 283.6081 | 224.5072 | 22398.33 | 3.138107 | 6.19E-04 | 0.149414 | 4.475865 |
|  |  |  |  |  |  |  |  |  |  |
| ID | m/z | Intens. | SN | Quality Fac. | Res. | Area | Rel. Intens. | FWHM | Chi^2 |
| 2235 | 736.401 | 150.0303 | 13.68551 | 306.1006 | 6238.083 | 31.56335 | 1.02E-02 | 0.118049 | 249.8228 |
|  | 780.3835 | 75.76238 | 6.520145 | 263.6275 | 6975.366 | 14.47878 | 5.14E-03 | 0.111877 | 51.48017 |
|  | 796.3869 | 57.8978 | 4.716544 | 116.333 | 5226.445 | 20.17614 | 3.93E-03 | 0.152376 | 85.84911 |
|  | 846.4461 | 63.79611 | 4.530316 | 166.5821 | 4948.182 | 19.63853 | 4.33E-03 | 0.171062 | 63.19402 |
|  | 856.5328 | 2238.874 | 154.4585 | 4251.748 | 7114.553 | 474.5007 | 0.151923 | 0.120392 | 11517.77 |
|  | 870.5411 | 4897.307 | 331.2639 | 25604.95 | 6822.863 | 1126.292 | 3.32E-01 | 0.127592 | 24957.99 |
|  | 897.5546 | 1135.274 | 77.39452 | 4795.422 | 7194.831 | 247.5449 | 7.70E-02 | 0.12475 | 3532.565 |
|  | 903.4858 | 128.1223 | 9.028896 | 97.78225 | 7939.205 | 26.04427 | 8.69E-03 | 0.113801 | 595.133 |
|  | 919.5385 | 74.82738 | 5.525636 | 113.0797 | 8547.347 | 14.81259 | 5.08E-03 | 0.107582 | 120.6672 |
|  | 928.4837 | 361.5725 | 26.83726 | 1748.263 | 7100.672 | 82.02005 | 2.45E-02 | 0.13076 | 278.8092 |
|  | 932.5011 | 172.8172 | 13.21792 | 385.3406 | 7918.566 | 36.89291 | 1.17E-02 | 0.117761 | 285.1373 |
|  | 973.5207 | 90.09618 | 6.734176 | 125.8098 | 9889.587 | 16.45657 | 6.11E-03 | 9.84E-02 | 159.253 |
|  | 976.5147 | 94.48133 | 7.041407 | 469.844 | 9637.797 | 17.22539 | 6.41E-03 | 0.101321 | 61.33965 |
|  | 982.4381 | 175.7791 | 12.68421 | 676.9106 | 9627.484 | 41.07689 | 1.19E-02 | 0.102045 | 168.8726 |
|  | 1015.588 | 252.4899 | 16.99812 | 901.9514 | 8018.141 | 61.17564 | 1.71E-02 | 0.126661 | 287.4473 |
|  | 1034.571 | 102.5873 | 6.526291 | 237.9118 | 5844.12 | 24.10059 | 6.96E-03 | 0.177028 | 171.3677 |
|  | 1042.589 | 98.3292 | 6.089173 | 93.5589 | 7536.815 | 23.90486 | 6.67E-03 | 0.138333 | 292.384 |
|  | 1051.557 | 886.0402 | 53.18684 | 11182.1 | 7608.974 | 241.7182 | 6.01E-02 | 0.1382 | 1226.945 |
|  | 1055.589 | 80.23068 | 4.79339 | 119.0879 | 6793.611 | 24.60332 | 5.44E-03 | 0.15538 | 168.545 |
|  | 1060.563 | 97.71151 | 5.801415 | 113.3502 | 6647.68 | 27.39057 | 6.63E-03 | 0.159539 | 253.3615 |
|  | 1063.507 | 121.9705 | 7.274735 | 130.5705 | 8356.059 | 36.7078 | 8.28E-03 | 0.127274 | 517.6235 |
|  | 1078.597 | 75.42452 | 4.525836 | 143.6188 | 7831.199 | 19.2235 | 5.12E-03 | 0.137731 | 111.8114 |
|  | 1092.509 | 120.8901 | 7.186465 | 256.4448 | 6069.912 | 33.23959 | 8.20E-03 | 0.179988 | 245.3707 |
|  | 1095.644 | 159.4985 | 9.518988 | 368.5961 | 7615.174 | 44.9949 | 1.08E-02 | 0.143876 | 295.144 |
|  | 1108.555 | 302.0581 | 18.0903 | 620.8993 | 9069.642 | 74.45849 | 2.05E-02 | 0.122227 | 608.8618 |
|  | 1113.547 | 244.378 | 14.60154 | 1056.69 | 8125.925 | 69.75126 | 1.66E-02 | 0.137036 | 250.1951 |
|  | 1122.574 | 146.8896 | 8.626786 | 245.2305 | 9217.523 | 37.63874 | 9.97E-03 | 0.121787 | 351.2372 |
|  | 1156.61 | 1313.27 | 72.03184 | 13256.41 | 8014.195 | 382.2755 | 8.91E-02 | 0.14432 | 3690.352 |
|  | 1161.612 | 210.5206 | 11.55126 | 180.8283 | 8346.519 | 56.9818 | 1.43E-02 | 0.139173 | 1111.834 |
|  | 1163.605 | 168.8839 | 9.309051 | 154.9419 | 8900.865 | 45.44534 | 1.15E-02 | 0.130729 | 746.8456 |
|  | 1201.64 | 114.428 | 6.388087 | 110.1969 | 9848.502 | 29.52635 | 7.76E-03 | 0.122012 | 308.8299 |
|  | 1225.693 | 271.0776 | 15.51616 | 1307.758 | 9228.241 | 74.43728 | 1.84E-02 | 0.13282 | 258.9596 |
|  | 1245.611 | 255.8509 | 13.90081 | 742.8649 | 10004.35 | 73.71212 | 1.74E-02 | 0.124507 | 396.7699 |
|  | 1248.663 | 185.6066 | 9.899629 | 284.6147 | 8225.357 | 58.77102 | 1.26E-02 | 1.52E-01 | 552.1314 |
|  | 1262.657 | 130.486 | 6.420174 | 88.14913 | 11582.69 | 37.79164 | 8.85E-03 | 0.109012 | 411.5532 |
|  | 1285.698 | 2070.542 | 88.92041 | 2642.744 | 7405.378 | 815.6705 | 1.41E-01 | 1.74E-01 | 9051.581 |
|  | 1297.668 | 128.1056 | 5.189888 | 106.039 | 8903.359 | 41.74158 | 8.69E-03 | 0.14575 | 662.4262 |
|  | 1311.715 | 291.7283 | 11.64864 | 211.9707 | 8952.125 | 94.38244 | 1.98E-02 | 0.146526 | 2031.304 |
|  | 1313.711 | 976.2616 | 38.40359 | 7553.559 | 9306.447 | 326.0545 | 6.62E-02 | 0.141161 | 2420.213 |
|  | 1316.696 | 1661.53 | 66.90548 | 52291 | 8747.458 | 574.5952 | 1.13E-01 | 0.150523 | 1447.463 |
|  | 1329.68 | 133.6949 | 5.426379 | 244.5911 | 11667.74 | 40.00445 | 9.07E-03 | 1.14E-01 | 275.1124 |
|  | 1373.666 | 158.4138 | 7.06571 | 125.5362 | 7210.585 | 51.20064 | 1.07E-02 | 0.190507 | 909.5622 |
|  | 1375.749 | 1284.86 | 58.13786 | 33982.21 | 9493.782 | 442.9598 | 8.72E-02 | 0.144911 | 1323.608 |
|  | 1403.685 | 148.6354 | 7.224802 | 289.8384 | 11449.43 | 42.95741 | 1.01E-02 | 0.122599 | 396.3276 |
|  | 1413.72 | 348.417 | 18.00819 | 1498.176 | 10175.23 | 107.4367 | 2.36E-02 | 0.138937 | 405.5898 |
|  | 1425.754 | 185.2704 | 9.975404 | 234.9979 | 7997.087 | 70.40655 | 1.26E-02 | 0.178284 | 874.9913 |
|  | 1427.779 | 127.162 | 6.858361 | 226.0273 | 10405.21 | 38.15547 | 8.63E-03 | 1.37E-01 | 355.5651 |
|  | 1430.75 | 279.0022 | 15.31488 | 927.0823 | 10861.02 | 80.70718 | 1.89E-02 | 0.131733 | 419.699 |
|  | 1462.754 | 98.25224 | 5.747832 | 164.9276 | 9906.273 | 32.27625 | 6.67E-03 | 0.147659 | 240.6452 |
|  | 1466.753 | 70.96769 | 4.180807 | 101.6055 | 10397.49 | 23.62405 | 4.82E-03 | 0.141068 | 201.3944 |
|  | 1523.778 | 270.9874 | 17.90453 | 643.976 | 11563.4 | 102.1019 | 1.84E-02 | 0.131776 | 653.7539 |
|  | 1539.792 | 155.7259 | 10.41342 | 166.0089 | 12902.37 | 54.65857 | 1.06E-02 | 0.119342 | 773.0832 |
|  | 1551.76 | 1605.832 | 108.0327 | 42445.62 | 10526.92 | 611.6652 | 1.09E-01 | 0.147409 | 2365.457 |
|  | 1556.848 | 232.4032 | 15.68269 | 1012.556 | 11715.34 | 86.43359 | 1.58E-02 | 0.13289 | 335.135 |
|  | 1576.864 | 707.8994 | 48.0874 | 10476.26 | 10987.98 | 265.0857 | 4.80E-02 | 0.143508 | 1414.813 |
|  | 1643.786 | 105.3594 | 7.198687 | 636.9932 | 11253.41 | 39.24165 | 7.15E-03 | 0.14607 | 90.99461 |
|  | 1660.837 | 92.91374 | 6.346422 | 173.3317 | 10954.54 | 30.63956 | 6.30E-03 | 0.151612 | 309.4619 |
|  | 1671.878 | 114.3645 | 7.891176 | 281.7218 | 11196.76 | 44.4177 | 7.76E-03 | 0.149318 | 276.4915 |
|  | 1716.855 | 96.65855 | 7.185587 | 263.692 | 11360.31 | 37.70846 | 6.56E-03 | 0.151127 | 273.045 |
|  | 1728.802 | 72.31671 | 5.469742 | 455.5573 | 12979.09 | 23.30616 | 4.91E-03 | 0.133199 | 65.70104 |
|  | 1740.783 | 89.30603 | 6.987908 | 169.0058 | 11098.09 | 42.89571 | 6.06E-03 | 0.156854 | 293.6663 |
|  | 1745.867 | 80.97126 | 6.348495 | 231.2815 | 9538.481 | 34.30041 | 5.49E-03 | 0.183034 | 191.3869 |
|  | 1855.841 | 91.01911 | 9.930954 | 1139.744 | 10573.02 | 48.17489 | 6.18E-03 | 0.175526 | 65.82633 |
|  | 2040.006 | 840.3562 | 126.0287 | 12763.49 | 12648.65 | 425.8722 | 5.70E-02 | 0.161282 | 2650.571 |
|  | 2158.051 | 65.22895 | 11.16627 | 644.7492 | 13790.97 | 34.16151 | 4.43E-03 | 0.156483 | 63.72501 |
|  | 2206.092 | 70.99817 | 12.82434 | 316.112 | 13656.17 | 38.58574 | 4.82E-03 | 0.161545 | 188.8303 |
|  | 2705.176 | 41.34664 | 18.18083 | 430.3298 | 15181.63 | 25.66481 | 2.81E-03 | 0.178187 | 60.66202 |
|  | 3312.32 | 68.63496 | 110.4075 | 462.3229 | 16698.1 | 55.94696 | 4.66E-03 | 0.198365 | 252.3477 |
|  |  |  |  |  |  |  |  |  |  |
| ID | m/z | Intens. | SN | Quality Fac. | Res. | Area | Rel. Intens. | FWHM | Chi^2 |
| 83 | 765.5939 | 65.66818 | 21.16958 | 99.65727 | 9081.613 | 9.226639 | 2.67E-03 | 8.43E-02 | 151.558 |
|  | 842.5094 | 81.41124 | 29.34307 | 592.5295 | 6658.509 | 17.51434 | 3.32E-03 | 0.126531 | 42.15721 |
|  | 1059.549 | 109.3346 | 30.63033 | 1383.593 | 8496.858 | 26.19589 | 4.45E-03 | 0.124699 | 36.54368 |
|  | 1065.514 | 2264.875 | 634.9428 | 3495.186 | 7280.732 | 633.9114 | 9.23E-02 | 0.146347 | 14546.49 |
|  | 1114.487 | 324.4878 | 93.17399 | 687.7086 | 8612.055 | 82.06697 | 1.32E-02 | 0.12941 | 705.6304 |
|  | 1146.472 | 272.8073 | 66.82585 | 1009.367 | 9593.35 | 68.56958 | 1.11E-02 | 0.119507 | 346.8263 |
|  | 1193.606 | 2038.421 | 409.2175 | 5479.27 | 8220.585 | 637.7131 | 8.30E-02 | 0.145197 | 12071.8 |
|  | 1248.658 | 116.6597 | 9.118904 | 1558.366 | 7373.489 | 40.3649 | 4.75E-03 | 0.169344 | 45.89839 |
|  | 1259.663 | 2984.46 | 149.4233 | 26622.9 | 8001.384 | 1045.425 | 0.121569 | 0.157431 | 6018.36 |
|  | 1263.657 | 320.3468 | 14.09056 | 1528.669 | 6049.552 | 146.9719 | 1.30E-02 | 0.208884 | 415.5067 |
|  | 1278.713 | 24549.51 | 754.987 | 99352.11 | 6377.564 | 11453.98 | 1 | 0.200502 | 227042 |
|  | 1290.7 | 436.5615 | 10.8272 | 92.87755 | 7930.261 | 165.4589 | 1.78E-02 | 0.162756 | 14434.13 |
|  | 1292.649 | 11567.72 | 278.4039 | 185601.1 | 7326.649 | 4691.26 | 0.471199 | 0.176431 | 29371.52 |
|  | 1296.646 | 1186.341 | 26.80896 | 2179.82 | 8943.935 | 375.7782 | 0.048324 | 0.144975 | 3514.227 |
|  | 1308.646 | 6349.168 | 139.9639 | 40576.51 | 7947.96 | 2417.131 | 0.258627 | 0.164652 | 26699.53 |
|  | 1320.657 | 381.435 | 10.16454 | 196.3664 | 9214.19 | 127.2167 | 1.55E-02 | 0.143329 | 4558.107 |
|  | 1324.641 | 9313.404 | 268.6244 | 57110.21 | 7611.695 | 3653.241 | 0.379372 | 0.174027 | 55758.77 |
|  | 1335.727 | 289.9236 | 10.47902 | 1730.639 | 9956.151 | 94.35471 | 1.18E-02 | 0.134161 | 269.7303 |
|  | 1340.634 | 132.7268 | 5.417426 | 656.8185 | 10699.78 | 40.46656 | 5.41E-03 | 0.125295 | 151.2795 |
|  | 1432.792 | 76.6507 | 13.29803 | 501.0255 | 7960.555 | 33.68273 | 3.12E-03 | 0.179986 | 77.73867 |
|  | 1448.765 | 151.5601 | 25.05164 | 684.2805 | 9977.921 | 52.01827 | 6.17E-03 | 0.145197 | 202.1631 |
|  | 1464.754 | 99.26165 | 16.59725 | 688.8374 | 12602.96 | 30.0409 | 4.04E-03 | 0.116223 | 74.62218 |
|  | 1473.866 | 110.6075 | 18.24119 | 893.5157 | 9418.2 | 41.91797 | 4.51E-03 | 0.156491 | 87.4374 |
|  | 1480.74 | 175.9753 | 29.28403 | 1402.886 | 10491.24 | 63.04431 | 7.17E-03 | 0.141141 | 130.4973 |
|  | 1487.888 | 286.3929 | 46.65104 | 1912.74 | 10809.21 | 97.3641 | 1.17E-02 | 0.13765 | 264.096 |
|  | 1534.852 | 75.96566 | 14.14314 | 441.0218 | 13947.17 | 26.78667 | 3.09E-03 | 0.110048 | 82.87985 |
|  | 1562.853 | 505.6056 | 112.1854 | 2374.685 | 11789.7 | 189.8659 | 2.06E-02 | 0.132561 | 1145.87 |
|  | 1699.92 | 98.69695 | 10.7891 | 603.9145 | 14386.36 | 30.80047 | 4.02E-03 | 0.118162 | 115.2807 |
|  | 1739.925 | 882.7901 | 70.01006 | 19511.84 | 10089.65 | 449.8887 | 3.60E-02 | 0.172446 | 1122.543 |
|  | 1741.968 | 205.8996 | 16.68605 | 1001.42 | 6438.177 | 160.1764 | 8.39E-03 | 0.270569 | 468.0583 |
|  | 1757.95 | 14259.14 | 1385.055 | 23340.72 | 7478.068 | 10313.83 | 0.580832 | 0.235081 | 96721.45 |
|  | 1785.931 | 177.8547 | 26.0088 | 1004.866 | 11483.78 | 80.26648 | 7.24E-03 | 0.155518 | 272.0677 |
|  | 1814.95 | 98.14663 | 25.86175 | 754.6155 | 12592.84 | 44.70682 | 4.00E-03 | 0.144125 | 116.2985 |
|  | 1879.944 | 84.41747 | 20.81774 | 788.9056 | 13168.61 | 40.03892 | 3.44E-03 | 0.142759 | 84.65194 |
|  | 1917.959 | 321.7874 | 87.70794 | 8243.113 | 10382.9 | 175.8441 | 1.31E-02 | 0.184723 | 484.9356 |
|  | 1965.95 | 459.4565 | 157.7675 | 3197.474 | 13381.03 | 219.8481 | 1.87E-02 | 0.146921 | 1489.486 |
|  | 1981.959 | 60.10033 | 21.55548 | 906.4024 | 12005.27 | 30.66652 | 2.45E-03 | 0.165091 | 43.06747 |
|  | 2056.142 | 239.4253 | 99.51193 | 3469.387 | 11513.4 | 135.4529 | 9.75E-03 | 0.178587 | 473.0088 |
|  | 2084.131 | 112.1374 | 50.30167 | 1377.714 | 13708.03 | 55.33414 | 4.57E-03 | 0.152037 | 132.0681 |
|  | 2191.125 | 84.52018 | 39.18773 | 927.9159 | 16217.96 | 41.03056 | 3.44E-03 | 0.135105 | 82.10524 |
|  | 2199.186 | 243.4459 | 110.7266 | 1148.156 | 15901.74 | 108.8358 | 9.92E-03 | 0.138298 | 695.0062 |
|  | 2221.16 | 73.21284 | 32.3197 | 832.5863 | 13263.21 | 38.52287 | 2.98E-03 | 0.167468 | 72.96977 |
|  | 2560.249 | 61.01796 | 21.31471 | 868.7246 | 12914.52 | 38.55437 | 2.49E-03 | 0.198246 | 63.01488 |
|  | 2578.293 | 115.2133 | 37.16961 | 1716.71 | 13583.56 | 74.06892 | 4.69E-03 | 0.18981 | 121.5589 |
|  | 2756.293 | 528.2266 | 85.55541 | 1644.177 | 11624.13 | 452.2986 | 2.15E-02 | 0.237118 | 3066.13 |
|  | 2763.591 | 197.3308 | 30.57963 | 1459.054 | 9509.319 | 210.4463 | 8.04E-03 | 0.290619 | 525.5115 |
|  | 2804.282 | 1921.161 | 367.2707 | 4198.091 | 12288.59 | 1734.215 | 7.83E-02 | 0.228202 | 15406.29 |
|  | 2820.29 | 410.2514 | 89.75933 | 4692.18 | 13591.68 | 316.3686 | 1.67E-02 | 0.207501 | 1572.86 |
|  | 2960.397 | 55.77845 | 31.55892 | 673.1472 | 19239.69 | 39.42318 | 2.27E-03 | 0.153869 | 87.51773 |
|  |  |  |  |  |  |  |  |  |  |
| ID | m/z | Intens. | SN | Quality Fac. | Res. | Area | Rel. Intens. | FWHM | Chi^2 |
| 245 | 774.4158 | 61.07112 | 11.97276 | 198.0672 | 7021.466 | 11.12577 | 0.01572 | 0.110293 | 61.12307 |
|  | 804.2792 | 79.07667 | 14.93236 | 521.4655 | 7600.404 | 14.06858 | 2.04E-02 | 0.105821 | 36.31668 |
|  | 832.4832 | 54.20438 | 9.713738 | 349.2914 | 7810.626 | 9.735709 | 1.40E-02 | 0.106583 | 27.68208 |
|  | 838.4615 | 81.19784 | 14.47057 | 337.6897 | 8616.141 | 13.61096 | 2.09E-02 | 9.73E-02 | 62.9735 |
|  | 856.5248 | 117.4247 | 21.4571 | 290.958 | 12872.29 | 14.47068 | 3.02E-02 | 6.65E-02 | 143.9756 |
|  | 870.5454 | 754.2021 | 144.9019 | 602.6186 | 10448.01 | 116.597 | 0.194138 | 8.33E-02 | 4747.308 |
|  | 934.5411 | 86.41644 | 18.36762 | 419.8128 | 10833.99 | 14.44303 | 0.022244 | 8.63E-02 | 65.71306 |
|  | 950.5502 | 71.34505 | 15.3156 | 636.9868 | 7316.407 | 17.10864 | 1.84E-02 | 0.12992 | 31.39977 |
|  | 973.5271 | 30.01685 | 6.478312 | 245.6121 | 11445.19 | 5.172027 | 7.73E-03 | 8.51E-02 | 13.23127 |
|  | 982.4484 | 165.2315 | 36.13283 | 313.4486 | 12613.1 | 29.7205 | 4.25E-02 | 7.79E-02 | 324.7036 |
|  | 1006.422 | 54.65222 | 11.82825 | 310.8607 | 9896.691 | 11.5705 | 1.41E-02 | 0.101693 | 39.94956 |
|  | 1011.652 | 107.264 | 23.37024 | 818.9067 | 9490.443 | 21.45732 | 2.76E-02 | 0.106597 | 50.83529 |
|  | 1057.519 | 24.98716 | 5.192897 | 155.4901 | 11210.34 | 5.624946 | 6.43E-03 | 9.43E-02 | 10.88586 |
|  | 1060.565 | 20.72219 | 4.407945 | 146.65 | 8697.858 | 5.620379 | 5.33E-03 | 0.121934 | 6.612644 |
|  | 1092.516 | 105.5299 | 22.11582 | 524.9311 | 10119.34 | 25.70522 | 2.72E-02 | 0.107963 | 99.43689 |
|  | 1219.707 | 26.97009 | 5.634094 | 293.424 | 13105 | 7.504465 | 6.94E-03 | 9.31E-02 | 10.53539 |
|  | 1277.731 | 450.4628 | 71.85514 | 10096.97 | 11092.58 | 110.7809 | 0.115953 | 0.115188 | 458.6789 |
|  | 1303.695 | 1845.974 | 270.0627 | 8317.052 | 9975.663 | 522.1568 | 0.475171 | 0.130688 | 17244.02 |
|  | 1318.76 | 42.93988 | 6.664159 | 191.5853 | 8717.088 | 14.6921 | 1.11E-02 | 0.151284 | 52.21616 |
|  | 1325.681 | 64.56899 | 10.3042 | 85.79674 | 9497.551 | 20.95913 | 1.66E-02 | 0.139581 | 293.6549 |
|  | 1327.744 | 198.0929 | 32.32126 | 1621.1 | 10744.39 | 60.4685 | 5.10E-02 | 0.123576 | 353.1773 |
|  | 1338.838 | 127.6257 | 22.21973 | 666.4965 | 7971.237 | 47.83531 | 3.29E-02 | 0.167959 | 142.4327 |
|  | 1339.655 | 57.82701 | 10.13723 | 197.8134 | 10332.5 | 16.1615 | 1.49E-02 | 0.129655 | 84.63852 |
|  | 1417.875 | 1408.316 | 338.1189 | 12188.53 | 11367.54 | 416.2299 | 0.362513 | 0.12473 | 8145.743 |
|  | 1424.668 | 43.46132 | 10.44189 | 174.7481 | 12348.7 | 13.58841 | 1.12E-02 | 0.11537 | 54.94298 |
|  | 1439.797 | 34.6317 | 8.533855 | 332.9713 | 7853.818 | 17.19169 | 8.91E-03 | 0.183324 | 28.47617 |
|  | 1523.807 | 15.5854 | 5.385474 | 145.5484 | 9116.709 | 7.182564 | 4.01E-03 | 0.167144 | 7.943886 |
|  | 1526.735 | 16.88586 | 5.732967 | 60.70302 | 16976.36 | 4.878635 | 4.35E-03 | 8.99E-02 | 19.07066 |
|  | 1530.766 | 35.29207 | 12.65526 | 518.9928 | 17627.21 | 13.01162 | 9.08E-03 | 8.68E-02 | 18.4567 |
|  | 1584.746 | 35.08137 | 11.69772 | 597.0211 | 12082.81 | 12.07213 | 9.03E-03 | 0.131157 | 14.24835 |
|  | 1598.786 | 20.26813 | 6.73539 | 384.7621 | 11093.31 | 8.231561 | 5.22E-03 | 0.144122 | 4.992013 |
|  | 1647.996 | 9.600153 | 3.30307 | 78.41489 | 10599.31 | 4.894317 | 2.47E-03 | 0.155481 | 6.44696 |
|  | 1666.011 | 122.9739 | 44.05651 | 3929.729 | 13911.15 | 44.62355 | 3.17E-02 | 0.119761 | 106.6407 |
|  | 1687.974 | 76.03166 | 27.96654 | 1399.683 | 14221.72 | 24.74162 | 1.96E-02 | 0.11869 | 75.81828 |
|  | 1716.857 | 51.093 | 19.20559 | 1374.671 | 13029.38 | 19.66529 | 1.32E-02 | 0.131768 | 19.1286 |
|  | 1802.008 | 20.43673 | 7.7205 | 260.8736 | 12277.25 | 9.487529 | 5.26E-03 | 0.146776 | 16.34573 |
|  | 1837.928 | 14.28874 | 6.772489 | 208.8086 | 15886.41 | 4.452975 | 3.68E-03 | 0.115692 | 5.058117 |
|  | 1947.916 | 9.057234 | 6.917001 | 191.8075 | 16019.74 | 3.619016 | 2.33E-03 | 0.121595 | 3.455889 |
|  | 2225.16 | 13.34472 | 8.071005 | 555.4611 | 12266.17 | 7.182663 | 3.44E-03 | 0.181406 | 3.408709 |
|  | 2232.647 | 36.22731 | 20.78046 | 382.144 | 14231.24 | 18.27696 | 9.33E-03 | 0.156883 | 41.91534 |
|  | 2273.194 | 146.9916 | 88.82338 | 3407.689 | 16291.12 | 64.10217 | 3.78E-02 | 0.139536 | 249.4571 |
|  | 2289.194 | 151.1139 | 96.45028 | 1324.012 | 18800.29 | 57.4282 | 3.89E-02 | 0.121764 | 596.4237 |
|  | 2342.975 | 12.9709 | 10.34815 | 223.8983 | 18080.16 | 6.400171 | 3.34E-03 | 0.129588 | 8.295991 |
|  | 2501.256 | 80.36716 | 121.9787 | 1736.16 | 16767.12 | 40.70497 | 2.07E-02 | 0.149176 | 159.5594 |
|  | 2565.18 | 117.3004 | 177.7807 | 2039.494 | 18781.59 | 60.20297 | 3.02E-02 | 0.136579 | 339.9345 |
|  | 2705.182 | 140.5805 | 390.8424 | 2611.954 | 17970.88 | 73.52491 | 3.62E-02 | 0.150531 | 525.0178 |
|  | 3223.42 | 6.211709 | 56.82482 | 146.1117 | 24321.76 | 3.73533 | 1.60E-03 | 0.132532 | 5.197774 |
|  | 3312.443 | 260.7008 | 1112.296 | 364.2739 | 18117.22 | 192.6621 | 6.71E-02 | 0.182834 | 4382.33 |
|  |  |  |  |  |  |  |  |  |  |
| ID | m/z | Intens. | SN | Quality Fac. | Res. | Area | Rel. Intens. | FWHM | Chi^2 |
| 363 | 743.36 | 526.9311 | 96.58455 | 1770.899 | 7800.274 | 76.74943 | 8.03E-02 | 0.095299 | 1432.743 |
|  | 804.2624 | 52.40292 | 9.880651 | 216.2751 | 7482.022 | 11.33921 | 7.99E-03 | 0.107493 | 49.0488 |
|  | 838.4728 | 26.95354 | 4.841838 | 77.67499 | 8013.771 | 5.281355 | 4.11E-03 | 0.104629 | 23.9428 |
|  | 856.5254 | 164.6392 | 30.62913 | 1679.365 | 9584.906 | 25.1426 | 2.51E-02 | 8.94E-02 | 84.22231 |
|  | 870.5438 | 1587.617 | 308.2442 | 6291.212 | 8769.35 | 276.3885 | 0.242 | 9.93E-02 | 7468.187 |
|  | 885.431 | 179.9879 | 36.59292 | 2122.638 | 8882.941 | 31.0315 | 2.74E-02 | 9.97E-02 | 149.5836 |
|  | 923.4841 | 65.05944 | 14.55742 | 656.5811 | 11438.47 | 11.68066 | 9.92E-03 | 8.07E-02 | 30.55051 |
|  | 944.5266 | 18.1991 | 4.267862 | 52.5083 | 10458.6 | 3.851212 | 2.77E-03 | 9.03E-02 | 18.21279 |
|  | 945.5313 | 21.8041 | 5.10874 | 173.4249 | 9070.231 | 4.747618 | 3.32E-03 | 0.104246 | 11.41246 |
|  | 949.5277 | 134.5692 | 31.82972 | 1076.648 | 10854.83 | 22.2727 | 2.05E-02 | 0.087475 | 101.7804 |
|  | 950.5355 | 28.29596 | 6.712857 | 93.42202 | 11717.53 | 5.185812 | 4.31E-03 | 0.081121 | 32.51305 |
|  | 1029.621 | 36.69666 | 8.181444 | 406.2041 | 10615.02 | 6.747798 | 5.59E-03 | 9.70E-02 | 10.69228 |
|  | 1038.582 | 53.23101 | 11.9255 | 868.4483 | 8022.628 | 13.06607 | 8.11E-03 | 0.129457 | 16.78802 |
|  | 1058.613 | 20.01006 | 4.665767 | 59.27666 | 7697.163 | 5.423403 | 3.05E-03 | 0.137533 | 14.75324 |
|  | 1078.599 | 22.43414 | 5.449466 | 87.11967 | 9062.796 | 5.302869 | 3.42E-03 | 0.119014 | 17.58145 |
|  | 1122.615 | 29.11561 | 7.081415 | 333.9902 | 12854.7 | 5.354592 | 4.44E-03 | 8.73E-02 | 8.819981 |
|  | 1205.67 | 130.9231 | 26.6516 | 1272.479 | 10237.78 | 35.00282 | 0.019957 | 0.117767 | 284.8404 |
|  | 1207.702 | 48.49277 | 9.911871 | 236.1087 | 9391.707 | 15.58886 | 7.39E-03 | 0.128592 | 57.85725 |
|  | 1222.687 | 1782.189 | 343.5169 | 16161.66 | 10098.3 | 459.1174 | 0.271658 | 0.121079 | 8426.529 |
|  | 1263.72 | 102.4307 | 18.93195 | 522.4108 | 10684.13 | 26.94322 | 0.015613 | 0.11828 | 105.243 |
|  | 1267.675 | 124.9772 | 23.02517 | 2845.107 | 10463.35 | 32.46071 | 0.01905 | 0.121154 | 70.30303 |
|  | 1278.715 | 68.64204 | 12.48418 | 617.567 | 10261.12 | 19.29524 | 1.05E-02 | 0.124618 | 37.88341 |
|  | 1284.773 | 222.8868 | 41.13913 | 4006.772 | 11594.88 | 57.63364 | 3.40E-02 | 0.110805 | 296.9154 |
|  | 1327.733 | 46.32724 | 8.422748 | 216.9913 | 11054.05 | 12.83713 | 7.06E-03 | 0.120113 | 51.49698 |
|  | 1350.768 | 187.8675 | 33.79238 | 5946.038 | 10225.1 | 53.7785 | 2.86E-02 | 0.132103 | 149.9842 |
|  | 1395.747 | 65.52049 | 11.63991 | 628.5683 | 14661.53 | 16.04869 | 9.99E-03 | 9.52E-02 | 36.43559 |
|  | 1410.711 | 23.86337 | 4.24452 | 137.1478 | 9180.934 | 9.17678 | 3.64E-03 | 0.153657 | 11.64086 |
|  | 1424.677 | 39.90928 | 7.071804 | 217.6 | 10562.78 | 15.37532 | 6.08E-03 | 0.134877 | 38.95677 |
|  | 1425.723 | 26.55636 | 4.567693 | 73.53138 | 15957.4 | 6.579994 | 4.05E-03 | 8.93E-02 | 41.704 |
|  | 1428.759 | 29.92587 | 5.351609 | 241.9884 | 11879.35 | 10.53963 | 4.56E-03 | 0.120273 | 23.36823 |
|  | 1442.849 | 62.60652 | 11.0939 | 558.5467 | 11247.42 | 20.46209 | 9.54E-03 | 0.128283 | 39.27888 |
|  | 1448.846 | 78.9644 | 14.16224 | 1359.54 | 12473.72 | 22.51956 | 1.20E-02 | 0.116152 | 52.72876 |
|  | 1470.837 | 38.3116 | 7.090607 | 190.4173 | 14635.76 | 9.324538 | 5.84E-03 | 0.100496 | 27.3161 |
|  | 1489.721 | 113.7813 | 22.35553 | 2090.899 | 13095.21 | 36.56568 | 1.73E-02 | 0.113761 | 116.1639 |
|  | 1509.802 | 164.0954 | 34.31554 | 5847.683 | 11451.77 | 55.09969 | 2.50E-02 | 0.13184 | 83.8685 |
|  | 1524.857 | 100.4253 | 21.9469 | 2083.611 | 11860.8 | 33.79884 | 1.53E-02 | 0.128563 | 51.37295 |
|  | 1530.764 | 61.26484 | 13.2893 | 73.71186 | 13584.11 | 18.00644 | 9.34E-03 | 0.112688 | 455.624 |
|  | 1532.814 | 273.4586 | 60.62935 | 6103.017 | 12680.27 | 85.4104 | 4.17E-02 | 0.120882 | 507.1213 |
|  | 1598.778 | 81.31592 | 17.92545 | 2497.288 | 14942.66 | 27.63485 | 1.24E-02 | 0.106994 | 28.67126 |
|  | 1604.927 | 22.5758 | 4.955753 | 141.2566 | 10180.52 | 11.12932 | 3.44E-03 | 0.157647 | 14.44713 |
|  | 1626.791 | 19.54853 | 4.248438 | 204.4291 | 7724.254 | 10.8611 | 2.98E-03 | 0.210608 | 8.684855 |
|  | 1637.875 | 42.46257 | 9.054092 | 287.3378 | 12285.69 | 15.3426 | 6.47E-03 | 0.133316 | 39.54223 |
|  | 1643.78 | 523.4169 | 110.8392 | 8814.789 | 12162 | 188.6933 | 7.98E-02 | 0.135157 | 1688.122 |
|  | 1654.455 | 72.35977 | 15.76564 | 1089.114 | 10481.43 | 34.72454 | 1.10E-02 | 0.157846 | 39.99748 |
|  | 1659.762 | 22.37035 | 4.871903 | 102.3311 | 11996.87 | 11.92658 | 3.41E-03 | 0.13835 | 18.94685 |
|  | 1675.798 | 52.58616 | 11.28789 | 477.7799 | 15485.15 | 19.24388 | 8.02E-03 | 0.10822 | 42.52446 |
|  | 1694.816 | 45.48483 | 9.838245 | 541.9681 | 14520.69 | 16.32589 | 6.93E-03 | 0.116717 | 26.22883 |
|  | 1710.821 | 402.8302 | 92.03179 | 14681.72 | 14515.92 | 137.6586 | 6.14E-02 | 0.117858 | 568.242 |
|  | 1794.897 | 56.74723 | 21.66127 | 2964.39 | 14085.02 | 21.05501 | 8.65E-03 | 0.127433 | 31.30493 |
|  | 1801.947 | 16.06462 | 6.23518 | 296.0464 | 12576.27 | 6.670902 | 2.45E-03 | 0.143281 | 5.723623 |
|  | 1852.773 | 16.05146 | 7.492222 | 364.5638 | 16678.13 | 6.082221 | 2.45E-03 | 0.11109 | 5.65821 |
|  | 2047.058 | 12.20024 | 9.304478 | 184.6213 | 15770.76 | 5.482477 | 1.86E-03 | 0.129801 | 8.164028 |
|  | 2075.97 | 13.36953 | 10.80151 | 502.5548 | 13907.6 | 6.781237 | 2.04E-03 | 0.149269 | 3.660764 |
|  | 2225.12 | 19.07762 | 12.6447 | 342.6993 | 20747.79 | 7.58356 | 2.91E-03 | 0.107246 | 10.69708 |
|  | 2233.089 | 36.42576 | 23.56464 | 427.172 | 17307.29 | 16.22483 | 5.55E-03 | 0.129026 | 35.36553 |
|  | 2379.147 | 678.7217 | 780.2822 | 6906.823 | 18644.48 | 302.0827 | 0.103457 | 0.127606 | 5271.038 |
|  | 3102.603 | 5.63689 | 74.50936 | 482.3789 | 33387.06 | 2.270167 | 8.59E-04 | 0.092928 | 3.623766 |
|  |  |  |  |  |  |  |  |  |  |
| ID | m/z | Intens. | SN | Quality Fac. | Res. | Area | Rel. Intens. | FWHM | Chi^2 |
| 589 | 765.5748 | 174.0027 | 43.3707 | 113.7863 | 10705 | 22.41414 | 3.08E-02 | 7.15E-02 | 745.7896 |
|  | 767.5767 | 101.7618 | 25.19017 | 226.1175 | 8744.814 | 17.97238 | 1.80E-02 | 8.78E-02 | 179.4179 |
|  | 774.4251 | 82.17988 | 20.97377 | 1922.445 | 8032.353 | 12.91416 | 1.45E-02 | 9.64E-02 | 10.65104 |
|  | 782.438 | 15.97775 | 4.109395 | 55.86381 | 5766.882 | 3.840645 | 2.82E-03 | 0.135678 | 9.709435 |
|  | 804.2831 | 20.63446 | 4.98257 | 138.6636 | 6747.894 | 4.594473 | 3.65E-03 | 0.11919 | 7.881077 |
|  | 812.3212 | 205.4743 | 45.36078 | 125.9986 | 13621.4 | 20.93289 | 0.036325 | 5.96E-02 | 894.9332 |
|  | 814.2986 | 45.68645 | 10.71028 | 126.4305 | 11222.99 | 6.307442 | 8.08E-03 | 7.26E-02 | 51.09792 |
|  | 836.4221 | 295.948 | 64.69953 | 3428.174 | 8492.539 | 48.63846 | 0.05232 | 0.098489 | 265.7937 |
|  | 838.4515 | 57.7221 | 12.17838 | 284.8455 | 9571.884 | 8.700529 | 1.02E-02 | 8.76E-02 | 39.17259 |
|  | 847.4662 | 24.90756 | 5.660658 | 85.22475 | 6316.657 | 5.67639 | 4.40E-03 | 0.134164 | 27.2034 |
|  | 856.523 | 67.25359 | 15.87203 | 552.3671 | 8796.649 | 12.08382 | 1.19E-02 | 9.74E-02 | 29.61057 |
|  | 864.4947 | 26.02186 | 6.621062 | 113.7848 | 7978.065 | 4.568995 | 4.60E-03 | 0.108359 | 20.37887 |
|  | 870.538 | 485.4209 | 127.1206 | 4606.698 | 8509.068 | 84.56907 | 8.58E-02 | 0.102307 | 907.3351 |
|  | 950.5305 | 54.87846 | 19.5938 | 209.8616 | 13727.04 | 7.485978 | 9.70E-03 | 6.92E-02 | 46.33066 |
|  | 1028.573 | 157.6232 | 53.29471 | 2370.522 | 10755.1 | 31.77773 | 2.79E-02 | 9.56E-02 | 108.1817 |
|  | 1046.145 | 99.48927 | 34.10467 | 78.26209 | 13690.53 | 13.97075 | 1.76E-02 | 7.64E-02 | 411.0492 |
|  | 1048.158 | 53.52308 | 19.19734 | 76.06211 | 13257.5 | 7.606308 | 9.46E-03 | 7.91E-02 | 134.5757 |
|  | 1186.679 | 922.2745 | 373.2932 | 5960.188 | 10345.57 | 214.7097 | 0.163046 | 0.114704 | 4916.102 |
|  | 1219.715 | 16.83978 | 7.065287 | 130.4467 | 9088.777 | 5.197265 | 2.98E-03 | 0.1342 | 7.57452 |
|  | 1282.002 | 16.47055 | 6.312096 | 92.4342 | 8574.657 | 4.815554 | 2.91E-03 | 0.149511 | 14.24844 |
|  | 1303.678 | 12.23188 | 4.601881 | 61.04418 | 11402.88 | 3.226219 | 2.16E-03 | 0.114329 | 10.04696 |
|  | 1313.659 | 66.35551 | 25.3706 | 1413.008 | 12476.15 | 17.89146 | 0.011731 | 0.105294 | 34.18876 |
|  | 1319.657 | 14.68643 | 5.643883 | 100.9829 | 13611.45 | 3.774243 | 2.60E-03 | 9.70E-02 | 10.61377 |
|  | 1327.736 | 81.18521 | 31.35054 | 616.116 | 11179.01 | 21.84732 | 0.014353 | 0.118771 | 59.15409 |
|  | 1431.705 | 61.0811 | 27.49321 | 1495.546 | 12313.24 | 17.65758 | 1.08E-02 | 0.116274 | 18.78488 |
|  | 1447.716 | 47.54118 | 21.34885 | 320.4859 | 17780.81 | 12.74815 | 8.40E-03 | 8.14E-02 | 40.68136 |
|  | 1515.772 | 10.07329 | 5.208852 | 73.79996 | 11352.64 | 3.571674 | 1.78E-03 | 0.133517 | 9.734781 |
|  | 1670.917 | 10.22882 | 4.918902 | 139.4476 | 11840.09 | 3.890653 | 1.81E-03 | 0.141124 | 4.144234 |
|  | 1720.904 | 3677.627 | 1593.413 | 12403.86 | 11943.87 | 1500.3 | 0.650157 | 0.144083 | 46296.56 |
|  | 1736.858 | 34.11381 | 16.13466 | 559.9927 | 12649.13 | 12.57217 | 6.03E-03 | 0.137311 | 17.97196 |
|  | 1752.843 | 28.8511 | 14.62313 | 671.6789 | 13764.66 | 10.44255 | 5.10E-03 | 0.127344 | 8.886744 |
|  | 1784.843 | 10.68279 | 6.526183 | 118.8308 | 13944.04 | 4.60629 | 1.89E-03 | 0.128 | 4.929609 |
|  | 1798.933 | 21.98246 | 13.38247 | 334.1176 | 11615.06 | 10.31425 | 3.89E-03 | 0.154879 | 12.96437 |
|  | 1829.957 | 5656.518 | 3442.018 | 7288.517 | 11894.34 | 2633.589 | 1 | 0.153851 | 118240.9 |
|  | 2015.991 | 18.59245 | 28.98654 | 564.4735 | 14247.88 | 8.317489 | 3.29E-03 | 0.141494 | 6.412927 |
|  | 2048.074 | 6.055244 | 9.796253 | 111.2221 | 15425.2 | 3.251269 | 1.07E-03 | 0.132775 | 3.737987 |
|  | 2233.094 | 5.605686 | 8.260366 | 130.237 | 14519.64 | 2.878323 | 9.91E-04 | 0.153798 | 2.762069 |
